# *KIBRA (WWC1)* Is a Metastasis Suppressor Gene Affected by Chromosome 5q Loss in Triple-Negative Breast Cancer

**DOI:** 10.1016/j.celrep.2018.02.095

**Published:** 2018-03-20

**Authors:** Jennifer F. Knight, Vanessa Y.C. Sung, Elena Kuzmin, Amber L. Couzens, Danielle A. de Verteuil, Colin D.H. Ratcliffe, Paula P. Coelho, Radia M. Johnson, Payman Samavarchi-Tehrani, Tina Gruosso, Harvey W. Smith, Wontae Lee, Sadiq M. Saleh, Dongmei Zuo, Hong Zhao, Marie-Christine Guiot, Ryan R. Davis, Jeffrey P. Gregg, Christopher Moraes, Anne-Claude Gingras, Morag Park

**Affiliations:** 1 Goodman Cancer Research Centre, McGill University, Montreal, QC H3G 0B1, Canada; 2 Department of Biochemistry, McGill University, Montreal, QC H2W 1S6, Canada; 3 Lunenfeld-Tanenbaum Research Institute, Mount Sinai Hospital, Toronto, ON M5G 1X5, Canada; 4 Department of Oncology, McGill University, Montreal, QC H2W 1S6, Canada; 5 Department of Biomedical Engineering, McGill University, Montreal, QC H3A 2B4, Canada; 6 Montreal Neurological Institute, Department of Pathology, McGill University, Montreal, QC H3A 2B4, Canada; 7 Department of Pathology and Laboratory Medicine, University of California at Davis School of Medicine, Sacramento, CA 95817, USA; 8 Department of Chemical Engineering, McGill University, Montreal, QC H3A 0C5, Canada; 9 Department of Molecular Genetics, University of Toronto, Toronto, ON M5S 1A8, Canada

**Keywords:** *KIBRA*, *WWC1*, *PTPN14*, *YAP/TAZ*, mechanotransduction, RHOA signaling, triple-negative breast cancer, metastasis, tumorspheres, chr5q

## Abstract

Triple-negative breast cancers (TNBCs) display a complex spectrum of mutations and chromosomal aberrations. Chromosome 5q (5q) loss is detected in up to 70% of TNBCs, but little is known regarding the genetic drivers associated with this event. Here, we show somatic deletion of a region syntenic with human 5q33.2–35.3 in a mouse model of TNBC. Mechanistically, we identify *KIBRA* as a major factor contributing to the effects of 5q loss on tumor growth and metastatic progression. Re-expression of *KIBRA* impairs metastasis *in vivo* and inhibits tumorsphere formation by TNBC cells *in vitro*. *KIBRA* functions co-operatively with the protein tyrosine phosphatase *PTPN14* to trigger mechanotransduction-regulated signals that inhibit the nuclear localization of oncogenic transcriptional co-activators YAP/TAZ. Our results argue that the selective advantage produced by 5q loss involves reduced dosage of *KIBRA*, promoting oncogenic functioning of YAP/TAZ in TNBC.

## Highlights

• Reduced *KIBRA* expression is associated with chr 5q loss in breast cancer
• Restoring *Kibra* expression inhibits metastatic dissemination in mice
• KIBRA impairs the self-renewal capacity of triple-negative breast cancer cells
• KIBRA blocks mechanotransduction signals required for YAP/TAZ activation

## Graphical Abstract

**Figure:**
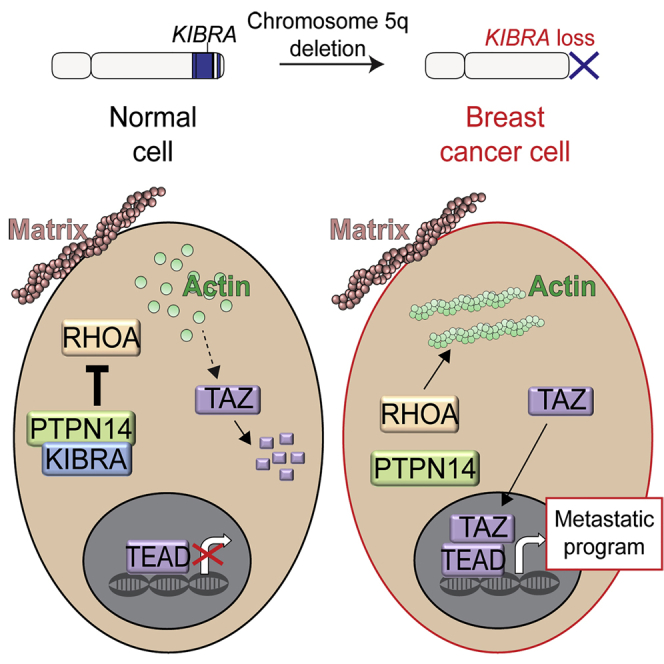

## Teaser

Triple-negative breast cancers (TNBCs) frequently lose chromosome 5q. Using a TNBC mouse model with spontaneous loss of a syntenic region, Knight et al. identify *KIBRA* as a metastasis suppressor. Mechanistically, KIBRA suppresses RHOA activation, impairing nuclear translocation of the oncogenes *YAP/TAZ*, which drive metastatic and cancer stem cell-like behavior.

## Introduction

Approximately 15% of patients with invasive breast cancer are diagnosed with triple-negative breast cancer (TNBC), defined by the absence of estrogen receptor (ER), progesterone receptor (PR), and human epidermal growth factor receptor 2 (HER2) expression (Foulkes et al., 2010). Because TNBC lacks an approved targeted therapy, the only systemic treatment is chemotherapy. Although this can induce a complete pathological response, TNBCs are associated with a high risk of early recurrence, and metastatic disease is virtually incurable (Denkert et al., 2017, Foulkes et al., 2010).

A concerted effort has been undertaken to understand the molecular basis of TNBC heterogeneity and discover actionable targets. Molecular subtyping based on gene expression has defined the majority of TNBCs as basal-like (49%–80%) (Denkert et al., 2017, Lehmann and Pietenpol, 2014, Rakha et al., 2009) or claudin-low (up to ∼30%) (Prat et al., 2010, Prat and Perou, 2011). Further studies have refined this classification into four subtypes: basal-like 1, basal-like 2, mesenchymal, and luminal androgen receptor (Lehmann et al., 2016). Integrating mutation status, gene expression, and copy number has shown that breast cancers segregate into 10 “integrative clusters” (Curtis et al., 2012). Most TNBCs (60%) fall into integrative cluster 10 (IntClust10), associated with an elevated 5-year risk of recurrence and frequent *TP53* mutations. Up to 70% of TNBCs also undergo deletions on the long arm of chromosome 5, spanning 5q11 to 5q35 (Johannsdottir et al., 2006, Natrajan et al., 2009, Turner et al., 2010). However, with few exceptions (Weigman et al., 2012), genes conferring selective pressure for 5q loss are relatively unknown.

Genetically engineered mouse models are powerful tools for deciphering breast cancer complexity (Cardiff et al., 2000, Herschkowitz et al., 2007). We have previously shown that mammary tumors driven by mouse mammary tumor virus (MMTV)-*Met* reflect human breast cancer subtypes, including basal-like (Ponzo et al., 2009), whereas conditional deletion of *Trp53* in this model (MMTV-*Met;Trp53fl/+;Cre*) induces mesenchymal tumors modeling the TNBC subtype claudin-low (Knight et al., 2013). Here we show that MMTV-*Met;Trp53fl/+;Cre* mammary tumors spontaneously lose a region on chromosome 11 that is syntenic with human 5q33.2–35.3. Using gene expression and functional analysis, we show that *WWC1* (*KIBRA)*, a scaffold protein and activator of the Hippo pathway located on 5q (Baumgartner et al., 2010, Genevet et al., 2010, Yu et al., 2010), has tumor- and metastasis-suppressive properties. Our data indicate a multifaceted role of KIBRA upstream of both canonical Hippo signaling and cytoskeletal cues that regulate the activity of the transcriptional coactivators YAP/TAZ.

## Results

### Chromosome 5q Loss, a Frequent Event in Human TNBC, Is Modeled in Mouse Mammary Tumors

**Figure 1:**
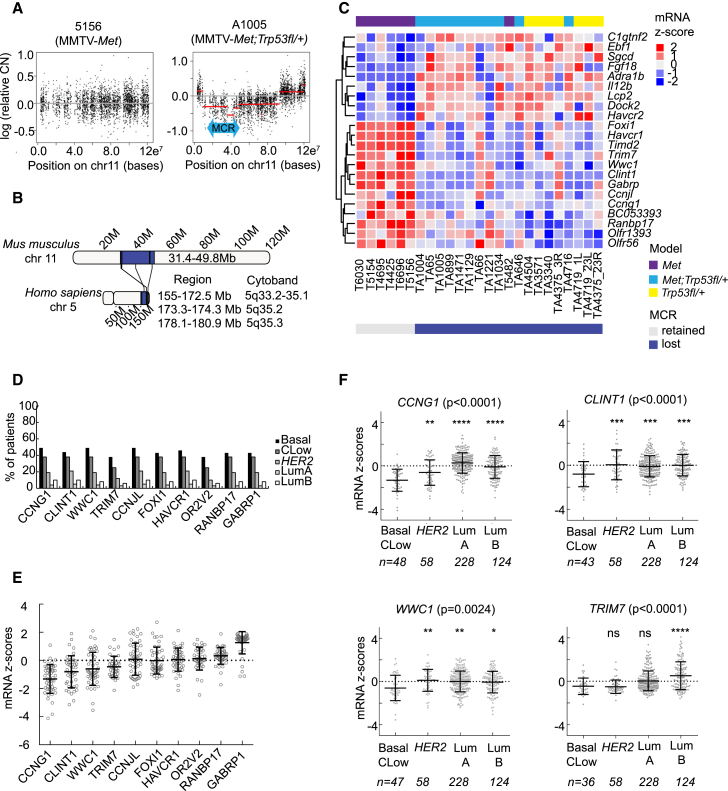
Loss of Heterozygosity in Mouse Mammary Tumors Mimics Chromosome 5q Loss, a Frequent Event in Human TNBC (A) Example aCGH profiles of chromosome (chr) 11 in MMTV-*Met* (5156) and MMTV-*Met;Trp53fl/+;Cre* (A1005) mammary tumors. Black dots indicate individual microarray probes and red lines segmented means for regions deviating from a log copy number change of 0. The blue arrow indicates a minimal common region (MCR) of loss from 18.9–49.8 Mb. (B) Alignment of the MCR with human chr 5q. (C) Heatmap showing significant differential expression among mouse model tumors, with decreased expression of 13 genes in tumors with loss of the MCR. (D) Frequency of hemizygous deletion for 10 of 11 genes across PAM50 and claudin-low (CLow) breast cancer subtypes in TCGA data. (E) TCGA mRNA *Z* scores for all 10 genes among basal and claudin-low tumors with hemizygous loss. (F) TCGA mRNA *Z* scores for all molecular subtypes. Asterisks indicate statistical significance for differences in mRNA levels between basal/claudin-low tumors with copy number loss and other PAM50 subtypes. n = number of patients. See also Figures S1 and S2 and Tables S2 and S3.

A powerful way to discover genes with causal roles in oncogenesis is to identify frequently altered genomic regions. Applying this approach to TNBC mouse models, we used array-comparative genomic hybridization (aCGH) to identify a region on chromosome 11 that is lost in 18 of 19 MMTV-*Met;Trp53fl/+;Cre* and *Trp53fl/+;Cre* tumors (Knight et al., 2013; Figures 1A and S1) but not MMTV-*Met* tumors (Ponzo et al., 2009), with one exception (Figure S1, 5482; Ponzo et al., 2009). Because the size of the affected region varied, we identified a minimal common region (MCR) of loss extending from 18.9 to 49.8 Mb (Figures 1A and S1).

Mouse chromosome 11:31.4–49.8 Mb is syntenic with human 5q33.2–35.3 (Figure 1B), which is frequently lost in TNBC (Table S1). We used The Cancer Genome Atlas breast cancer patient dataset (Cancer Genome Atlas Network, 2012) to explore the extent of 5q loss among basal and claudin-low subtypes, representing the majority of TNBCs. Segmental losses spanning the entire 5q arm were frequent, with 40%–55% of tumors showing loss of 5q33.2–35.3 (Figure S2A). To identify candidate tumor suppressor genes within 5q, we analyzed 88 mouse-human gene homologs from the syntenic region (Table S2). Because gene expression and copy number alteration are not always correlative, we analyzed their expression in our mouse models, finding 13 genes (orthologous to 11 unique human genes) that were significantly decreased in tumors with loss of the MCR (Figure 1C; Table S3). Analysis of copy number and expression data, available for 10 of these genes, confirmed their hemizygous deletion in 40%–50% of human claudin-low and basal breast cancers (Figure 1D), although only 4 of 10 had negative mRNA *Z* scores, consistent with decreased expression (Figure 1E). Furthermore, only *CCNG1*, *CLINT1*, and *WWC1* had significantly decreased expression in basal and claudin-low patients (Figure 1F). To corroborate our findings, we used the Cancer Cell Line Encyclopedia (CCLE) to analyze expression in human cell lines representing breast cancer subtypes. Although *CCNG1* mRNA levels were universally low irrespective of subtype, and *CLINT1* levels did not vary significantly, basal B (claudin-low) cell lines had significantly lower expression of *WWC1* (also known as *KIBRA*) (Figure S2B). This is consistent with a previous observation associating low *WWC1* expression with a claudin-low phenotype (Moleirinho et al., 2013).

### Depletion of *WWC1/KIBRA*, a 5q Gene, Increases the Metastatic Aggressivity of Mouse Breast Cancer Cells

**Figure 2:**
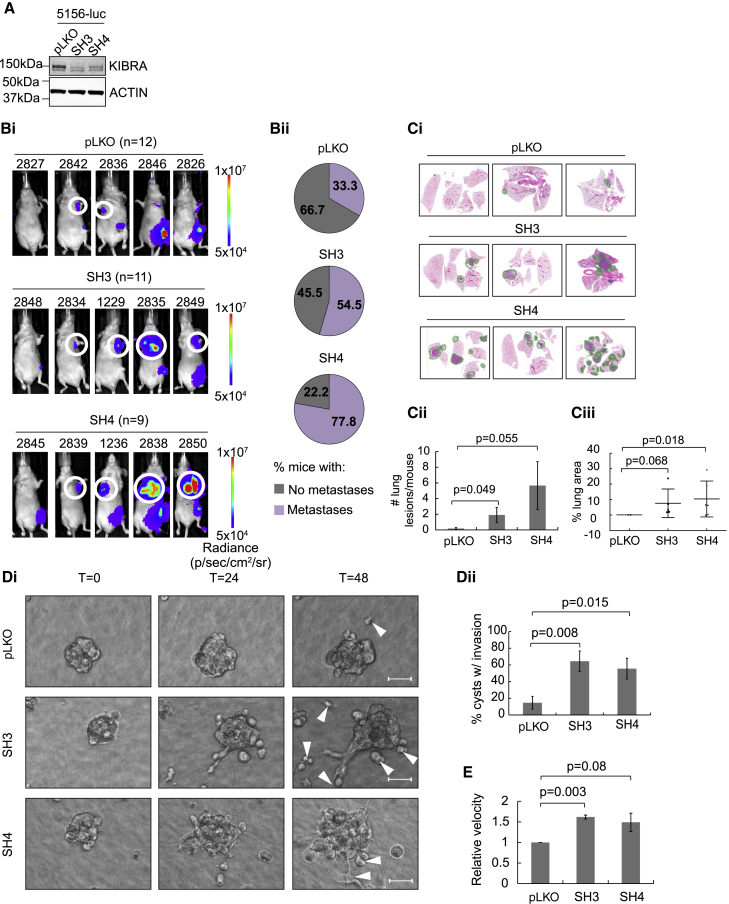
*Kibra* Silencing Increases Tumor Cell Aggressivity in Mice (A) Knockdown of *Kibra* in the MMTV-*Met* mammary tumor cell line 5156-luciferase (5156-luc). Two independent shRNAs (SH3 and SH4) are compared with a pLKO-empty vector control. (Bi) 5156-luc cells were orthotopically injected and resected after 5 weeks. Representative bioluminescence images of metastatic dissemination are shown. Metastases (white circles) were confirmed in histological sections. n = number of mice. (Bii) Percentages of mice with confirmed lung and lymph node metastases. (Ci) H&E-stained lung sections from 3 representative mice per condition. Metastatic lesions are outlined in green. (Cii) Quantification of lung metastatic burden. (Ciii) Calculation of the lung area containing tumor (mean ± SEM). (Di) Representative images of invasion (white arrows) from cysts into the collagen matrix. Scale bars represent 50 μm. (Dii) Quantification of invasion (3 independent experiments, means ± SEM). (E) Migration velocity of cells on fibronectin-coated plates (3 independent experiments, 30 cells/condition/experiment, mean ± SEM). See also Figure S3.

Low *KIBRA* expression in murine and human basal B cell lines was validated by real-time qPCR and western blotting (Figures S2C and S2D). *KIBRA* encodes a multi-domain scaffold protein (Kremerskothen et al., 2003) acting upstream of the Hippo tumor suppressor pathway, interacting with MERLIN and LATS1/2 to inhibit the oncogenic transcriptional co-activators YAP/TAZ (Baumgartner et al., 2010, Genevet et al., 2010, Yu et al., 2010). To understand the role of *KIBRA* loss, we silenced *Kibra* in cells from an MMTV-*Met* tumor, 5156, which retain chromosome 11 (Figure 2A). These cells were transduced with a luciferase-expressing lentivirus and orthotopically injected into nude mice. We observed no difference in primary tumor growth between control and *Kibra* knockdown cohorts (Figure S3). Because breast cancer morbidity and mortality are caused primarily by metastasis, and TNBC is highly metastatic, we resected primary mammary tumors and monitored mice for metastasis using bioluminescence imaging (Figure 2B). Compared with controls, tumors with *Kibra* silencing had an elevated capacity to metastasize to lungs and lymph nodes (Figures 2B and 2C). To determine whether this was due to increased invasion, we grew cells as 3D cyst-like structures and monitored their ability to invade a surrounding type I collagen matrix. *Kibra* knockdown significantly increased the percentage of cysts displaying invasion (Figure 2D). Accordingly, *Kibra* silencing also enhanced cell migration in two dimensions (Figure 2E). These data support a role for KIBRA in suppressing metastatic dissemination.

### *Kibra* Expression in Mouse Breast Cancer Cells Decreases Metastatic Potential

**Figure 3:**
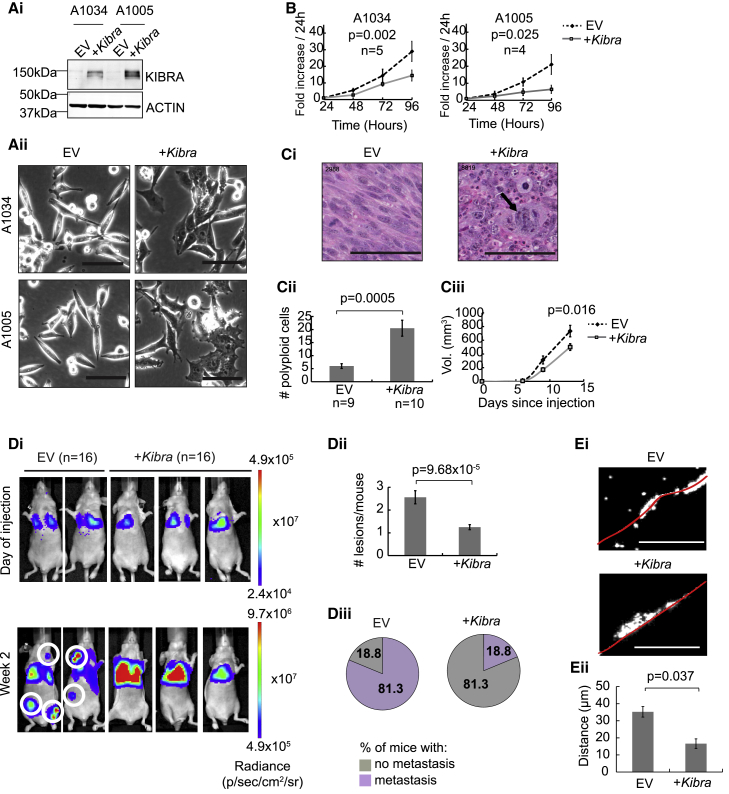
*Kibra* Re-expression Has an Anti-tumorigenic Effect (Ai) Western blot showing stable KIBRA re-expression in MMTV-*Met;Trp53fl/+;Cre* mammary tumor cells (A1034 and A1005). (Aii) Altered cell morphology in *Kibra*-expressing cells. EV, empty vector control. Scale bars, 100 μm. (B) Proliferation of cell lines with or without *Kibra*. Shown is the mean of the indicated replicates ± SEM. (Ci) H&E-stained mammary tumor sections from mice orthotopically injected with A1005 cells with or without *Kibra*. The arrow indicates an example of polyploidy. Scale bars, 100 μm. (Cii) Quantification of karyomegalic/multi-nucleated (polyploid) cells per section (mean ± SEM). (Ciii) Growth of tumors from (Ci) (mean ± SEM), showing significant difference in endpoint tumor size. (Di) Representative bioluminescent images of mice immediately after and 2 weeks after intravenous injection of A1005-luciferase cells with or without *Kibra.* n = number of mice. White circles highlight metastases outside of the lungs. (Dii) Number of metastatic sites per mouse (mean ± SEM). (Diii) Percentage of mice with metastatic sites outside of the lungs (mean ± SEM). (Ei) Representative images of invasion of DAPI-stained (white) A1005 cells with or without *Kibra*. Scale bars, 250 μm. (Eii) Quantification of invasion as distance traveled through collagen from the seeded area (red line in Ei) (n = 3, mean ± SEM). See also Figure S4.

To further understand the role of *KIBRA* loss in TNBCs, we overexpressed *Kibra* in MMTV-*Met;Trp53fl/+;Cre* tumor cells (A1005 and A1034) with spontaneous loss of chromosome 11 (Figure 3Ai). *Kibra* expression altered cell morphology (Figure 3Aii) and decreased proliferation *in vitro* (Figure 3B), and tumor cells grown orthotopically had altered pathology and decreased growth (Figure 3C). Interestingly, *Kibra*-positive tumors also displayed a significant increase in polyploidy (Figure 3C). This may be due to an increased rate of cytokinesis failure, providing an explanation for the reduced growth and smaller size of *Kibra*-positive tumors compared with controls (Figure 3C).

Because *KIBRA* knockdown in MCF10A cells induces EMT (epithelial-to-mesenchymal transition) (Moleirinho et al., 2013), we used real-time qPCR to determine whether *Kibra* expression modulates the expression of EMT regulators. Although *Kibra* expression significantly decreased the mRNA levels of *Twist2* (a transcriptional driver of EMT), it also increased the expression of its homolog *Twist1*, with no effect on other EMT drivers (Figure S4). Despite this, the mRNA levels of *E-cadherin* (*Cdh1*) and *Claudin-1* (*Cldn1*) were elevated upon *Kibra* expression, linking *Kibra* to an epithelial phenotype. These observations are reflected in the Cancer Genome Atlas (TCGA) dataset, where *KIBRA* and *CDH1* mRNA levels positively correlate in basal breast tumors, but no anti-correlation between *KIBRA* and EMT drivers is apparent (Figure S4).

Because *Kibra* depletion increased metastatic potential, we investigated the effect of *Kibra* re-expression on metastasis. Because spontaneous metastasis of A1005 cells from the mammary gland is variable, we injected them into the tail vein (Figure 3D). Strikingly, control cells disseminated extensively to sites outside of the lung 2 weeks post-injection. This was strongly suppressed by *Kibra* expression (Figure 3D), indicating that, although *Kibra*-positive cells survive and grow in the lung parenchyma, they are unable either to re-enter the bloodstream, survive in the circulation, or establish in sites other than the lungs, pre-requisites for further metastatic dissemination. Supporting an anti-metastatic function of *Kibra*, its expression decreased the invasion of a 3D collagen matrix by A1005 cells (Figure 3E). Together, these *in vivo* and *in vitro* assays demonstrate a metastasis-suppressive role for *Kibra*, consistent with its frequent loss in TNBCs.

### *KIBRA* Expression Inhibits Tumorsphere Formation in Human TNBC Cell Lines

**Figure 4:**
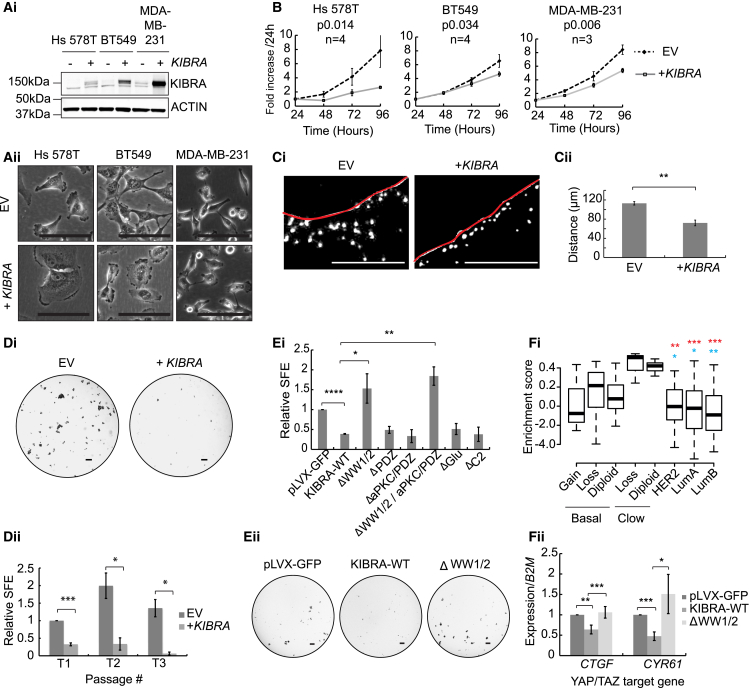
KIBRA Expression Reduces the Invasiveness and Tumorsphere-Forming Capacity of Breast Cancer Cells and Correlates with a YAP/TAZ Signature in Human Breast Cancers (Ai) Western blot showing stable KIBRA expression in 3 basal B breast cancer cell lines. (Aii) Images showing *KIBRA*-induced loss of mesenchymal features. Scale bars, 100 μm. (B) Proliferation with or without *KIBRA*. Shown are the mean values of the indicated replicates ± SEM. (Ci) Representative images of invasion of DAPI-stained (white) MDA-MB-231 cells with or without *KIBRA*. Scale bars, 250 μm. (Cii) Quantification of invasion as distance traveled through collagen from the seeded area (red line). n = 3, mean ± SEM. (Di) Representative images of MDA-MB-231 tumorspheres with or without *KIBRA*. Scale bars, 400 μm. (Dii) Sphere-forming efficiency (SFE) calculated at 3 serial passages (T1, T2, and T3) and normalized to EV control at T1 (n = 3, mean ± SEM). (Ei) SFE for MDA-MB-231 cells expressing a control (pLVX-GFP) compared with cells expressing either wild-type KIBRA (KIBRA-WT) or KIBRA mutants lacking specific regions as indicated (n = 3, mean ± SEM). (Eii) Representative images of tumorspheres in (Ei). Scale bars, 400 μm. (Fi) Gene set variation analysis (GSVA) showing enrichment of a *YAP/TAZ* gene expression signature. Basal and claudin-low subtypes are divided by *KIBRA* copy number gain or loss or diploid status. Asterisks indicate statistical differences between PAM50 subtypes and claudin-low (blue) or basal (red) tumors affected by *KIBRA* copy number loss. (Fii) qRT-PCR for YAP/TAZ targets in MDA-MB-231 cells expressing vector control, KIBRA-WT, or ΔWW1/2-KIBRA (n = 3, mean ± SEM). See also Figure S5.

To determine its effect on the biology of human TNBC, we re-expressed *KIBRA* in 3 TNBC cell lines (Figure 4Ai). As with murine TNBC, *KIBRA* expression altered the morphology, decreased proliferation, and decreased the ability to invade a collagen matrix (Figures 4A–4C). To examine how *KIBRA* influenced tumorigenic capacity, we grew cells under conditions of anoikis, as tumorspheres, to assay their tumor-initiating capacity and stem-like properties (Pece et al., 2010). *KIBRA* expression dramatically decreased tumorsphere propagation (Figure 4D; Figures S5A and S5B). Because sphere-forming efficiency (SFE) can indicate both tumorigenic and metastatic potential (i.e., the ability to seed, survive, and propagate at a secondary site), this is consistent with the role of *KIBRA* as a metastasis suppressor. Overall, these data show that *KIBRA* suppresses the tumorigenic and metastatic potential in TNBC cells and, therefore, that its loss can confer significant advantages to triple-negative tumors.

### Inhibition of Tumorsphere Formation by KIBRA Requires the WW1/2 Domains

To identify molecular mechanisms by which *KIBRA* functions as a tumor/metastasis suppressor, we systematically deleted regions of protein-protein interaction and structural regions and determined their role in tumorsphere formation (Figures S5C and S5D). MDA-MB-231 cells expressing wild-type KIBRA or mutants lacking the PSD95/DLG1/ZO-1 (PDZ)/atypical protein kinase C (aPKC) binding, Glu-rich, or C2 regions displayed reduced SFE compared with the empty vector control (Figure 4E). In contrast, KIBRA mutants lacking the WW1/2 domains did not impair tumorsphere formation, implicating proteins binding the KIBRA WW domains in the repression of tumorsphere formation.

**Figure 5:**
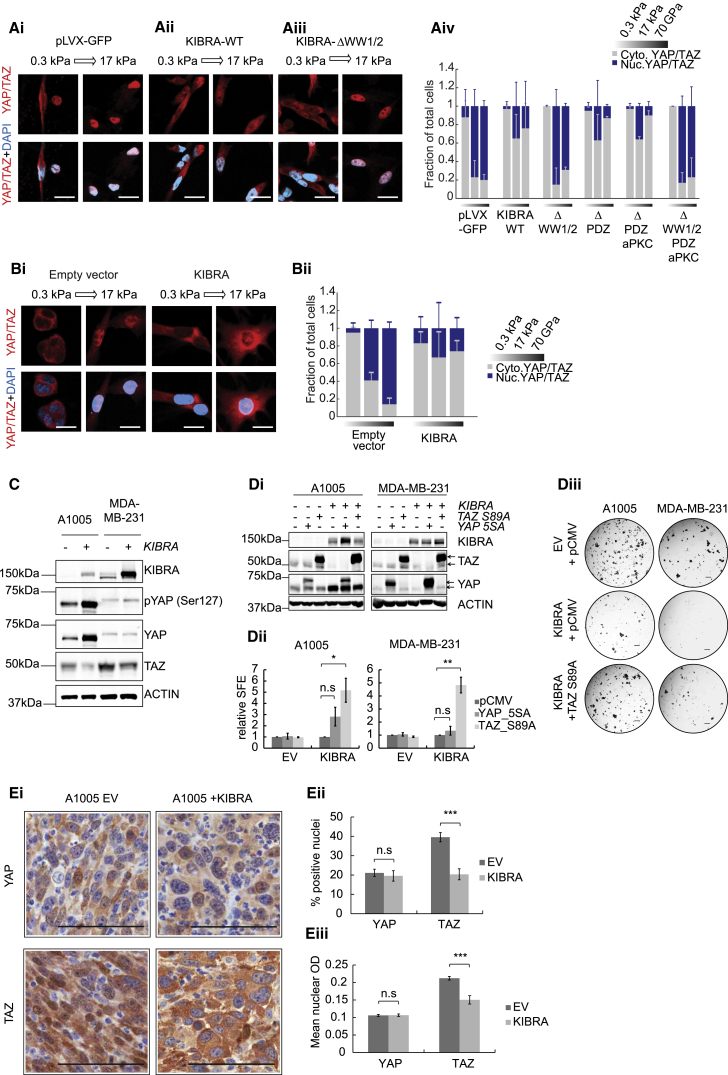
Anti-tumorigenic Effects of KIBRA Are Associated with a Reduction in TAZ Protein Levels and Inhibition of TAZ Nuclear Localization (Ai–Aiii) Subcellular localization of YAP/TAZ in MDA-MB-231 cells expressing wild-type KIBRA (KIBRA-WT), ΔWW1/2-KIBRA, or control (pLVX-GFP) in response to increasing matrix tension (0.3 to 17 kPa). Scale bars, 20 μm. (Aiv) Quantification of nuclear to cytoplasmic YAP/TAZ ratios under conditions of soft (0.3 kPa) or stiff (17 kPa) matrix or a collagen-coated glass coverslip (70 GPa) (n = 3, mean ± SD). (Bi and Bii) Representative images and quantification of YAP/TAZ localization in A1005 cells in response to matrix tension as in (A). (C) Western blot showing YAP phosphorylation and TAZ protein levels in MDA-MB-231 and A1005 cells with or without KIBRA. (Di) Western blots confirming transfection of constitutively active YAP or TAZ in A1005 and MDA-MB-231 cells with KIBRA. The empty pCMV vector is a negative control. The arrows indicate tagged (top) and endogenous (bottom) proteins. (Dii) Quantification of SFE relative to empty vector for cells in (Di) (n = 3, ± SEM). (Diii) Representative tumorsphere images. Scale bars, 400 μm. (Ei) Immunohistochemistry (IHC) showing YAP and TAZ subcellular localization in A1005 orthotopic tumors with or without *KIBRA*. Scale bars, 100 μm. (Eii and Eiii) Percentage of cells with positive nuclear staining (ii) and mean optical density (OD) of nuclear staining (iii) for YAP and TAZ in ten fields of view, 6 to 10 sections per condition (mean ± SEM). See also Figure S5.

Several studies have shown that increased TAZ activation endows mammary gland cells with stem-like properties (Bartucci et al., 2015, Cordenonsi et al., 2011). To examine the role of KIBRA in inhibiting YAP/TAZ, we initially examined the expression of a YAP/TAZ signature (Cordenonsi et al., 2011) in TCGA breast cancer data. Claudin-low and basal tumors with *KIBRA* copy number loss showed enrichment of this signature compared with those without *KIBRA* loss or other PAM50 subtypes (Figure 4Fi), suggesting that *KIBRA* loss increases YAP/TAZ activity in tumors with 5q deletion. Accordingly, *KIBRA* expression induced a significant WW domain-dependent decrease in mRNA levels of YAP/TAZ transcriptional targets *(CYR61* and *CTGF*) in MDA-MB-231 cells (Figure 4Fii). To examine this further, we assayed the effect of KIBRA on nuclear accumulation of YAP/TAZ using immunofluorescence. Using a stiffness-tenable polyacrylamide culture platform mimicking the mechanical rigidities of healthy and diseased breast tissue (Engler et al., 2006, Levental et al., 2009), we exploited the ability of YAP/TAZ to translocate to the nucleus in response to increasing extracellular matrix (ECM) stiffness (Dupont et al., 2011). Importantly, this allowed us to assay single cells, alleviating variability induced by changes in cell-cell contact. Compared with controls, KIBRA expression severely diminished nuclear YAP/TAZ localization in MDA-MB-231 and A1005 cells on stiff ECM (Figures 5A and 5B), an effect abrogated by deletion of the WW domains (Figure 5A). These data demonstrate that KIBRA prevents mechanotransduction-dependent nuclear accumulation of YAP/TAZ in a manner dependent on interaction(s) with its WW domains.

Although YAP and TAZ are generally considered to functionally overlap, it is *TAZ* specifically that is amplified in basal-like breast cancer and is associated with stem-like characteristics and metastatic potential (Chan et al., 2008, Cordenonsi et al., 2011, Skibinski et al., 2014). To determine the effect of KIBRA expression on YAP and TAZ, we used specific antibodies to examine their status in MDA-MB-231 and A1005 cells (Figure 5C). In agreement with previously published work (Xiao et al., 2011), we detected elevated YAP phosphorylation at Ser127 in cells expressing KIBRA, indicating inhibition. However, the increase in MDA-MB-231 cells was slight and, in A1005, correlated with increased YAP protein levels. More significantly, we observed a decrease in TAZ protein levels upon KIBRA expression in both cell lines (Figure 5C), which is consistent with the proteasomal degradation of TAZ that occurs following either Hippo pathway activation (Liu et al., 2010) or interference with mechanotransduction (Sorrentino et al., 2014).

To investigate the possibility that KIBRA functions through TAZ inhibition, we grew KIBRA-expressing cells as tumorspheres after transfection with constitutively active, serine-to-alanine mutants of YAP and TAZ (Figure 5Di), for which we confirmed nuclear localization (Figure S5E). Constitutively active TAZ, but not YAP, significantly increased the SFE of KIBRA-expressing cells (Figures 5Dii and 5Diii). In further support of a role for TAZ inhibition downstream of KIBRA, orthotopic A1005 tumors (Figure 3) showed prominent nuclear localization of TAZ that became cytoplasmic in tumors expressing KIBRA. YAP, however, remained largely cytoplasmic under all conditions (Figure 5E). Collectively, these data indicate that, in claudin-low breast cancer cells, loss of KIBRA promotes tumor progression and metastasis primarily by relieving inhibition of TAZ.

### KIBRA and PTPN14 Co-operate to Impair Breast Cancer Tumorsphere Formation

**Figure 6:**
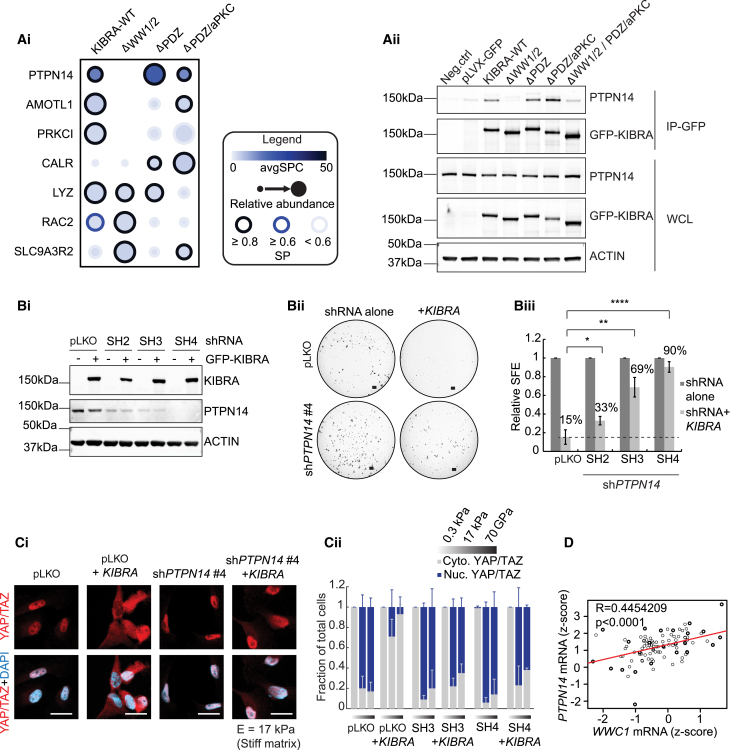
The KIBRA WW1/2 Domain Interactor PTPN14 Is Required for KIBRA-Mediated Inhibition of Tumorsphere Formation (Ai) High-confidence KIBRA-proximal proteins from BioID mass spectrometry analysis of MDA-MB-231 cells expressing WT or mutated KIBRA. (Aii) Co-immunoprecipitation of KIBRA and PTPN14 in MDA-MB-231 cells expressing wild-type or mutated KIBRA. (Bi) Western blot showing PTPN14 levels in MDA-MB-231-KIBRA cells expressing 3 *PTPN14* shRNAs (SH2, SH3, and SH4) or empty vector (pLKO). (Bii) Representative images of MDA-MB-231 tumorspheres expressing pLKO or *PTPN14* SH4 ± *KIBRA*. Scale bars, 400 μm. (Biii) SFE of MDA-MB-231 cells expressing pLKO or *PTPN14* shRNA with or without *KIBRA*, normalized to the appropriate shRNA-alone condition (conditions seeded in triplicate, mean of 2 experiments ± SEM). (Ci) YAP/TAZ localization in MDA-MB-231 cells expressing pLKO or *PTPN14* shRNA with or without *KIBRA*. Scale bars, 40 μm. (Cii) Quantification of YAP/TAZ nuclear to cytoplasmic ratios in MDA-MB-231 cells expressing pLKO or *PTPN14* shRNA with or without *KIBRA*, cultured on soft (0.3 kPa) or stiff (17 kPa) matrix or collagen-coated glass coverslips (70 GPa) (n = 3 mean ± SD). (D) Pearson correlation analysis of *WWC1* (*KIBRA*) and *PTPN14* mRNA levels (*Z* scores) in pooled basal and claudin-low patients (TCGA data, n = 89). See also Figure S6 and Table S4.

To clarify the WW domain interactions critical for KIBRA to suppress tumorsphere formation, we used BioID, a proximity-based strategy using biotinylation and mass spectrometry, for analysis of proximity-dependent interactions (Roux et al., 2012). Figure 6A, i, and Table S4 show high-confidence interactors (significance analysis of interactome [SAINT]express < 0.8) enriched in KIBRA BioID compared with negative controls in MDA-MB-231 cells. The only significant association lost by ΔWW1/2-KIBRA but retained in “non-rescue” mutants (lacking PDZ and aPKC binding domains), was with PTPN14 (protein tyrosine phosphatase non-receptor 14) (Poernbacher et al., 2012). We validated this interaction by co-immunoprecipitation (Figure 6Aii). Notably, the BioID failed to detect other known KIBRA interactors, including MERLIN and LATS1/2 (which were readily detected in other cell types such as HeLa; data not shown). Although MDA-MB-231 cells express LATS1, LATS2 is barely detectable, and MERLIN is not expressed (Figure S6A). Consistent with previous work (Xiao et al., 2011), KIBRA expression increased the levels of LATS1 and LATS2. However, KIBRA did not induce their auto-phosphorylation, indicating that KIBRA does not activate LATS1/2 in MDA-MB-231 cells (Figure S6A), possibly because of the absence of MERLIN (Baumgartner et al., 2010, Genevet et al., 2010, Yu et al., 2010). Interestingly, copy number loss of *KIBRA* can co-occur with that of *LATS1/2* or *NF2* (MERLIN), supporting LATS1/2 and MERLIN-independent functions of KIBRA in TNBCs (Cancer Genome Atlas Network, 2012; Figure S6B).

To investigate the role of the PTPN14-KIBRA interaction, we stably silenced *PTPN14* in MDA-MB-231 cells, expressed GFP-KIBRA, and seeded GFP-positive cells in tumorsphere assays (Figure 6Bi). KIBRA expression in control cells reduced SFE by 85%, which was rescued by *PTPN14* silencing in a manner correlative with the extent of knockdown (Figure 6B). This supports the hypothesis that PTPN14 co-operates with KIBRA to inhibit tumorsphere formation in MDA-MB-231 cells. To determine the role of the KIBRA/PTPN14 interaction in YAP/TAZ regulation, we evaluated YAP/TAZ subcellular localization in MDA-MB-231 cells, which express high levels of TAZ that decrease in response to KIBRA expression (Figure 5C). Strikingly, *PTPN14* silencing elicited a near-complete rescue of YAP/TAZ nuclear localization in KIBRA-expressing cells (Figure 6C), demonstrating co-operativity between KIBRA and PTPN14 in cytoplasmic sequestration of YAP/TAZ. This is supported by a highly significant correlation between *KIBRA* and *PTPN14* mRNA levels in basal and claudin-low tumors (Figure 6D).

### KIBRA and PTPN14 Promote YAP/TAZ Cytoplasmic Sequestration through Regulation of Actin Cytoskeletal Dynamics

**Figure 7:**
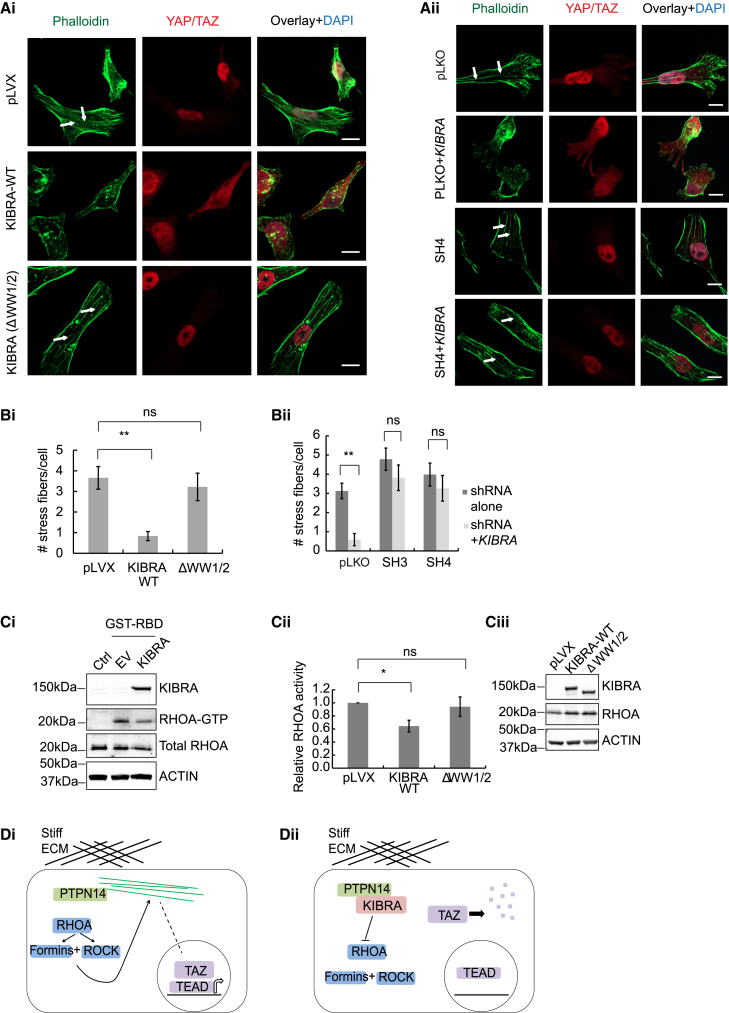
KIBRA and PTPN14 Co-operatively Regulate Actin Cytoskeletal Tension to Inhibit the Nuclear Translocation of YAP/TAZ (Ai) Representative phalloidin staining and YAP/TAZ immunofluorescence of MDA-MB-231 cells expressing empty vector, wild-type KIBRA, or ΔWW1/2 KIBRA seeded on collagen-coated coverslips. White arrows indicate actin stress fibers. Scale bars, 10 μm. (Aii) Representative immunofluorescence as in (Ai) for cells expressing pLKO control or sh*PTPN14* (SH4) with or without *KIBRA*. (Bi and Bii) Number of stress fibers per cell for (Ai) and (Aii) (n = 3, mean ± SEM). (Ci) Representative Rhotekin-GST pull-down in MDA-MB-231 cells expressing EV or KIBRA. GST alone was used as a control (Ctrl). (Cii) RHOA activity determined by G-LISAs (n = 3, mean ± SEM). (Ciii) RHOA protein levels for (Cii). (D) Schematic diagram showing regulation of TAZ by KIBRA. (Di) In the absence of KIBRA, stiff ECM activates RHOA, leading to actin stress fiber formation and contractility, facilitating TAZ nuclear translocation and interaction with TEA-domain (TEAD) transcription factors to promote expression of pro-oncogenic genes. (Dii) Association of KIBRA with PTPN14 inhibits RHOA activation required for actin stress fiber assembly, removing the stimulus for nuclear translocation of TAZ and resulting in its proteasomal degradation. See also Figure S7.

The regulation of YAP/TAZ localization by matrix tension or cell density involves modulation of the actin cytoskeleton (Aragona et al., 2013, Dupont et al., 2011). Consistent with this, expression of wild-type KIBRA, but not ΔWW1/2-KIBRA, decreased both actin stress fibers and nuclear localization of YAP/TAZ in MDA-MB-231 (Figures 7Ai and 7Bi) and A1005 cells (Figure S7) under stiff matrix conditions. These phenotypes were rescued by *PTPN14* silencing in wild-type KIBRA-expressing cells (Figures 7Aii, and 7Bii), demonstrating co-operativity between KIBRA and PTPN14 in regulating actin cytoskeletal dynamics to sequester YAP/TAZ in the cytoplasm. Furthermore, *Ptpn14* knockdown increased the metastasis of A1005 cells expressing *Kibra* to sites outside of the lungs (Figure S7), supporting the role of the KIBRA-PTPN14 interaction in suppressing metastasis *in vivo*.

The formation of actin stress fibers is controlled by RHOA, which activates formins that assemble F-actin and Rho-associated kinase (ROCK), which is required for stress fiber contractility (Narumiya et al., 2009). RHOA activation is therefore strongly implicated in YAP/TAZ nuclear localization caused by ECM stiffness (Dupont et al., 2011). We used Rhotekin-glutathione S-transferase (GST) pull-down assays (Ren et al., 1999) and an ELISA-based assay to detect guanosine triphosphate (GTP)-bound RHOA in cells expressing KIBRA (Figure 7C). Consistent with loss of stress fibers, KIBRA expression in MDA-MB-231 and A1005 cells decreased RHOA activity (Figure 7Ci; Figures S7Bi and S7Bii). This effect was not observed with ΔWW1/2 KIBRA (Figures 7Cii and 7Ciii), suggesting that the KIBRA-PTPN14 interaction represses RHOA activity to impair mechanotransduction-based regulation of TAZ, as shown schematically in Figure 7D.

## Discussion

The identification of syntenic regions of chromosomal loss in mouse cancer models and the human tumors they represent can aid in the identification of tumor suppressor genes (Liu et al., 2016, Xue et al., 2012). Here we have applied this strategy to show that mammary tumors from the MMTV-*Met;Trp53fl/+;Cre* mouse model lose a chromosomal region syntenic with human 5q33.2–35.3. Using a multifaceted approach, we identified *KIBRA* as a suppressor not only of tumor growth but also of metastasis. Selective pressure for loss of metastasis suppressor genes during tumorigenesis has been described (Al-Mulla et al., 2006, Cohn et al., 1991) and may reflect functional overlap between tumor initiation and aspects of the metastatic cascade. For example, the ability to survive and self-renew could contribute to dissemination and establishment at a secondary site. Notably, however, selective pressure for 5q loss might also be conferred by co-operative effects because of loss of multiple genes, including *KIBRA*. Indeed, it has been suggested that loss of multiple DNA damage response and cell cycle genes upon 5q deletion may promote genomic instability and tumor progression (Curtis et al., 2012, Weigman et al., 2012). This may explain why the re-introduction of KIBRA alone has a modest effect on tumor growth *in vivo*.

Diminished expression of KIBRA has been detected in claudin-low breast cancers, leukemia, and osteosarcomas (Basu-Roy et al., 2015, Hill et al., 2011, Moleirinho et al., 2013), although the underlying mechanisms have not been fully explored. Much of the premise for KIBRA as a tumor suppressor comes from its role in activating the Hippo pathway, for which loss of function and the concomitant activation of YAP/TAZ are well-documented in TNBCs (Cordenonsi et al., 2011). Hypermethylation of the LATS1 and LATS2 promoters is observed in 50% of breast cancers (Takahashi et al., 2005), whereas genomic loss of LATS1, LATS2, and NF2 also occurs in TNBC (Figure S6B). Amplification of TAZ occurs in ∼44% of basal breast cancers, where its expression confers stem-like and metastatic traits (Chan et al., 2008, Cordenonsi et al., 2011) and predicts poor outcome (Skibinski et al., 2014). Here, we provide evidence that hemizygous deletion of *KIBRA* increases TAZ activity in TNBC, with KIBRA expression inhibiting both tumorsphere formation (i.e., self-renewal of tumor-initiating cells) and the mechanosensing of a stiff ECM. The role of KIBRA in suppressing mechanical signals activating TAZ may be related to suppression of self-renewal, given that an undifferentiated stem-like state is maintained through contact with stiff ECM (Engler et al., 2006, Lui et al., 2012). Indeed, cells maintaining ECM contact in the basal layer of breast epithelium have nuclear TAZ, which becomes cytoplasmic as cells lose basement membrane contact and differentiate (Skibinski et al., 2014). KIBRA loss may constitutively activate mechanotransduction pathways that positively regulate TAZ, leading to persistent TAZ nuclear localization and maintenance of the poorly differentiated phenotype associated with basal-like tumors.

The mechanism of tumorsphere suppression by KIBRA involves its WW1/2 domain-mediated interaction with PTPN14. Although previous studies have shown that KIBRA and PTPN14 engage canonical Hippo signaling (Wilson et al., 2014), we found that they also co-operate to inhibit TAZ in MDA-MB-231 cells that lack MERLIN and activated LATS1/2 by inactivating RHOA and impairing actin stress fiber assembly. Although the metastasis suppressor phenotype conferred by KIBRA was only partially rescued by *Ptpn14* knockdown in A1005 cells, this may be due to residual inhibition of YAP/TAZ by canonical Hippo signaling, which, as we show, remains active in these cells. Hence, KIBRA inhibits YAP/TAZ via Hippo signaling or by activating the mechanotransduction-sensitive pathways that can promote YAP phosphorylation and TAZ degradation even in the absence of LATS1/2 and MERLIN (Sorrentino et al., 2014).

In addition to migration and invasion, cytoskeletal modulation by RHOA is critical for cytokinesis (Chircop, 2014). The accumulation of polyploid cells in A1005 KIBRA tumors may therefore involve decreased RHOA activity, which is known to cause growth arrest in tetraploid cells via activation of LATS2, subsequent YAP inhibition, and TP53 stabilization (Ganem et al., 2014). Although A1005 cells are *Trp53*-null, both LATS1/2 and YAP are phosphorylated upon KIBRA expression in A1005 cells (Figure S6), providing a partial mechanism by which KIBRA could impair growth.

Loss of heterozygosity (LOH) affecting large genomic regions occurs frequently in many cancers, including breast cancer (Solimini et al., 2012). The identification of genetic drivers for LOH and determination of their biological functions could provide new approaches for therapy. We demonstrate tumor-suppressive properties for the 5q gene *KIBRA*, which we link to tumor-initiating capacity and metastatic ability. We identify a Hippo pathway-independent function for KIBRA via its interaction with PTPN14, which itself has metastasis suppressor properties (Belle et al., 2015), in regulating YAP/TAZ localization through modulation of RHOA activity and the actin cytoskeleton. This contributes significantly to the understanding of cross-talk between actin cytoskeletal dynamics and YAP/TAZ function. The potential to target YAP/TAZ therapeutically, including through inhibiting mechanotransduction pathways, is currently being explored (Zanconato et al., 2016). Based on our findings, such therapeutic angles could be applied to TNBCs with 5q loss.

## Experimental Procedures

### Genomic Analyses

Genomic DNA and mRNA isolation and microarrays were performed as described previously (Knight et al., 2013). Patient gene expression and copy number information were obtained from a TCGA Breast Invasive Carcinoma dataset (Cancer Genome Atlas Network, 2012; http://cancergenome.nih.gov). Further details can be found in the Supplemental Experimental Procedures.

### Statistical Analyses

Statistical differences were calculated using Student’s t tests, where significance is as follows: p > 0.05, not significant (ns); ^∗^p ≤ 0.05; ^∗∗^p ≤ 0.01; ^∗∗∗^p ≤ 0.001; ^∗∗∗∗^p ≤ 0.0001. Statistical significance in Figures 1F and 4F was calculated by one-way ANOVA with *post hoc* Tukey’s multiple comparisons test. One luminal B patient-TCGA-E2-A155-01 was an outlier and was removed from all analyses.

### Cell Culture

Mouse tumor cells were isolated and cultured as described previously (Knight et al., 2013). All human cell lines were obtained from the ATCC and cultured in DMEM (Hs578T and MDA-MB-231) or RPMI medium (BT549) with 10% fetal bovine serum (FBS). *In vitro* assays are described in the Supplemental Experimental Procedures.

### Generation of Stable Cell Lines

The retroviral pBabe-*KIBRA* vector was a kind gift from Dr. Paul Reynolds (Addgene 40887). N-terminally GFP-tagged wild-type and mutant *KIBRA* were expressed from the pLVX lentiviral vector. Short hairpin RNAs (shRNAs) were expressed from pLKO.1 (Sigma-Aldrich). Further details can be found in the Supplemental Experimental Procedures.

### Transient Transfections

The vectors pCMV-FLAG YAP2 5SA (Kunliang Guan, Addgene 27371) and 3XFLAG pCMV-TOPO TAZ (S89A) (Jeff Wrana, Addgene 24815) were used. The empty pCMV vector was a negative control. Cells were transfected using Lipofectamine 3000 (Invitrogen) according to the manufacturer’s instructions. Further details can be found in the Supplemental Experimental Procedures.

### KIBRA Mutagenesis

*KIBRA* mutants were generated using Q5 site-directed mutagenesis (New England Biolabs) on a pENTR11-wild-type KIBRA vector, as detailed in the Supplemental Experimental Procedures.

### *In Vivo* Studies

All procedures involving mice were reviewed and approved by the McGill University Facility Animal Care Committee (FACC) and performed in accordance with university and national guidelines. Female 6-week-old friend virus B/NIH (FVB/N) mice were used for orthotopic mammary tumor growth experiments, and female 6-week-old NCr athymic nude mice (Taconic) were used for metastasis assays (tail vein injection and primary tumor resection assays). Bioluminescence imaging was performed weekly using the Xenogen IVIS 100 (Caliper LifeSciences) as described previously (Knight et al., 2013). Mammary tumor growth was monitored by twice-weekly caliper measurements. Further details can be found in the Supplemental Experimental Procedures.

### Microscopy

Phase contrast images were taken on an Axiovert 200M for adherent cells and an AxioScope Zoom for tumorspheres (both from Carl Zeiss). Immunofluorescence was imaged on an LSM800 confocal laser-scanning microscope (Carl Zeiss).

### YAP/TAZ Localization Assays

Polyacrylamide hydrogels, immunofluorescent staining, and analysis are described in the Supplemental Experimental Procedures. Each experiment was conducted in triplicate. An average of 45 cells was scored per replicate per condition.

### PCRs

Total RNA was isolated using the RNeasy mini kit (QIAGEN) and reverse-transcribed using the Transcriptor First Strand cDNA Synthesis Kit (Roche). Real-time PCR was performed as described previously, normalizing to *GAPDH* and *B2M* (human) or *Gapdh*, *Hprt*, and *Rpl13a* (mouse) (Knight et al., 2013). Primers (listed in the Supplemental Experimental Procedures) were designed using Primer3 (http://bioinfo.ut.ee/primer3-0.4.0/).

### RHO-A Activity Assays and Actin Stress Fiber Scoring

GST pull-downs and RHOA G-LISA assays (Cytoskeleton) are described in the Supplemental Experimental Procedures. Stress fibers were counted in ImageJ software, assessing a minimum of 12 cells per experiment in 3 experiments.

### BioID and Mass Spectrometry

*KIBRA* constructs were cloned into pSTV2-BirA^∗^-FLAG using Gateway LR clonase (Invitrogen). MDA-MB-231 expressing pSTV2-KIBRA constructs or vector alone were analyzed in biological duplicates. Expression and peptide isolation are described in the Supplemental Experimental Procedures.

## Data and Software Availability

The accession number for the raw and normalized aCGH and gene expression microarray data reported in this paper is GEO: GSE417748. The accession numbers for the mass spectrometry data reported in this paper are ProteomeXchange: PXD006608 and MassIVE: MSV000081111.

## Supplemental Information

Document S1. Supplemental Experimental Procedures, Figures S1–S7, and Tables S1 and S3

Table S2. Homologous Mouse and Human Genes Encoded within the Chr11–Chr5q Syntenic Genomic Region, Related to Figure 1

Table S4. BioID on KIBRA Constructs in MDA-MB-231 Cells Acquired on the Orbielite or LTQ Velos Mass Spectrometer, Related to Figure 6A The “Bait mutation' lists the gene name from NCBI followed by the mutation. “Prey Accession,” “Prey Gene Symbol” are from NCBI. “Spectra” are the individual spectral counts across both biological replicates (“|” serves as a delimiter), and the ‘Avg Spec’ across 2 biological replicates. The spectral counts across negative controls are used for scoring purposes, using both ‘AvgP’ and ‘MaxP’, and a simple Fold Change empirical calculation (see Supplemental Experimental Procedures for compression and scoring parameters). “BFDR” is the false discovery rate and the ‘Average spectra of unique peptides’ is the average number of unique peptides across 2 biological replicates, followed by the length of the prey sequence. The dataset is available for ftp download (see Supplemental Experimental Procedures).

Document S2. Article plus Supplemental Information
