# Supplementary material for: KIBRA (WWC1) Is a Metastasis Suppressor Gene Affected by Chromosome 5q Loss in Triple-Negative Breast Cancer
Source: Cell Rep. 2018 Mar 20;22(12):3191–205. doi: 10.1016/j.celrep.2018.02.095 (PMC5873529; doi:10.1016/j.celrep.2018.02.095)
Supplement: Document S1. Supplemental Experimental Procedures, Figures S1–S7, and Tables S1 and S3 [file mmc1.pdf]

Cell Reports, Volume *22*

Supplemental Information

KIBRA (WWC1) Is a Metastasis Suppressor Gene Affected by Chromosome 5q Loss in Triple-Negative Breast Cancer

Jennifer F. Knight, Vanessa Y.C. Sung, Elena Kuzmin, Amber L. Couzens, Danielle A. de Verteuil, Colin D.H. Ratcliffe, Paula P. Coelho, Radia M. Johnson, Payman Samavarchi-Tehrani, Tina Gruosso, Harvey W. Smith, Wontae Lee, Sadiq M. Saleh, Dongmei Zuo, Hong Zhao, Marie-Christine Guiot, Ryan R. Davis, Jeffrey P. Gregg, Christopher Moraes, Anne-Claude Gingras, and Morag Park

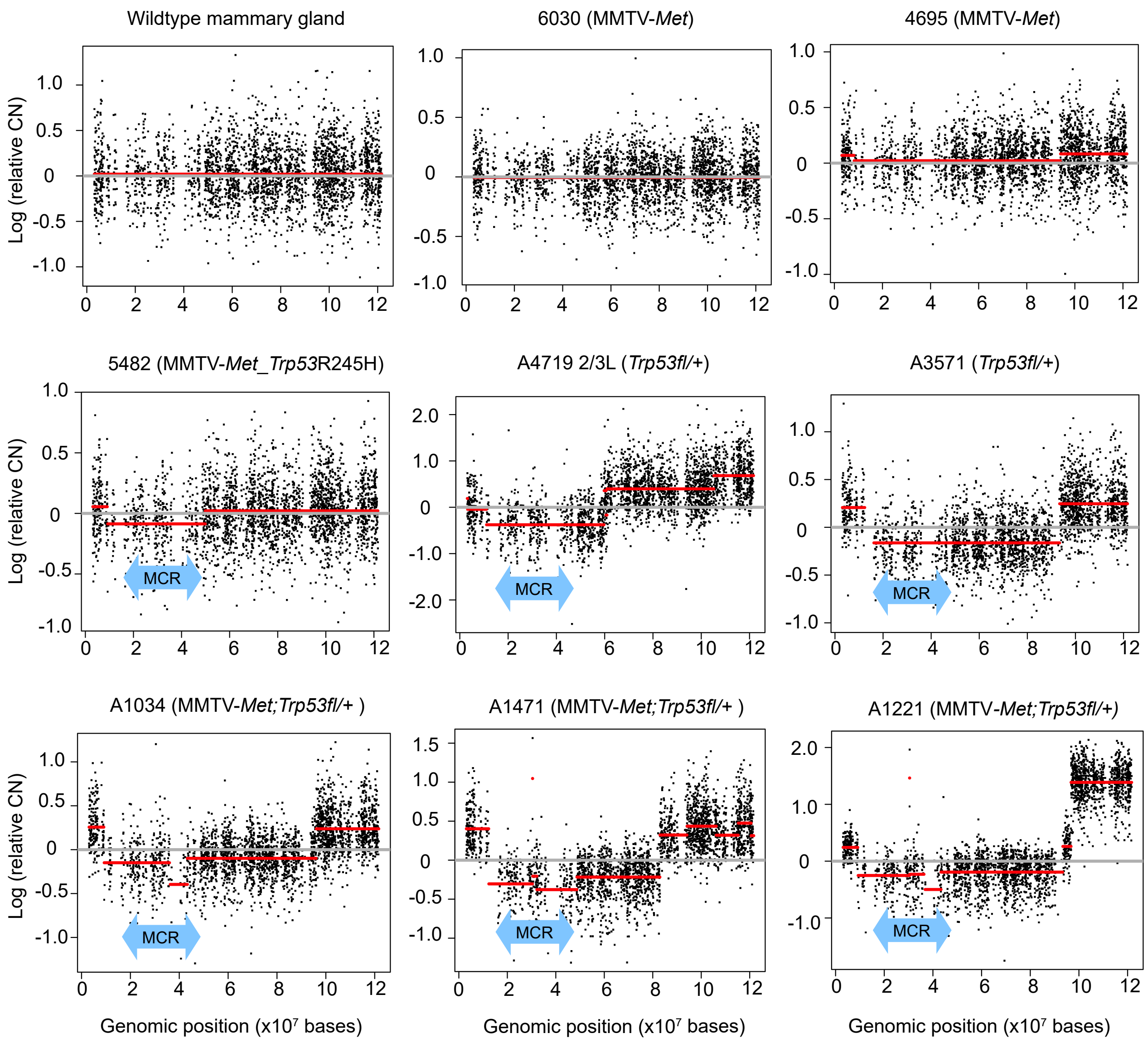

Supplemental Figure S1. Loss of chromosome 11 is a frequent event in MMTV-*Met;Trp53fl/+;Cre* and *Trp53fl/+;Cre* mouse mammary tumors. Refers to figure 1 of the main manuscript.

Examples of array-CGH (aCGH) profiles for mouse chromosome 11. Black dots indicate individual aCGH probes, red lines indicate segmented means for probe regions that deviate from a log copy number change of 0. A profile for chr11 in a normal wildtype mammary gland is shown, alongside profiles for 2 MMTV-*Met* model tumors (6030 and 4695), for which chr11 loss was an infrequent event (8/9 tumors showed no genomic loss). By contrast, loss of chr11 segments occurred frequently in tumors of the MMTV-*Met;Trp53fl/+;Cre* and *Trp53fl/+;Cre* models (18/19 tumors profiled), in addition to 1 MMTV-*Met* tumor with spontaneous *Trp53* mutation (5482). The region of chr11 loss common to all tumors with loss was defined (referred to as the 'minimal common region' or MCR) and is highlighted in blue. This region spans from position chr11:18862572 to 49845204bp.

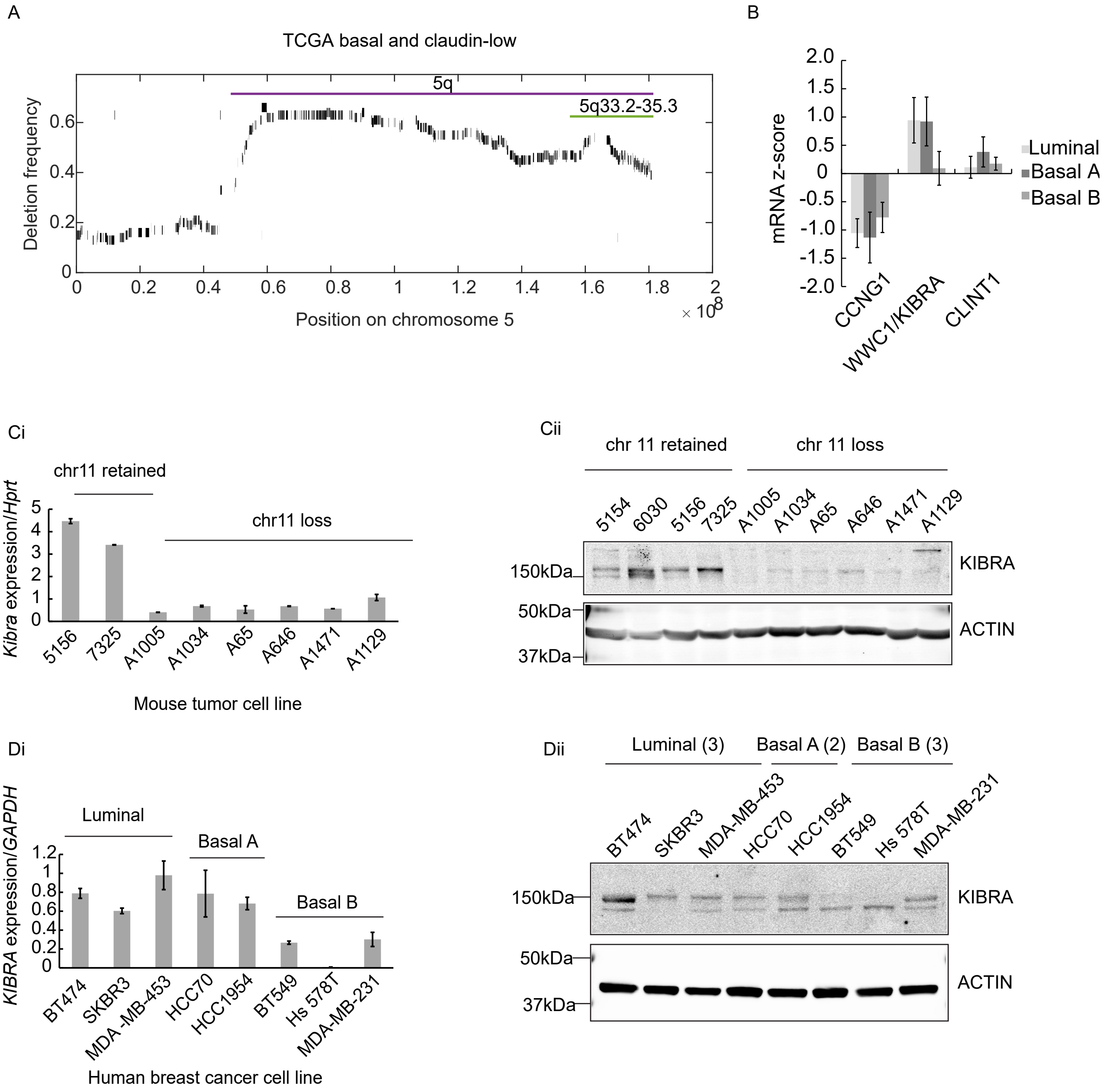

Supplemental Figure S2. Loss of chromosome 5q in basal and claudin-low breast cancers is associated with low expression of *KIBRA*. Relates to figure 1 of the main manuscript.

A) The Cancer Genome Atlas (TCGA) Invasive Breast Carcinoma single nucleotide polymorphism (SNP) array dataset, as analyzed by GISTIC, was used to investigate the frequency of gene loss on chromosome 5 among basal and claudin-low subtypes. Regions spanning the enitre long arm of chr5 (5q) occur in up to 60% of basal and claudin-low tumors. Region 5q33.2-35.3 is highlighted and represents the syntenic region of mouse chr11 that undergoes genomic loss in the transgenic breast cancer models used in this study. Loss of this region occurs in 40-55% of basal and claudin-low tumors. B) The Cancer Cell Line Encyclopedia (CCLE) was used to investigate mRNA expression of 3 genes (*CCNG1, KIBRA, CLINT1*) that undergo hemizygous deletion due to 5q loss (see main Figure 1). Human cell lines representative of the luminal, basal ('basal A') and claudin-low ('basal B') molecular subtypes were analysed. Low expression of *KIBRA* was specifically associated with basal B/claudin-low cell lines Ci) Quantitative real time PCR validated low *Kibra* mRNA expression in mouse mammary tumor cells with genomic loss of the syntenic region on mouse chr11. PCRs were performed in duplicate, error bars are SEM. Cii) Western blotting confirmed absence or low levels of KIBRA protein expression in mouse tumor cells affected by chr11 loss. Di) Quantitative real time PCR validated CCLE data showing reduced expression of *KIBRA* mRNA in basal B breast cancer cell lines compared to other subtypes. PCRs were performed in duplicate, error bars are SEM. Dii) Western blotting showed that KIBRA protein levels were low to absent in human cell lines belonging to the basal B/claudin-low subtype.

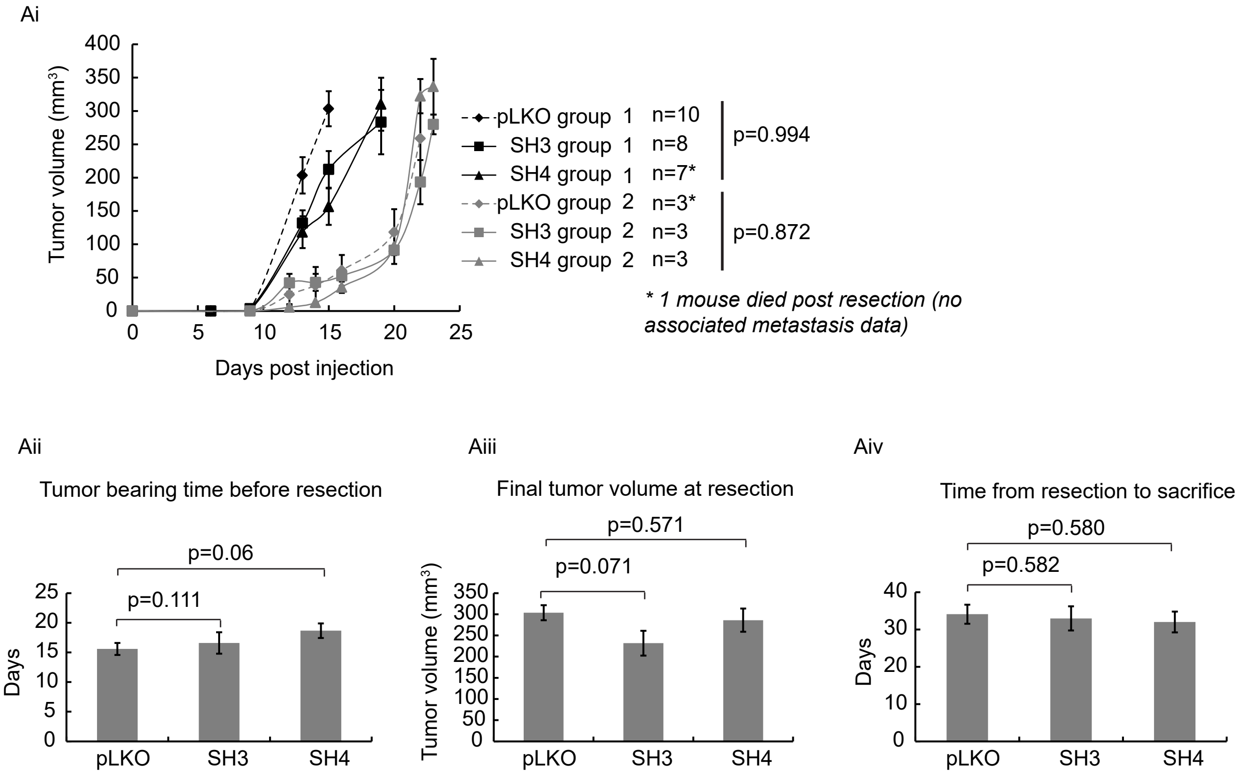

Supplemental Figure S3. Kibra knockdown has no significant impact on the *in vivo* growth of MMTV-*Met* driven mammary tumor cells. Related to Figure 2 of the main manuscript.

Ai) Primary tumour growth curves for mice presented in figure 2. Mammary fat pad injections were performed in nude mice using the MMTV-*Met* mouse mammary tumor cell line 5156-luc. Cells with *Kibra* knockdown (SH3, SH4) are compared to an empty vector control (pLKO). Two experimental groups containing the indicated number of mice are presented, mean values for all mice are shown, +/-SEM. Growth rates of pLKO and SH3/SH4 tumors were not statistically significant as determined by a Kruskal-Wallis One Way Analysis of Variance test. Aii-iv) Primary tumor resection data that accompanies results presented in Figure 2 of the main text. Tumor bearing time prior to resection, final tumor volume at resection and time from resection to sacrifice were equivalent between pLKO control and SH3, SH4 tumors. Mean values for all mice are shown, +/- SEM.

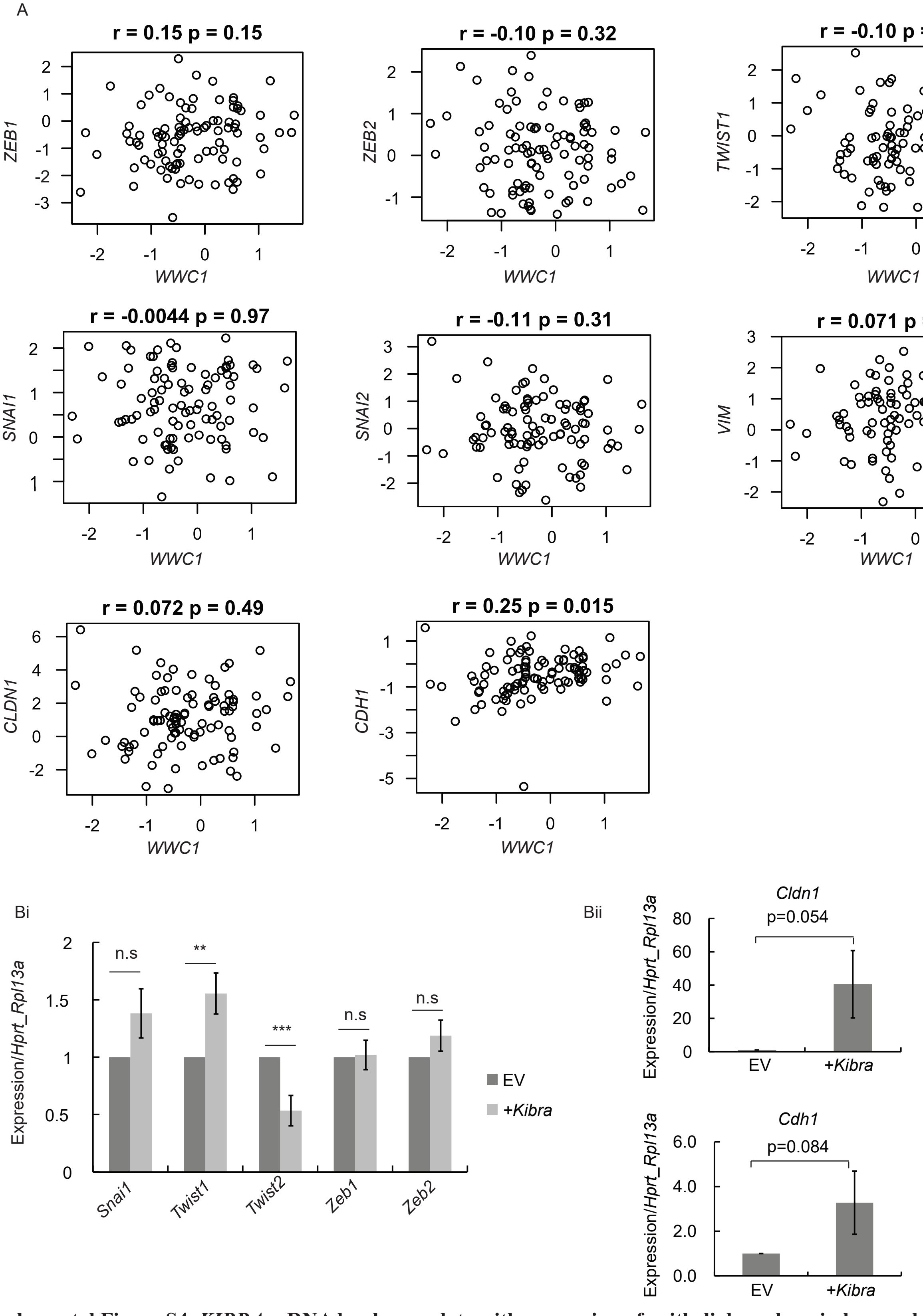

Supplemental Figure S4. *KIBRA* mRNA levels correlate with expression of epithelial markers in human breast cancers and mouse model tumor cells. Relates to figure 3 of the main manuscript.

A) Analysis of gene expression data for pooled basal and claudin-low tumors (TCGA, Nature 2012). Pearson correlation coefficients were calculated to determine the degree of correlation between mRNA levels of *WWC1* (*KIBRA*) and a panel of genes associated with either mesenchymal (*ZEB1/2, TWIST1, SNAI1/2, VIM*) or epithelial (*CLDN1, CDH1*) phenotypes. X and Y axis values are mRNA *Z*-scores. The only significant correlation was with *CDH1* (*E-CADHERIN*). B) RT-PCR data for mouse mammary tumor cells engineered to re-express *Kibra*. EV= empty vector control Bi) The only gene associated with a mesenchymal phenotype to significantly decrease following *Kibra* expression was *Twist2* (p=0.008). *Twist1* showed a compensatory increase (p=0.013). Bii) *Kibra* expression led to increases in the epithelial markers *Cldn1* (*Claudin 1*) and *Cdh1* (*E-Cadherin*). RT-PCR data were normalised to two housekeeping genes (*Hprt* and *Rpl13a*). The mean values for 3 independent experiments using two cell lines (A1005 and A1034) are shown. Error bars are SEM.

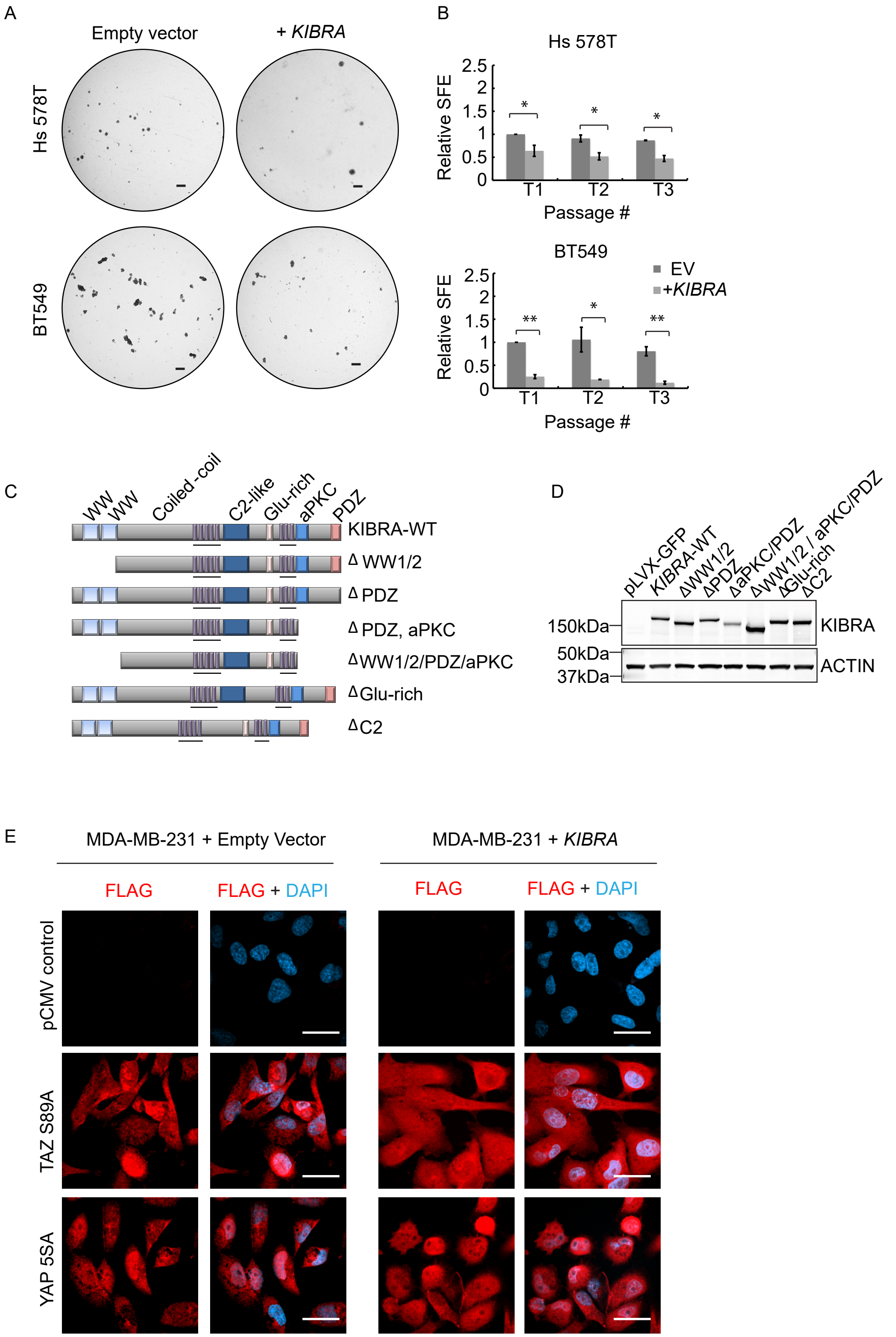

Supplemental Figure S5. *KIBRA* expression impairs tumorsphere formation in human basal B cell lines, a phenotype which requires the KIBRA WW-domains and can be rescued by expression of activated TAZ. Accompanies Figures 4 and 5 of the main manuscript.
A) Representative images of tumorspheres formed by basal B cell lines Hs 578T and BT549 +/- *KIBRA* expression. Scale bars are 400 µm. B) Quantification of sphere forming efficiency (SFE) for Hs 578T and BT549 cells +/- *KIBRA*. Results mirror those obtained with MDA-MB-231 cells as used in Figure 4 of the main manuscript (3 independent experiments, mean +/- SEM). C) Schematic showing GFP-tagged wildtype KIBRA (KIBRA-WT) and a series of KIBRA mutants lacking protein interaction and structural regions, including the WW-domains shown to be critical for the inhibition of tumorsphere formation (Figure 4) D) Western blotting showing expression of KIBRA-WT and KIBRA mutants in MDA-MB-231 cells (Figure 4). E) Immunofluorescent labelling of MDA-MB-231 cells +/- *KIBRA* and transfected with FLAG-tagged TAZ S89A or YAP 5SA mutants or a pCMV empty vector control (see Figure 5). Nuclear localisation of FLAG confirms TAZ and YAP activity in control and *KIBRA*-expressing MDA-MB-231. Scale bars are 20 µm.

A

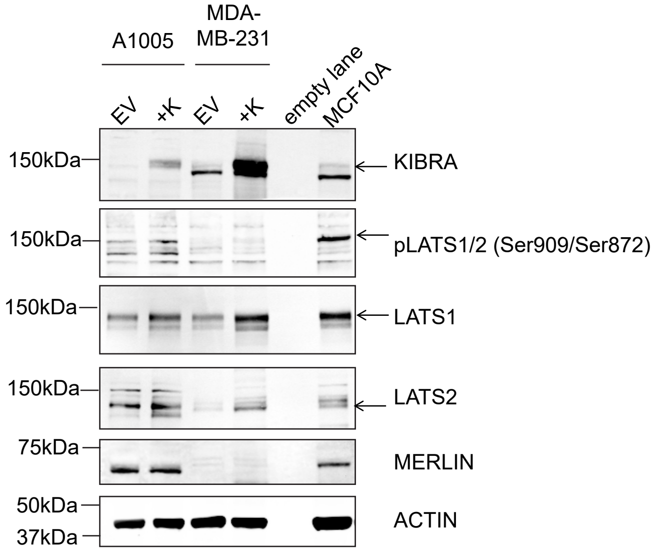

B

Gene (frequency of loss*)		p-value	Log odds ratio	Association
LATS2 (35%)	WWC1 (52%)	<0.001	1.8	Tendency towards co-occurrence
NF2 (17%)	WWC1 (52%)	0.005	2.0	Tendency towards co-occurrence
LATS1 (19%)	WWC1 (52%)	0.015	1.6	Tendency towards co-occurrence

** hetero- or homozygous loss in basal breast cancers (n=81)*

Supplemental Figure S6. MDA-MB-231 cells do not express MERLIN and do not show activation of LATS1/2. Relates to Figure 6 of the main manuscript.
A) Western blots showing expression of Hippo pathway components LATS1, LATS2 and MERLIN in cell lines A1005 and MDA-MB-231, +/- *KIBRA* expression (EV= empty vector control, +K = +*KIBRA*). The immortalised mammary epithelial line MCF10A is used as a positive control for Hippo pathway proteins and correct band sizes are highlighted by arrows. Whilst A1005 express both LATS1 and 2, expression of LATS2 is below the range of detectability in MDA-MB-231 EV cells. However, the presence of KIBRA leads to the stabilisation of LATS1 and 2 in both cell lines. Blotting for the auto-phosphorylation sites using an antibody that reacts with Serine 909 (LATS1) and Serine 872 (LATS2), gave no signal in MDA-MB-231 lysates +/- *KIBRA*, indicating lack of LATS1/2 activation in these cells. In addition, no signal was detected for MERLIN protein in MDA-MB-231. This is in contrast to A1005 cells, which express MERLIN and activate LATS1/2. B) CBioportal output showing the probability of there being co-occurrent deletion of *WWC1* (*KIBRA*) with other genes of the Hippo pathway (*LATS1*, *LATS2* and *NF2/MERLIN*). Analysis was restricted to the basal subtype. Dataset: Breast Invasive Carcinoma (TCGA, Nature 2012). Search terms entered: LATS1:HETLOSS HOMDEL LATS2: HETLOSS HOMDEL WWC1:HETLOSS HOMDEL NF2: HETLOSS HOMDEL. Small sample size (n=8 patients) for the claudin-low subtype precluded analysis of this subset.

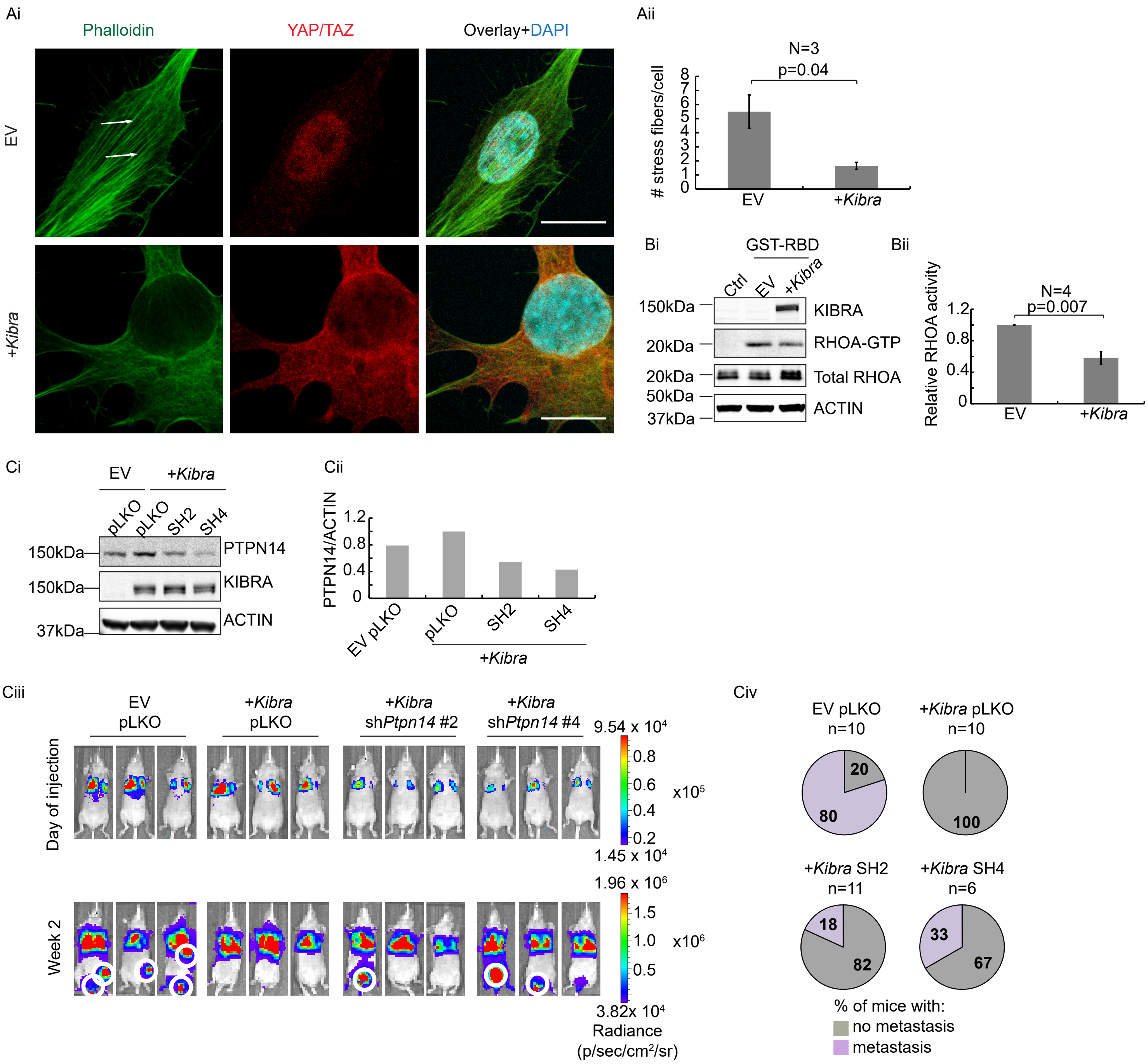

Supplemental Figure S7. ***Kibra*** **expression in A1005 cells disrupts ACTIN stress fibers and inhibits metastatic dissemination, which is partially restored by depletion of** ***Ptpn14*****. Relates to Figure 7 of the main manuscript.**

Ai) Subcellular localisation of YAP/TAZ in the mouse mammary tumor cell line A1005 +/- *Kibra* expression. EV=empty vector control. Cells were plated on glass coverslips overlayed with type I collagen to mimic a stiff-matrix environment. Under these conditions, EV cells show nuclear YAP/TAZ and abundant ACTIN stress fibers (white arrows). Expression of *Kibra* leads to accumulation of YAP/TAZ in the cytoplasm and stress fiber loss. Scale bars are 20 µm. Aii) Quantification of (Ai). The mean number of stress fibers per cell is shown. At least 12 cells per experiment were scored and data presented are the mean of 3 independent experiments. Error bars are SEM. Bi) Representative RHO-GST-pulldown using GST-fused to the Rho-binding domain of RHOTEKIN (GST-RBD). Pulldown was performed in A1005 cells +/- *Kibra*. GST-alone is a negative control (Ctrl). Bii) Quantification of RHO-GST pulldowns as shown in (Bi). RHOA activity is based on RHOA-GTP/Total RHOA band intensity. Data are the mean of 4 experiments, error bars are SEM. Ci) Knockdown of *Ptpn14* in A1005 cells +/- *Kibra*. Two independent shRNA oligos (SH2 and SH4) are used in addition to a negative control (pLKO). Cii) Quantification of PTPN14 protein levels shown in (Ci). Levels are normalised to cells expressing *Kibra* with pLKO negative control. Ciii) Representative bioluminescent images of mice injected intravenously with A1005 cells expressing EV or *Kibra* in combination with pLKO or *Ptpn14* shRNA as indicated. White circles indicate metastases outside of the lungs that were confirmed in histological sections. Civ) Percentages of mice with metastases outside of the lungs. Numbers of mice per group are indicated.

Table S1. A summary of published studies in which loss of chromosome 5q has been correlated with basal and triple negative breast cancers. Relates to figure 1 of the main manuscript.

		Clinical features associated with loss (% of patients affected)	
**Reference**	**Chr5q region lost (Cytoband)**	**Receptor status**	**Molecular subtype**
(Natrajan et al., 2009)	5q23.1-33.1		Basal (40-44%)
	5q33.2-33.3		
	5q33.3-35.3		
(Bergamaschi et al., 2006)	5q35.3	ER-negative	
	5q11-31		Basal
(Chin et al., 2006)	5q		Basal
(Horlings et al., 2010)	5q33.1	ER-negative	
(Sabatier et al., 2014)	5q		Basal and Claudin-low
(Turner et al., 2010)	5q23.1-34	Triple negative (71%)	
	5q34	Triple negative (43%)	
	5q34-35.3	Triple negative (66%)	
(Wang et al., 2004)	5q11.2-35.1	High grade, ER-neg/ HER2-neg, TP53 mutated	
(Johannsdottir et al., 2006)	5q (20 regions spanning 5q11.2-35.3)		*BRCA1* mutants (31-82%)
(Weigman et al., 2012)	5q11-35		Basal

Table S3. Mouse genes with decreased expression in mammary tumors that display chr11 loss, together with their unique human chr5q homologs. Relates to figure 1 of the main manuscript.

Mus musculus **gene**	**Chromosome location**	***Homo sapiens*** **homologue**	**Chromosome location**
Ranbp17	11:33211795-33513746	*RANBP17*	5:170861870-171300015
Gabrp	11:33550781-33578959	*GABRP*	5:170763350-170814047
Foxi1	11:34204338-34208089	*FOXI1*	5:170105897-170109725
Wwc1	11:35838400-35980527	*WWC1*	5:168291651-168472303
Ccng1	11:40748552-40755311	*CCNG1*	5:163437569-163446151
Ccnjl	11:43528784-43586997	*CCNJL*	5:160251652-160345396
Clint1	11:45852051-45910625	*CLINT1*	5:157785743-157859175
BC053393	11:46571536-46589232	*HAVCR1*	5:157029413-157059119
Timd2	11:46668960-46707061	*HAVCR1*	5:157029413-157059119
Havcr1	11:46735080-46779578	*HAVCR1*	5:157029413-157059119
Trim7	11:48826140-48852209	*TRIM7*	5:181193924-181205293
Olfr56	11:48978889-49135387	*OR2V2*	5:181154943-181155890
Olfr1393	11:49280150-49281085	*OR2Y1*	5:180739042-180740099

Thirteen mouse genes located on Chr11 31.4-49.8Mb that undergo hemizygous loss and have decreased gene expression in MMTV-*Met;Trp53fl/+;Cre* and *Trp53fl/+;Cre* mammary tumors. These are homologous with 11 unique human genes (listed) on chromosome 5q.

Experimental Procedures.

Genomic analysis of mouse model tumors. The aCGH data were analysed with the snapCGH version (1.42.0) Bioconductor R package (Smith ML, 2009). The cghMCR (1.30.0) Bioconductor R package was used to determine the minimal common region of loss (Zhang and Feng, 2016). Synteny between the MCR and human 5q was determined by analysis in Ensembl Genome Browser (Aken et al., 2016). Differentially expressed genes (Figure 1C) were identified using the limma Bioconductor package (3.28.21). Gene expression values were scaled by row with *Z*-score shown.

Genomic analysis of basal and claudin-low breast cancers. Patient data were obtained from TCGA (TCGA, 2012), using CBioportal (Gao et al., 2013) to generate the results shown in Figure 1 (D-F). Figure S2A was generated using copy number variations (CNVs) from the same dataset, as obtained from Firehose Broad GDAC (https://gdac.broadinstitute.org/; accessed on 31 July 2016). Frequencies of gene deletions residing on 5q as shown in Figure S2 were derived from the single nucleotide polymorphism array dataset (genome_wide_snp_6-segmented_scna_minus_germline_cnv_hg19) and analyzed by GISTIC2.0 (Mermel et al., 2011). Parameters used for analysis were: reference genome build hg19; amplification threshold 0.3; deletion threshold -0.3; join segment size 4; qv threshold 0.25; remove X chromosome yes; cap value 1.5; confidence level 95; broad analysis yes; broad length cut-off 0.5; maximum samples per segments per sample 2000; arm peel-off yes.

Generation of stable mouse and human cell lines. Selection of cells with stable expression of pBabe-*Kibra* was under Puromycin (2μg/ml). For A1034 and A1005 cells this included the drug efflux inhibitor Cyclosporin A (2.5 μM).

Short hairpin RNAs (shRNAs) targeting mouse *Kibra* in 5156-luc cells were expressed from a pLKO.1 vector (Dharmacon) and the following clones IDs were used: TRCN0000176876 (SH3) and TRCN0000177135 (SH4). Selection was under Puromycin (2μg/ml). Knockdown of *Ptpn14* in A1005 pBabe-EV and pBabe-*Kibra* cells was carried out with shRNAs cloned into 'pLKO.1-Blasticidin' and shRNAs had the following clone IDs: TRCN0000029015 (SH2) and TRCN0000029017 (SH4) (Sigma-Aldrich). Selection with Blasticidin (4μg/ml).

For *in vivo* experiments involving imaging of live mice, A1005 cells expressing pBabe-*Kibra* and 5156 cells with *Kibra* knockdown were transduced with pLenti PGK V5-Luc Neo lentivirus (Addgene 21471; Eric Campeau) and were selected under G418 (400μg/ml).

Expression of N-terminally GFP-tagged wildtype and mutant *KIBRA* in MDA-MB-231 cells was driven from the pLVX lentiviral vector. Cells underwent FACS for GFP-positivity 5 to 7 days post-infection. Sorted cells were cultured for 48 hours prior to use in functional assays.

Knockdown of *PTPN14* in MDA-MB-231 cells was carried out using pLKO.1 encoded shRNAs (Sigma-Aldrich) with the following clone IDs: TRCN0000006890 (SH2); TRCN0000006891 (SH3) and TRCN0000006892 (SH4). *PTPN14* knockdown cells were selected under Puromycin (2μg/ml) and were subsequently infected with pLVX-GFP-KIBRA lentivirus and sorted for GFP-positivity as described above.

Tumorsphere assays. Single cells were seeded in 6-well ultra-low attachments plates (Corning) in 2 ml serum-free DMEM/F12 supplemented with 1x B27, 10 μg/ml insulin (Gibco), 20 ng/ml EGF (BPS Bioscience), 20 ng/ml bFGF (StemRD), 10 μg/ml heparin (StemCell Technologies), and 0.5 μg/ml hydrocortisone (Wisent). 1% Methylcellulose (Sigma) was added to tumorsphere media to prevent cell clumping. BT549 and Hs 578T cells were seeded at 15,000 cells/well, A1005 at 10,000 cells/well and MDA-MB-231 cells at 7500 cells/well. To serially passage, tumorspheres were enzymatically and mechanically dissociated in 0.05% Trypsin-EDTA (Gibco), passed through a 25G needle, and re-seeded as single cells. For experiments involving constitutively active YAP and TAZ constructs, culture medium was changed 24 hours post-transfection. Cells were then trypsinised and resuspended for use in tumorsphere assays, seeding for protein isolation or staining with an anti-FLAG antibody a total of 48 hours post-transfection, as described below.

Proliferation assays. Cells were seeded in quadruplicate wells of a 12 well tissue culture plate (20,000-40,000 cells/well). Cells from one well were trypsinised and counted at 24hr intervals over 96hrs. Counts were performed using the Auto T4 Cellometer (Nexcelom Bioscience).

5156 Migration assays. Cells were plated at 2.5x10^4 cells per well of a 24-well plate pre-coated with 10ug/ml Fibronectin (EMD Millipore FC010). Cells were allowed to adhere for 4 hours (37 degrees, 5%CO_2) before being transferred to an Axiovert 200M inverted microscope equipped with a motorized stage and Climabox that was maintained at 37 degrees, 5% CO_2 (Carl Zeiss Inc.). AxioVision LE software (Carl Zeiss Inc.) was used to pre-programme positions for imaging. Images were captured every 10 minutes for 8 hours. Cell tracks were generated by manual tracking using MetaMorph software version 7.7.7.0 (Molecular Devices) and analysed using Microsoft Excel.

Geltrex-collagen invasion assays. Twenty-four well plates were pre-treated with 100% ethanol (10 mins) before being coated with Geltrex growth factor-reduced basement membrane matrix (25µl/well) (Life Technologies). Coated plates were incubated at 37°C (30 mins). The cell line 5156-luc was seeded at 5×10^4 cells/well in medium that contained Geltrex (2%) and cultured for 1 day to form cysts. Culture medium was then removed, before adding Type I Human Collagen Solution that had been prepared in PBS and 0.01N NaOH (3mg/ml; 400 µl/well) (Advanced BioMatrix). Plates were then incubated at 37°C (1 hour) before fresh cell culture medium was applied (1ml/well). The cysts were imaged every 15 mins for 48 hours using an Axiovert 200M microscope (Carl Zeiss Inc) as described for migration assays. Cysts with a diameter greater than 0.4 µm at the start were monitored for cell scattering as a measure of invasion into the collagen matrix.

Collagen invasion assays. Assays were performed as detailed elsewhere (Brekhman and Neufeld, 2009) with minor modifications as follows. Type I Human Collagen Solution (Advanced BioMatrix), prepared to 3mg/ml in PBS and 0.01N NaOH with no additional supplements, was used. Both cell lines were seeded in between the two layers of collagen at 5x10^4 / chamber of an 8-well chamber slide (Nunc). Cells were grown for 7 days prior to fixation. Fixed collagen gels were embedded in OCT and immediately frozen in liquid nitrogen. Constructs were flipped by 90-degrees and sectioned at 8µm thickness. Sections were mounted on microscope slides pre-coated with Poly-L-Lysine (Sigma, P8920) as described by the manufacturer. Sections were dried and nuclei stained with DAPI prior to mounting with a glass coverslip. Sections were imaged at 4X magnification using an EVOS Cell Imaging System. Invasion of individual cells into collagen was quantified using MetaMorph software (Molecular Devices). Briefly, a line was drawn to indicate the plane where cells were seeded and was used to generate an Euclidean distance map. Nuclei that had been identified by the 'granularity' application were transferred onto the Euclidean map to calculate the distance invaded into the collagen either side of the line. The mean distance of all nuclei was calculated. Three images were analysed per condition and 3 independent experiments were performed.

In vivo assays. Sh*Kibra* resection experiments were performed in 6-week old female Athymic nude mice (Taconic Farms, Inc.). Cells were injected into the 4th mammary gland (MFP4-Left; 1x10^6 cells/mouse) and mammary tumors surgically resected before reaching 500mm^3. Bioluminescent signal due to re-growth at or near to the primary resection site was excluded in scoring of metastatic burden. Lung metastases were scored in Haematoxylin and Eosin (H&E) stained sections as described (Knight et al., 2013).

A1005 cells expressing pBabe-empty vector or pBabe-*Kibra* were injected orthotopically into 6 week old female FVB/N mice bred in-house (MFP4-Left; 5x10^4 cells/mouse).

Intravenous (tail vein) injections of A1005-luciferase expressing cells were performed in 6 week old female Athymic nude mice (Taconic Farms, Inc.) as described previously (Knight et al., 2013), injecting 0.5x10^6 cells per mouse.

Tissue processing and Immunohistochemistry. Mouse tissue was fixed and processed as described (Knight et al., 2013). Haematoxylin and Eosin stained sections of mammary tumor were evaluated with the help of a pathologist (M.C.G) and were imaged using an Aperio-XT slide scanner (Aperio Technologies). The extent of polyploidy was evaluated by manually counting the number of cells exhibiting karyomegaly (enlarged nuclei) or the presence of multiple nuclei in one cell body. One section from each tumor (n=9 EV; n=10 +*Kibra*) was evaluated in Aperio ImageScope software and the entirety of each section was scored. Immunohistochemical staining was carried out using the Discovery Ultra Autostainer (Ventana Medical Systems Inc). Specific antibodies for YAP (Cell Signaling 14074) or TAZ (Atlas Antibodies HPA0077415) were used at 1/400 and 1/200 dilution, respectively. Stained tissue sections were imaged using an Aperio-XT slide scanner (Aperio Technologies). Staining was quantified using HALO 2.0 analysis software (Indica Labs) and the algorithm 'Cytonuclear'.

KIBRA mutagenesis. Mutagenesis was performed on a Gateway Entry vector encoding wildtype *KIBRA* (pENTR11-wildtype *KIBRA*) using the primers indicated below. *KIBRA* constructs were transferred by Gateway LR clonase recombination (Invitrogen) into the acceptor pLVX-GFP lentiviral vector. Constructs were transformed into Stbl3 cells at 30°C to avoid spontaneous recombination prior to DNA purification. The presence of the correct inserts was verified by restriction enzyme digestion with *Mun* I (Fermentas) and by Sanger sequencing.

Mutagenesis primers.

(ΔWW1/2): 5'-TGGCGGCGGGAGCAGGAA-3' and 5'-CATCCATGGGAAGCCTGCTTTTTTGTAC-3'

(ΔPDZ): 5'-TAAGCGGCCGCACTCGAG-3' and 5'-GAGAGCTGGGATATTCATCCGAG-3'

(ΔaPKC/PDZ): 5'-TAAGCGGCCGCACTCGAG-3' and 5'-GGAGCTGTCACTATCACTCCG-3'

(ΔGlu-rich): 5'-AAAGCCTCACCTGATATGG-3' and 5'-CAGTGTCTGTGTGCTGCTC-3'

(ΔC2): 5'-CTCAGCTACAAATACTTGAAG-3' and 5'-CGATTCGTCACTGTCAAATG-3'

PCR primers

Human *CTGF* Fwd: 5'-GCAGGCTAGAGAAGCAGAGC-3'

Human *CTGF* Rvs: 5'-TGGAGATTTTGGGAGTACGG-3'.

Human *CYR61* Fwd: 5'-GGAAAAGGCAGCTCACTGAA-3'.

Human *CYR61* Rvs: 5'-GAGCACTGGGACCATGAAGT-3'.

Human *B2M* Fwd: 5'-TGACTTTGTCACAGCCCAAG-3'.

Human *B2M* Rvs: 5'-AGCAAGCAAGCAGAATTTGG-3'.

GAPDH Fwd: 5'-CTGCACCACCAACTGCTTAG-3'.

GAPDH Rvs: 5'- GTCTTCTGGGTGGCAGTGAT-3'.

Human *KIBRA* Fwd: 5'-GCCTCACCTGATATGGATGG-3'.

Human *KIBRA* Rvs: 5'-CCACTCTCCGGTCCTTAGGT-3'.

Mouse *Kibra* Fwd: 5'-AAGATACCGGCTGGAGGAAC-3'.

Mouse *Kibra* Rvs: 5'- AGCGGACACACAGGCTACTT-3'.

Mouse *Hprt* Fwd: 5'-GCCCCAAAATGGTTAAGGTT-3'.

Mouse *Hprt* Rvs: 5'- CAAGGGCATATCCAACAACA-3'.

Mouse *Rpl13a* Fwd: 5'-AAGGCCAAGATGCACTATCG-3'

Mouse *Rpl13a* Rvs: 5'-GAGTCCGTTGGTCTTGAGGA-3'

Mouse *Zeb1* Fwd: 5'-TGAAGGTGATCCAGCCAAAC-3'

Mouse *Zeb1* Rvs: 5'-GGCGTGGAGTCAGAGTCATT-3'

Mouse *Zeb2* Fwd: 5'-TGGCCTATACCTACCCAACG-3'

Mouse *Zeb2* Rvs: 5'-GTGCTCCATCCAGCAAGTCT-3'

Mouse *Snai1* Fwd: 5'-CTTGTGTCTGCACGACCTGT-3'

Mouse *Snai1* Rvs: 5'-GCAGTGGGAGCAGGAGAAT-3'

Mouse *Twist1* Fwd: 5'-CTCGGACAAGCTGAGCAAG-3'

Mouse *Twist1* Rvs: 5'-CAGCTTGCCATCTTGGAGTC-3'

Mouse *Twist2* Fwd: 5'-ATGTCCGCCTCCCACTAGC-3'

Mouse *Twist2* Rvs: 5'-GTCATGAGGAGCCACAAGGT-3'

Mouse *Cdh1* Fwd: 5'-GACGCTGAGCATGTGAAGAA-3'

Mouse *Cdh1* Rvs: 5'-CAGGACCAGGAGAAGAGTGC-3'

Mouse *Cldn1* Fwd: 5'-ATTGGCATGAAGTGCATGAG-3'

Mouse *Cldn1* Rvs: 5'-CCACTAATGTCGCCAGACCT-3'

YAP/TAZ localisation assays

Tunable elastic modulus cell culture substrate fabrication: Polyacrylamide (PA) hydrogels were polymerized on 12-mm-diameter coverslips, using an adapted protocol (Tse and Engler, 2010). Acrylamide 3% and bisacrylamide 0.059% were used to generate E ~ 0.3 kPa PA gels. Acrylamide 7.5% and bisacrylamide 0.236% were used to generate E ~ 17 kPa PA gels. Sulfo-SANPAH (0.05 mg/ml, G-Biosciences BC38) was added to PA gels and activated by ultraviolet irradiation for 4 min. PA gels were washed with phosphate-buffered saline (PBS) and then functionalized with type I collagen (0.05 mg/ml, Corning, Bovine 354231) overnight at 4°C. Gels were rinsed with PBS and irradiated with UV for 45 min immediately before cell culture.

Elastic modulus measurements: The stiffness of polyacrylamide hydrogels was mechanically characterized by rheometric analysis, using a parallel plate shear rheometer (Anton-Paar). One-millimeter thick polyacrylamide hydrogels were fabricated between two 3-(trimethoxysilyl)propyl methacrylate (MPS) treated coverslips and adhesively fixed between the rheometer plates. The storage modulus (G) was measured at a 5% strain and at 10 Hz, which was verified to be within the linear elastic regime by a strain sweep. The elastic modulus E was estimated to be E=3G, assuming incompressible material properties.

YAP/TAZ immunofluorescence staining: 80,000 cells were plated on 0.3 kPa PA gels and 10,000 on 17 kPa PA gels and coverslips coated directly with type 1 collagen without PA gel (denoted as 70 GPa (Seal A, 2001)) to ensure similar cell density at final time point. Cells were cultured for 72 hrs and then fixed in 4% paraformaldehyde (20 min), permeabilized with 0.2% Triton X-100 (10 min), blocked with 2% BSA (30 min), and then incubated with YAP/TAZ primary antibody (1:200, Cell Signaling 8418) (1 hr). The primary antibody was visualized with a fluorescent secondary antibody conjugated to Alexa Fluor 647 raised in donkey (1:1000, Invitrogen A-31573) together with Alexa Fluor 488 phalloidin (1:200, Invitrogen A12379) (1 hr). Nuclei were counterstained with 0.25 ng/ml DAPI (5 min). All steps were performed at room temperature. Images were acquired on the LSM800 confocal laser scanning microscope (Carl Zeiss, GmbH), using a 20X objective.

Scoring of YAP/TAZ localisation: The MetaXpress Cell Scoring Application was used to calculate ratios of mean fluorescence intensity in the nuclear vs. cytoplasmic compartments of segmented cells (Lin et al., 2015). Thresholds of ratio that were used to establish nuclear and cytoplasmic classes were derived based on precision-recall (PR) analysis using a true positive (TP) set of objects representing distinct subcellular localizations based on MDA-MB-231 expressing pLVX-empty vector across different matrix conditions as scored by visual inspection (Vizeacoumar et al., 2010; Zanella et al., 2007). For example, nuclear TP objects were those for which manual labels were nuclear. Precision was calculated as the fraction of cells correctly classified as nuclear compared to all cells that fall above a

given threshold, i.e. TP/(TP+FP) and recall was calculated as fraction of cells correctly classified as nuclear compared to all cells that are labelled as nuclear, i.e. TP/(TP+FN). Classification of YAP/TAZ localization in KIBRA mutants was achieved with P = 0.91, R = 0.82 for nuclear localization and with P = 0.76 and R = 0.88 for cytoplasmic localization. Classification of YAP/TAZ localization in *PTPN14* knockdown conditions was achieved with P = 0.94, R = 0.75 for nuclear localization and with P = 0.71 and R = 0.92 for cytoplasmic localization.

Immunofluorescent staining for FLAG-tagged YAP2 5SA or TAZ S89A constructs . Cells shown in Figure S5E were cultured on collagen coated glass coverslips as described for YAP/TAZ localisation assays. Cells were stained with an anti-FLAG tag antibody at 1/50 (Cell Signaling 14793) using conditions described above for endogenous YAP/TAZ.

Protein isolation and Western blotting. Protein extraction was performed on ice using either 1% Triton buffer (50 mM Hepes at pH 7.5, 150 mM NaCl, 1.5 mM $MgCl_2$, 1 mM EGTA, 10% glycerol, 1% Triton X-100) or RIPA buffer (1M Tris-Cl (pH8.0), 5M NaCl, 10% NP40, 0.5% Na-desoxy-cholate, 10% SDS) containing the following inhibitors: 1 mM phenylmethylsulfonyl fluoride, 1 mM sodium vanadate, 1 mM sodium fluoride, 10 μg/mL aprotinin, and 10 μg/mL leupeptin). Western blotting was performed using NuPAGE Bis-Tris 4-12% gradient gels in MOPS-SDS running buffer (Invitrogen). The LI-COR Odyssey system was used for detection (see below).

Antibodies used in Western blotting. KIBRA primary antibody (Cell Signaling 8774) was used at 1/600, PTPN14 (Cell Signaling 13808) at 1/1000, PTPN14 (R&D Systems MAB4458) at 1/600, MERLIN (Abcam 88957) at 1/2000, YAP at 1/1000 (Cell Signaling 14074), TAZ at 1/1000 (Cell Signaling 4883), pYAP Ser127 at 1/1000 (Cell Signaling 4911), LATS1 at 1/1000 (Cell Signaling 9153), LATS2 at 1/1000 (Bethyl labs A300-479A), pLATS Ser909 at 1/1000 (Cell Signaling 9157), Beta-Actin (Sigma) 1/10,000, and anti-GFP (Invitrogen LifeSciences A6455) at 1/1000. Secondary antibodies were IRDye 800 CW anti-rabbit and IRDye 680RD anti-mouse and were used as described by the manufacturer (LI-COR).

Immunoprecipitation. Co-immunoprecipitation of GFP-KIBRA and PTPN14 was performed using 500ug of protein input. Lysates were pre-cleared using IgA sepharose beads (GE Healthcare) (45 min). Incubation with anti-GFP antibody (Life Technologies A6455 at 1/500) was performed overnight with rocking, followed by incubation with IgA sepharose beads (1 hr). Beads were washed 3 times in 1% Triton lysis buffer (as described above). All steps were performed a 4 degrees. Protein was eluted by boiling in lamelli buffer containing 1mM DTT.

GST-pulldowns. GST-Rhotekin Rho-binding domain (RBD) protein was expressed in the bacterial strain BL21 DE3 pLysS from the vector pGEX2T, using Isopropyl β-D-thiogalactoside (IPTG) (0.5mM) for induction. RBD protein was conjugated to Glutathione-sepharose beads (Ren et al., 1999). Protocols for lysis and pulldown are described elsewhere (Coleman et al., 2001). Pulldowns were performed in triplicate. Pulldowns and whole cell lysates were analysed by Western blotting using a rabbit anti-RHOA antibody (Santa Cruz).

RHOA G-LISAs. The G-LISA RHOA Activation Assay was performed according to the manufacturer's instructions (Cytoskeleton Inc.) in triplicate. Duplicate absorbance readings per sample were measured using a Varioskan (Thermo Electron Corporation) with SkanIt RE software. Equal levels of total RHOA protein were verified by Western blotting of duplicate lysates.

BioID and mass spectrometry.

Lentiviral delivery of BirA-FLAG-KIBRA and biotin labeling*: BioID experiments were performed by lentiviral transduction in MDA-MB-231 cells, using a BirA*-FLAG cloning vector, pSTV2 (Samavarchi-Tehrani, manuscript in preparation), using Gateway cloning. KIBRA coding sequences (wild type and mutations as described above) were subcloned to induce a N-terminal BirA*-FLAG fusion. HEK293T cells (American Type and Tissue Collection, ATCC, Manassas, VA, USA; Cat# CRL-3216) were used for virus production. Briefly, 3 μg of psPAX2 (a gift from Didier Trono, AddGene #12260), 2 μg of VSV-G packaging vectors (a gift from Bob Weinberg, AddGene #8454) and 3 μg of pSTV2 harboring KIBRA were transfected into HEK293T cells using the jetPRIME reagent as per manufacturer's recommendations (Polyplus-transfection SA, Illkirch-Graffenstaden, France). After 10 hrs, media was changed to virus production media. Virus production media consists of DMEM supplemented with 5% heat-inactivated Fetal Bovine Serum (Gibco, ThermoFisher Scientific, Waltham, MA, USA) and 50 U/ml Penicillin-Streptomycin solution (Corning, Manassas, VA, USA); Virus was harvested at 36 hrs post media change. For all

experiments, MDA-MB-231 cells [American Type and Tissue Collection, ATCC, Manassas, VA, USA; Cat# HTB-26™] in a 15 cm dish at approximately 35-40% density were infected with an amount of KIBRA virus optimized to yield 75-85% infection; to enable doxycycline induction of expression, cells were co-infected with a similarly expressed EF1a-rtTA lentivirus. Cells were then grown until ready for the BioID experiment. One 15 cm dish was used for each biological replicate. Biological duplicates were prepared for all experiments (alongside negative controls). Cells at 75% confluence in 15 cm plates were induced with 1 μg/ml doxycycline and treated with 40 μM biotin for 24 hrs. At the end of the induction and labeling phase, cells were washed and harvested in cold PBS and flash-frozen until time of sample processing.

Streptavidin affinity purification and mass spectrometry: The frozen cell pellets were resuspended in ice-cold RIPA buffer (50 mM Tris-HCl (pH 7.5), 150 mM NaCl, 1% NP-40, 1 mM EDTA, 1 mM EGTA, 0.1% SDS, Sigma protease inhibitors P8340 1:500, and 0.5% sodium deoxycholate), supplemented with 250 U benzonase. Pellets were further processed as described (Hesketh et al., 2017). Peptides were analyzed by nano-LCMS using a home-packed 0.75 μm x 10 cm C18 emitter tip (Reprosil-Pur 120 C18-AQ, 3 μm). A NanoLC-Ultra HPLC system (Eksigent) was coupled to an LTQ Orbitrap Velos or Elite (Thermo Fisher Scientific) and samples were analyzed in data-dependent acquisition mode. A 60,000 resolution MS scan was followed by 10 CID MS/MS ion trap scans on multiple charged precursor ions with a dynamic exclusion of 20 s. The LC gradient was delivered at 200 nl/min and consisted of a ramp of 2-35% acetonitrile (0.1% formic acid) over 90 min, 35-80% acetonitrile (0.1% formic acid) over 5 min, 80% acetonitrile (0.1% formic acid) for 5 min, and then 2% acetonitrile for 20 min. This data set consisting of 10 raw files and associated peak list and results files have been deposited in ProteomeXchange (PXD006608) through partner MassIVE (MSV000081111). Raw files were converted to mzXML and mgf files using ProteoWizard 3.0.4468 (Kessner et al., 2008) and analyzed using the iProphet pipeline (Shteynberg et al., 2011) implemented within ProHits (Liu et al., 2010) as follows. The database consisted of the human and adenovirus sequences in the RefSeq protein database (version 57) supplemented with "common contaminants" from the Max Planck Institute (http://141.61.102.106:8080/share.cgi?ssid=0f2gfuB) and the Global Proteome Machine (GPM; http://www.thegpm.org/crap/index.html). The search database consisted of forward and reverse sequences (labeled "gi|9999" or "DECOY"); in total, 72,226 entries were searched. Spectra were analyzed separately using Mascot (2.3.02; Matrix Science) and Comet [2012.01 rev.3 (Eng et al., 2013)] for trypsin specificity with up to two missed cleavages; deamidation (Asn or Gln) and oxidation (Met) as variable modifications; the mass tolerance of the precursor ion was set at +/-12 parts per million (ppm), the fragment ion tolerance at +/- 0.6 amu. The resulting Comet and Mascot results were individually processed by PeptideProphet (Keller et al., 2002) and combined into a final iProphet output using the Trans-Proteomic Pipeline (TPP; Linux version, v0.0 Development trunk rev 0, Build 201303061711). TPP options were as follows: general options were -p0.05 -x20 -d"gi|9999," iProphet options were –ipPRIME, and PeptideProphet options were –OpdP.

Data analysis and visualization: For analysis with SAINTexpress (Teo et al., 2014), only proteins with an iProphet protein probability of >0.95 were considered. Hits were also restricted to those detected with a minimum of 2 unique peptides. Since only two control purifications were included as part of this analysis, we supplemented these controls with controls from the Contaminant Repository for Affinity Purification (CRAPome; controls were selected to model endogenous biotinylation, i.e. no bait, and promiscuous biotinylation, i.e. FLAG-BirA* alone, in two additional cell lines, namely HEK293 and HeLa cells (Mellacheruvu et al., 2013) . These (CC532, CC533, CC537, CC538, CC540, CC5541, CC546, CC547 were used) and the MDA-MB-231 generated here were compressed to two controls, and SAINTexpress analysis was performed. Here, we considered as high-confidence those hits that passed a 0.8 SAINTexpress cutoff. Visualization of the interactions as dot plots was through prohits-viz.lunenfeld.ca (Knight et al., 2017); once a particular prey passes the SAINTexpress threshold for at least one bait, all the quantitative data across all baits are retrieved and displayed. On these dot plots, the color intensity maps to the averaged spectral counts across both replicates (capped at 50 spectral counts), while the size of the circles is proportional to the maximal spectral count value for the bait across all samples analyzed in parallel. The confidence score from SAINTexpress is mapped as the edge color.

Supplemental References.

Aken, B.L., Ayling, S., Barrell, D., Clarke, L., Curwen, V., Fairley, S., Fernandez Banet, J., Billis, K., Garcia Giron, C., Hourlier, T., *et al.* (2016). The Ensembl gene annotation system. Database : the journal of biological databases and curation *2016*.

Bergamaschi, A., Kim, Y.H., Wang, P., Sorlie, T., Hernandez-Boussard, T., Lonning, P.E., Tibshirani, R., Borresen-Dale, A.L., and Pollack, J.R. (2006). Distinct patterns of DNA copy number alteration are associated with different clinicopathological features and gene-expression subtypes of breast cancer. Genes, chromosomes & cancer *45*, 1033-1040.

Brekhman, V., and Neufeld, G. (2009). A novel asymmetric 3D in-vitro assay for the study of tumor cell invasion. BMC cancer *9*, 415.

Chin, K., DeVries, S., Fridlyand, J., Spellman, P.T., Roydasgupta, R., Kuo, W.L., Lapuk, A., Neve, R.M., Qian, Z., Ryder, T.*, et al.* (2006). Genomic and transcriptional aberrations linked to breast cancer pathophysiologies. Cancer cell *10*, 529-541.

Coleman, M.L., Sahai, E.A., Yeo, M., Bosch, M., Dewar, A., and Olson, M.F. (2001). Membrane blebbing during apoptosis results from caspase-mediated activation of ROCK I. Nature cell biology *3*, 339-345.

Eng, J.K., Jahan, T.A., and Hoopmann, M.R. (2013). Comet: an open-source MS/MS sequence database search tool. Proteomics *13*, 22-24.

Gao, J., Aksoy, B.A., Dogrusoz, U., Dresdner, G., Gross, B., Sumer, S.O., Sun, Y., Jacobsen, A., Sinha, R., Larsson, E.*, et al.* (2013). Integrative analysis of complex cancer genomics and clinical profiles using the cBioPortal. Science signaling *6*, pl1.

Hesketh, G.G., Youn, J.Y., Samavarchi-Tehrani, P., Raught, B., and Gingras, A.C. (2017). Parallel Exploration of Interaction Space by BioID and Affinity Purification Coupled to Mass Spectrometry. Methods Mol Biol *1550*, 115-136.

Horlings, H.M., Lai, C., Nuyten, D.S., Halfwerk, H., Kristel, P., van Beers, E., Joosse, S.A., Klijn, C., Nederlof, P.M., Reinders, M.J.*, et al.* (2010). Integration of DNA copy number alterations and prognostic gene expression signatures in breast cancer patients. Clinical cancer research : an official journal of the American Association for Cancer Research *16*, 651-663.

Johannsdottir, H.K., Jonsson, G., Johannesdottir, G., Agnarsson, B.A., Eerola, H., Arason, A., Heikkila, P., Egilsson, V., Olsson, H., Johannsson, O.T.*, et al.* (2006). Chromosome 5 imbalance mapping in breast tumors from BRCA1 and BRCA2 mutation carriers and sporadic breast tumors. International journal of cancer Journal international du cancer *119*, 1052-1060.

Keller, A., Nesvizhskii, A.I., Kolker, E., and Aebersold, R. (2002). Empirical statistical model to estimate the accuracy of peptide identifications made by MS/MS and database search. Analytical chemistry *74*, 5383-5392.

Kessner, D., Chambers, M., Burke, R., Agus, D., and Mallick, P. (2008). ProteoWizard: open source software for rapid proteomics tools development. Bioinformatics *24*, 2534-2536.

Knight, J.D.R., Choi, H., Gupta, G.D., Pelletier, L., Raught, B., Nesvizhskii, A.I., and Gingras, A.C. (2017). ProHits-viz: a suite of web tools for visualizing interaction proteomics data. Nature methods *14*, 645-646.

Knight, J.F., Lesurf, R., Zhao, H., Pinnaduwage, D., Davis, R.R., Saleh, S.M., Zuo, D., Naujokas, M.A., Chughtai, N., Herschkowitz, J.I.*, et al.* (2013). Met synergizes with p53 loss to induce mammary tumors that possess features of claudin-low breast cancer. Proceedings of the National Academy of Sciences of the United States of America *110*, E1301-1310.

Lin, C.H., Pelissier, F.A., Zhang, H., Lakins, J., Weaver, V.M., Park, C., and LaBarge, M.A. (2015). Microenvironment rigidity modulates responses to the HER2 receptor tyrosine kinase inhibitor lapatinib via YAP and TAZ transcription factors. Molecular biology of the cell *26*, 3946-3953.

Liu, G., Zhang, J., Larsen, B., Stark, C., Breitkreutz, A., Lin, Z.Y., Breitkreutz, B.J., Ding, Y., Colwill, K., Pasculescu, A.*, et al.* (2010). ProHits: integrated software for mass spectrometry-based interaction proteomics. Nature biotechnology *28*, 1015-1017.

Mellacheruvu, D., Wright, Z., Couzens, A.L., Lambert, J.P., St-Denis, N.A., Li, T., Miteva, Y.V., Hauri, S., Sardiu, M.E., Low, T.Y.*, et al.* (2013). The CRAPome: a contaminant repository for affinity purification-mass spectrometry data. Nature methods *10*, 730-736.

Mermel, C.H., Schumacher, S.E., Hill, B., Meyerson, M.L., Beroukhim, R., and Getz, G. (2011). GISTIC2.0 facilitates sensitive and confident localization of the targets of focal somatic copy-number alteration in human cancers. Genome biology *12*, R41.

Natrajan, R., Lambros, M.B., Rodriguez-Pinilla, S.M., Moreno-Bueno, G., Tan, D.S., Marchio, C., Vatcheva, R., Rayter, S., Mahler-Araujo, B., Fulford, L.G.*, et al.* (2009). Tiling path genomic profiling of grade 3 invasive ductal breast cancers. Clinical cancer research : an official journal of the American Association for Cancer Research *15*, 2711-2722.

Ren, X.D., Kiosses, W.B., and Schwartz, M.A. (1999). Regulation of the small GTP-binding protein Rho by cell adhesion and the cytoskeleton. The EMBO journal *18*, 578-585.

Sabatier, R., Finetti, P., Guille, A., Adelaide, J., Chaffanet, M., Viens, P., Birnbaum, D., and Bertucci, F. (2014). Claudin-low breast cancers: clinical, pathological, molecular and prognostic characterization. Molecular cancer *13*, 228.
Seal A, D.A., Banerjee M, Mukhopadhyay AK, Phani KK (2001). Mechanical properties of very thin cover slip glass disc. Bulletin of Materials Science *24*, 151-155.
Shteynberg, D., Deutsch, E.W., Lam, H., Eng, J.K., Sun, Z., Tasman, N., Mendoza, L., Moritz, R.L., Aebersold, R., and Nesvizhskii, A.I. (2011). iProphet: multi-level integrative analysis of shotgun proteomic data improves peptide and protein identification rates and error estimates. Molecular & cellular proteomics : MCP *10*, M111 007690.
Smith ML, M.J., McKinney S, Hardcastle T and Thorne NP (2009). snapCGH: Segmentation, normalisation and processing of aCGH data. R-package version 1.46.0.
TCGA (2012). Comprehensive molecular portraits of human breast tumours. Nature *490*, 61-70.
Teo, G., Liu, G., Zhang, J., Nesvizhskii, A.I., Gingras, A.C., and Choi, H. (2014). SAINTexpress: improvements and additional features in Significance Analysis of INTeractome software. Journal of proteomics *100*, 37-43.
Tse, J.R., and Engler, A.J. (2010). Preparation of hydrogel substrates with tunable mechanical properties. Current protocols in cell biology *Chapter 10*, Unit 10 16.
Turner, N., Lambros, M.B., Horlings, H.M., Pearson, A., Sharpe, R., Natrajan, R., Geyer, F.C., van Kouwenhove, M., Kreike, B., Mackay, A., *et al.* (2010). Integrative molecular profiling of triple negative breast cancers identifies amplicon drivers and potential therapeutic targets. Oncogene *29*, 2013-2023.
Vizeacoumar, F.J., van Dyk, N., F, S.V., Cheung, V., Li, J., Sydorskyy, Y., Case, N., Li, Z., Datti, A., Nislow, C., *et al.* (2010). Integrating high-throughput genetic interaction mapping and high-content screening to explore yeast spindle morphogenesis. The Journal of cell biology *188*, 69-81.
Wang, Z.C., Lin, M., Wei, L.J., Li, C., Miron, A., Lodeiro, G., Harris, L., Ramaswamy, S., Tanenbaum, D.M., Meyerson, M., *et al.* (2004). Loss of heterozygosity and its correlation with expression profiles in subclasses of invasive breast cancers. Cancer research *64*, 64-71.
Weigman, V.J., Chao, H.H., Shabalin, A.A., He, X., Parker, J.S., Nordgard, S.H., Grushko, T., Huo, D., Nwachukwu, C., Nobel, A., *et al.* (2012). Basal-like Breast cancer DNA copy number losses identify genes involved in genomic instability, response to therapy, and patient survival. Breast cancer research and treatment *133*, 865-880.
Zanella, F., Rosado, A., Blanco, F., Henderson, B.R., Carnero, A., and Link, W. (2007). An HTS approach to screen for antagonists of the nuclear export machinery using high content cell-based assays. Assay and drug development technologies *5*, 333-341.
Zhang, J., and Feng, B. (2016). cghMCR: Find chromosome regions showing common gains/losses.
