# Supplementary material for: KIBRA (WWC1) Is a Metastasis Suppressor Gene Affected by Chromosome 5q Loss in Triple-Negative Breast Cancer
Source: Cell Rep. 2018 Mar 20;22(12):3191–205. doi: 10.1016/j.celrep.2018.02.095 (PMC5873529; doi:10.1016/j.celrep.2018.02.095)
Supplement: Document S2. Article plus Supplemental Information [file mmc4.pdf]

Cell Reports

Article

KIBRA (WWC1) Is a Metastasis Suppressor Gene Affected by Chromosome 5q Loss in Triple-Negative Breast Cancer

Graphical Abstract

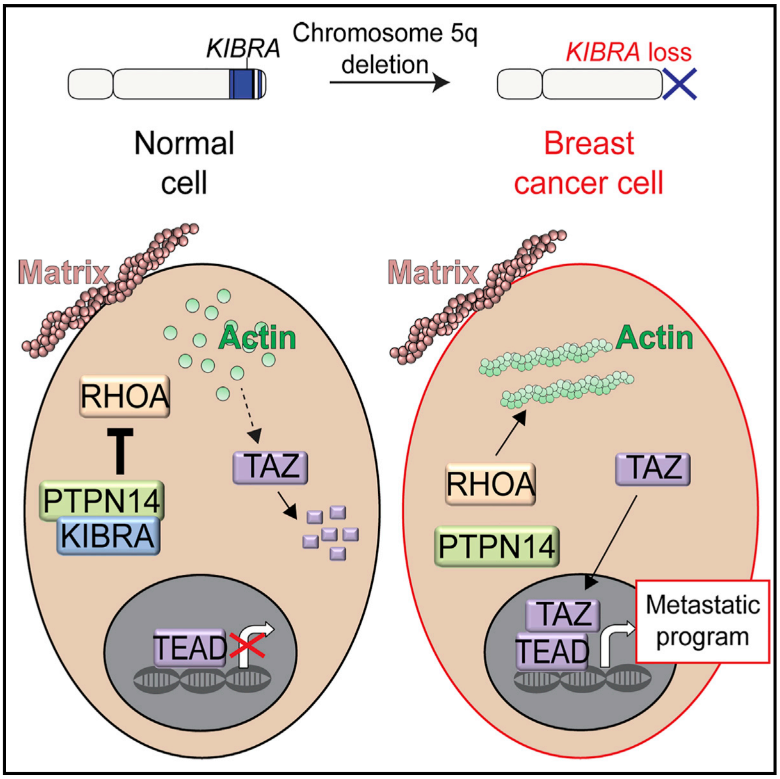

Highlights

- Reduced *KIBRA* expression is associated with chr 5q loss in breast cancer
- Restoring *Kibra* expression inhibits metastatic dissemination in mice
- KIBRA impairs the self-renewal capacity of triple-negative breast cancer cells
- KIBRA blocks mechanotransduction signals required for YAP/TAZ activation

Authors

Jennifer F. Knight, Vanessa Y.C. Sung, Elena Kuzmin, ..., Christopher Moraes, Anne-Claude Gingras, Morag Park

Correspondence

morag.park@mcgill.ca

In Brief

Triple-negative breast cancers (TNBCs) frequently lose chromosome 5q. Using a TNBC mouse model with spontaneous loss of a syntenic region, Knight et al. identify *KIBRA* as a metastasis suppressor. Mechanistically, KIBRA suppresses RHOA activation, impairing nuclear translocation of the oncogenes *YAP/TAZ*, which drive metastatic and cancer stem cell-like behavior.

Data and Software Availability

GSE417748
PXD006608

Knight et al., 2018, Cell Reports *22*, 3191–3205
March 20, 2018 © 2018 The Authors.
https://doi.org/10.1016/j.celrep.2018.02.095

CellPress

Cell Reports
Article

OPEN ACCESS
CellPress

KIBRA (WWC1) Is a Metastasis Suppressor Gene Affected by Chromosome 5q Loss in Triple-Negative Breast Cancer

Jennifer F. Knight,[1] Vanessa Y.C. Sung,[1,2] Elena Kuzmin,[1,2] Amber L. Couzens,[3] Danielle A. de Verteuil,[1] Colin D.H. Ratcliffe,[1,2] Paula P. Coelho,[1,2] Radia M. Johnson,[1] Payman Samavarchi-Tehrani,[3] Tina Gruosso,[1,4] Harvey W. Smith,[1] Wontae Lee,[5] Sadiq M. Saleh,[1] Dongmei Zuo,[1] Hong Zhao,[1] Marie-Christine Guiot,[6] Ryan R. Davis,[7] Jeffrey P. Gregg,[7] Christopher Moraes,[1,5,8] Anne-Claude Gingras,[3,9] and Morag Park[1,2,4,10,*]
[1]Goodman Cancer Research Centre, McGill University, Montreal, QC H3G 0B1, Canada
[2]Department of Biochemistry, McGill University, Montreal, QC H2W 1S6, Canada
[3]Lunenfeld-Tanenbaum Research Institute, Mount Sinai Hospital, Toronto, ON M5G 1X5, Canada
[4]Department of Oncology, McGill University, Montreal, QC H2W 1S6, Canada
[5]Department of Biomedical Engineering, McGill University, Montreal, QC H3A 2B4, Canada
[6]Montreal Neurological Institute, Department of Pathology, McGill University, Montreal, QC H3A 2B4, Canada
[7]Department of Pathology and Laboratory Medicine, University of California at Davis School of Medicine, Sacramento, CA 95817, USA
[8]Department of Chemical Engineering, McGill University, Montreal, QC H3A 0C5, Canada
[9]Department of Molecular Genetics, University of Toronto, Toronto, ON M5S 1A8, Canada
[10]Lead Contact
*Correspondence: morag.park@mcgill.ca
https://doi.org/10.1016/j.celrep.2018.02.095

SUMMARY

Triple-negative breast cancers (TNBCs) display a complex spectrum of mutations and chromosomal aberrations. Chromosome 5q (5q) loss is detected in up to 70% of TNBCs, but little is known regarding the genetic drivers associated with this event. Here, we show somatic deletion of a region syntenic with human 5q33.2–35.3 in a mouse model of TNBC. Mechanistically, we identify *KIBRA* as a major factor contributing to the effects of 5q loss on tumor growth and metastatic progression. Re-expression of *KIBRA* impairs metastasis *in vivo* and inhibits tumorsphere formation by TNBC cells *in vitro*. *KIBRA* functions co-operatively with the protein tyrosine phosphatase *PTPN14* to trigger mechanotransduction-regulated signals that inhibit the nuclear localization of oncogenic transcriptional co-activators YAP/TAZ. Our results argue that the selective advantage produced by 5q loss involves reduced dosage of *KIBRA*, promoting oncogenic functioning of YAP/TAZ in TNBC.

INTRODUCTION

Approximately 15% of patients with invasive breast cancer are diagnosed with triple-negative breast cancer (TNBC), defined by the absence of estrogen receptor (ER), progesterone receptor (PR), and human epidermal growth factor receptor 2 (HER2) expression (Foulkes et al., 2010). Because TNBC lacks an approved targeted therapy, the only systemic treatment is chemotherapy. Although this can induce a complete pathological response, TNBCs are associated with a high risk of early recurrence, and metastatic disease is virtually incurable (Denkert et al., 2017; Foulkes et al., 2010).

A concerted effort has been undertaken to understand the molecular basis of TNBC heterogeneity and discover actionable targets. Molecular subtyping based on gene expression has defined the majority of TNBCs as basal-like (49%–80%) (Denkert et al., 2017; Lehmann and Pietenpol, 2014; Rakha et al., 2009) or claudin-low (up to ~30%) (Prat et al., 2010; Prat and Perou, 2011). Further studies have refined this classification into four subtypes: basal-like 1, basal-like 2, mesenchymal, and luminal androgen receptor (Lehmann et al., 2016). Integrating mutation status, gene expression, and copy number has shown that breast cancers segregate into 10 "integrative clusters" (Curtis et al., 2012). Most TNBCs (60%) fall into integrative cluster 10 (IntClust10), associated with an elevated 5-year risk of recurrence and frequent *TP53* mutations. Up to 70% of TNBCs also undergo deletions on the long arm of chromosome 5, spanning 5q11 to 5q35 (Johannsdottir et al., 2006; Natrajan et al., 2009; Turner et al., 2010). However, with few exceptions (Weigman et al., 2012), genes conferring selective pressure for 5q loss are relatively unknown.

Genetically engineered mouse models are powerful tools for deciphering breast cancer complexity (Cardiff et al., 2000; Herschkowitz et al., 2007). We have previously shown that mammary tumors driven by mouse mammary tumor virus (MMTV)-*Met* reflect human breast cancer subtypes, including basal-like (Ponzo et al., 2009), whereas conditional deletion of *Trp53* in this model (MMTV-*Met;Trp53fl/+;Cre*) induces mesenchymal tumors modeling the TNBC subtype claudin-low (Knight et al., 2013). Here we show that MMTV-*Met;Trp53fl/+;Cre* mammary tumors spontaneously lose a region on chromosome 11 that is syntenic with human 5q33.2–35.3. Using gene expression and functional analysis, we show that *WWC1* (*KIBRA*), a scaffold

This is an open access article under the CC BY license (http://creativecommons.org/licenses/by/4.0/).

protein and activator of the Hippo pathway located on 5q (Baumgartner et al., 2010; Genevet et al., 2010; Yu et al., 2010), has tumor- and metastasis-suppressive properties. Our data indicate a multifaceted role of KIBRA upstream of both canonical Hippo signaling and cytoskeletal cues that regulate the activity of the transcriptional coactivators YAP/TAZ.

RESULTS

Chromosome 5q Loss, a Frequent Event in Human TNBC, Is Modeled in Mouse Mammary Tumors

A powerful way to discover genes with causal roles in oncogenesis is to identify frequently altered genomic regions. Applying this approach to TNBC mouse models, we used array-comparative genomic hybridization (aCGH) to identify a region on chromosome 11 that is lost in 18 of 19 MMTV-*Met;Trp53fl/+;Cre* and *Trp53fl/+;Cre* tumors (Knight et al., 2013; Figures 1A and S1) but not MMTV-*Met* tumors (Ponzo et al., 2009), with one exception (Figure S1, 5482; Ponzo et al., 2009). Because the size of the affected region varied, we identified a minimal common region (MCR) of loss extending from 18.9 to 49.8 Mb (Figures 1A and S1).

Mouse chromosome 11:31.4–49.8 Mb is syntenic with human 5q33.2–35.3 (Figure 1B), which is frequently lost in TNBC (Table S1). We used The Cancer Genome Atlas breast cancer patient dataset (Cancer Genome Atlas Network, 2012) to explore the extent of 5q loss among basal and claudin-low subtypes, representing the majority of TNBCs. Segmental losses spanning the entire 5q arm were frequent, with 40%–55% of tumors showing loss of 5q33.2–35.3 (Figure S2A). To identify candidate tumor suppressor genes within 5q, we analyzed 88 mouse-human gene homologs from the syntenic region (Table S2). Because gene expression and copy number alteration are not always correlative, we analyzed their expression in our mouse models, finding 13 genes (orthologous to 11 unique human genes) that were significantly decreased in tumors with loss of the MCR (Figure 1C; Table S3). Analysis of copy number and expression data, available for 10 of these genes, confirmed their hemizygous deletion in 40%–50% of human claudin-low and basal breast cancers (Figure 1D), although only 4 of 10 had negative mRNA *Z* scores, consistent with decreased expression (Figure 1E). Furthermore, only *CCNG1*, *CLINT1*, and *WWC1* had significantly decreased expression in basal and claudin-low patients (Figure 1F). To corroborate our findings, we used the Cancer Cell Line Encyclopedia (CCLE) to analyze expression in human cell lines representing breast cancer subtypes. Although *CCNG1* mRNA levels were universally low irrespective of subtype, and *CLINT1* levels did not vary significantly, basal B (claudin-low) cell lines had significantly lower expression of *WWC1* (also known as *KIBRA*) (Figure S2B). This is consistent with a previous observation associating low *WWC1* expression with a claudin-low phenotype (Moleirinho et al., 2013).

Depletion of *WWC1/KIBRA*, a 5q Gene, Increases the Metastatic Aggressivity of Mouse Breast Cancer Cells

Low *KIBRA* expression in murine and human basal B cell lines was validated by real-time qPCR and western blotting (Figures S2C and S2D). *KIBRA* encodes a multi-domain scaffold protein (Kremerskothen et al., 2003) acting upstream of the Hippo tumor suppressor pathway, interacting with MERLIN and LATS1/2 to inhibit the oncogenic transcriptional co-activators YAP/TAZ (Baumgartner et al., 2010; Genevet et al., 2010; Yu et al., 2010). To understand the role of *KIBRA* loss, we silenced *Kibra* in cells from an MMTV-*Met* tumor, 5156, which retain chromosome 11 (Figure 2A). These cells were transduced with a luciferase-expressing lentivirus and orthotopically injected into nude mice. We observed no difference in primary tumor growth between control and *Kibra* knockdown cohorts (Figure S3). Because breast cancer morbidity and mortality are caused primarily by metastasis, and TNBC is highly metastatic, we resected primary mammary tumors and monitored mice for metastasis using bioluminescence imaging (Figure 2B). Compared with controls, tumors with *Kibra* silencing had an elevated capacity to metastasize to lungs and lymph nodes (Figures 2B and 2C). To determine whether this was due to increased invasion, we grew cells as 3D cyst-like structures and monitored their ability to invade a surrounding type I collagen matrix. *Kibra* knockdown significantly increased the percentage of cysts displaying invasion (Figure 2D). Accordingly, *Kibra* silencing also enhanced cell migration in two dimensions (Figure 2E). These data support a role for KIBRA in suppressing metastatic dissemination.

Kibra Expression in Mouse Breast Cancer Cells Decreases Metastatic Potential

To further understand the role of *KIBRA* loss in TNBCs, we overexpressed *Kibra* in MMTV-*Met;Trp53fl/+;Cre* tumor cells (A1005 and A1034) with spontaneous loss of chromosome 11 (Figure 3Ai). *Kibra* expression altered cell morphology (Figure 3Aii) and decreased proliferation *in vitro* (Figure 3B), and tumor cells grown orthotopically had altered pathology and decreased growth (Figure 3C). Interestingly, *Kibra*-positive tumors also displayed a significant increase in polyploidy (Figure 3C). This may be due to an increased rate of cytokinesis failure, providing an explanation for the reduced growth and smaller size of *Kibra*-positive tumors compared with controls (Figure 3C).

Because *KIBRA* knockdown in MCF10A cells induces EMT (epithelial-to-mesenchymal transition) (Moleirinho et al., 2013), we used real-time qPCR to determine whether *Kibra* expression modulates the expression of EMT regulators. Although *Kibra* expression significantly decreased the mRNA levels of *Twist2* (a transcriptional driver of EMT), it also increased the expression of its homolog *Twist1*, with no effect on other EMT drivers (Figure S4). Despite this, the mRNA levels of *E-cadherin* (*Cdh1*) and *Claudin-1* (*Cldn1*) were elevated upon *Kibra* expression, linking *Kibra* to an epithelial phenotype. These observations are reflected in the Cancer Genome Atlas (TCGA) dataset, where *KIBRA* and *CDH1* mRNA levels positively correlate in basal breast tumors, but no anti-correlation between *KIBRA* and EMT drivers is apparent (Figure S4).

Because *Kibra* depletion increased metastatic potential, we investigated the effect of *Kibra* re-expression on metastasis. Because spontaneous metastasis of A1005 cells from the mammary gland is variable, we injected them into the tail vein (Figure 3D). Strikingly, control cells disseminated extensively to sites outside of the lung 2 weeks post-injection. This was strongly suppressed by *Kibra* expression (Figure 3D), indicating that, although *Kibra*-positive cells survive and grow in the lung

OPEN ACCESS
CellPress

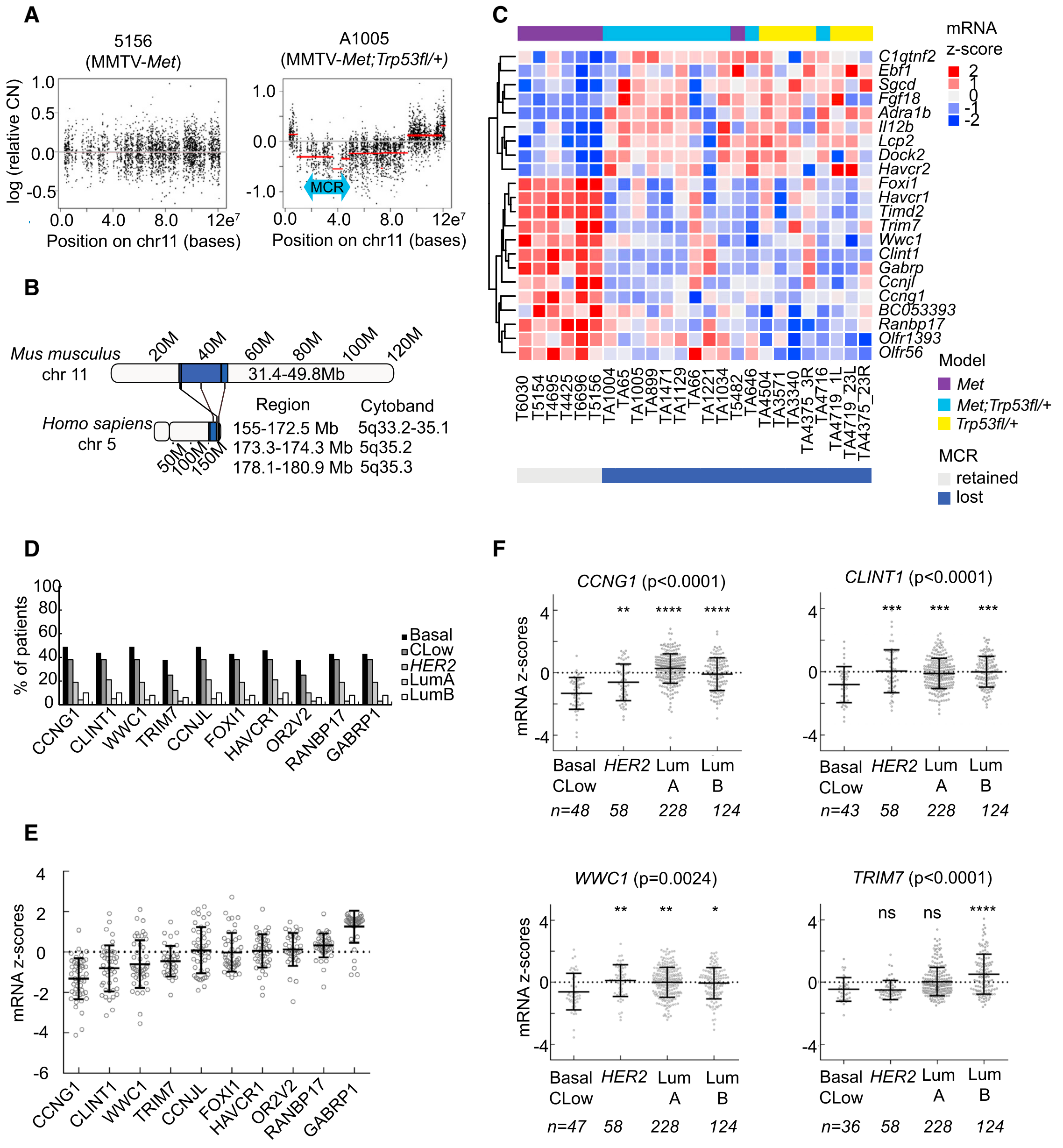

Figure 1. Loss of Heterozygosity in Mouse Mammary Tumors Mimics Chromosome 5q Loss, a Frequent Event in Human TNBC

(A) Example aCGH profiles of chromosome (chr) 11 in MMTV-*Met* (5156) and MMTV-*Met;Trp53fl/+;Cre* (A1005) mammary tumors. Black dots indicate individual microarray probes and red lines segmented means for regions deviating from a log copy number change of 0. The blue arrow indicates a minimal common region (MCR) of loss from 18.9–49.8 Mb.
(B) Alignment of the MCR with human chr 5q.
(C) Heatmap showing significant differential expression among mouse model tumors, with decreased expression of 13 genes in tumors with loss of the MCR.
(D) Frequency of hemizygous deletion for 10 of 11 genes across PAM50 and claudin-low (CLow) breast cancer subtypes in TCGA data.
(E) TCGA mRNA *Z* scores for all 10 genes among basal and claudin-low tumors with hemizygous loss.
(F) TCGA mRNA *Z* scores for all molecular subtypes. Asterisks indicate statistical significance for differences in mRNA levels between basal/claudin-low tumors with copy number loss and other PAM50 subtypes. n = number of patients.
See also Figures S1 and S2 and Tables S2 and S3.

parenchyma, they are unable either to re-enter the bloodstream, survive in the circulation, or establish in sites other than the lungs, pre-requisites for further metastatic dissemination. Supporting an anti-metastatic function of *Kibra*, its expression decreased the invasion of a 3D collagen matrix by A1005 cells (Figure 3E). Together, these *in vivo* and *in vitro* assays demonstrate a metastasis-suppressive role for *Kibra*, consistent with its frequent loss in TNBCs.

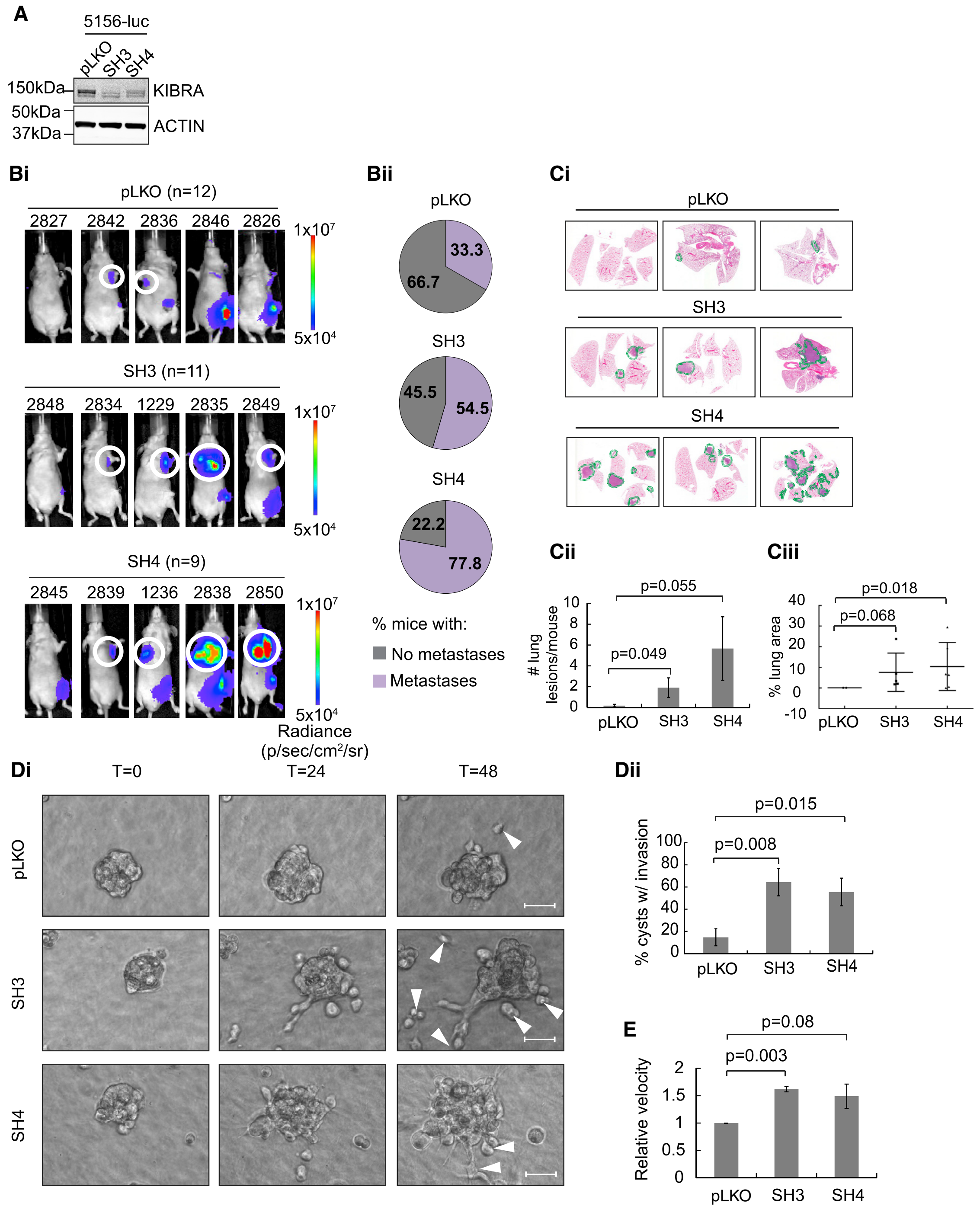

Figure 2. *Kibra* Silencing Increases Tumor Cell Aggressivity in Mice

(A) Knockdown of *Kibra* in the MMTV-*Met* mammary tumor cell line 5156-luciferase (5156-luc). Two independent shRNAs (SH3 and SH4) are compared with a pLKO-empty vector control.

(Bi) 5156-luc cells were orthotopically injected and resected after 5 weeks. Representative bioluminescence images of metastatic dissemination are shown. Metastases (white circles) were confirmed in histological sections. n = number of mice.

(Bii) Percentages of mice with confirmed lung and lymph node metastases.

(Ci) H&E-stained lung sections from 3 representative mice per condition. Metastatic lesions are outlined in green.

(Cii) Quantification of lung metastatic burden.

(Ciii) Calculation of the lung area containing tumor (mean ± SEM).

(legend continued on next page)

KIBRA Expression Inhibits Tumorsphere Formation in Human TNBC Cell Lines

To determine its effect on the biology of human TNBC, we re-expressed *KIBRA* in 3 TNBC cell lines (Figure 4Ai). As with murine TNBC, *KIBRA* expression altered the morphology, decreased proliferation, and decreased the ability to invade a collagen matrix (Figures 4A–4C). To examine how *KIBRA* influenced tumorigenic capacity, we grew cells under conditions of anoikis, as tumorspheres, to assay their tumor-initiating capacity and stem-like properties (Pece et al., 2010). *KIBRA* expression dramatically decreased tumorsphere propagation (Figure 4D; Figures S5A and S5B). Because sphere-forming efficiency (SFE) can indicate both tumorigenic and metastatic potential (i.e., the ability to seed, survive, and propagate at a secondary site), this is consistent with the role of *KIBRA* as a metastasis suppressor. Overall, these data show that *KIBRA* suppresses the tumorigenic and metastatic potential in TNBC cells and, therefore, that its loss can confer significant advantages to triple-negative tumors.

Inhibition of Tumorsphere Formation by KIBRA Requires the WW1/2 Domains

To identify molecular mechanisms by which *KIBRA* functions as a tumor/metastasis suppressor, we systematically deleted regions of protein-protein interaction and structural regions and determined their role in tumorsphere formation (Figures S5C and S5D). MDA-MB-231 cells expressing wild-type KIBRA or mutants lacking the PSD95/DLG1/ZO-1 (PDZ)/atypical protein kinase C (aPKC) binding, Glu-rich, or C2 regions displayed reduced SFE compared with the empty vector control (Figure 4E). In contrast, KIBRA mutants lacking the WW1/2 domains did not impair tumorsphere formation, implicating proteins binding the KIBRA WW domains in the repression of tumorsphere formation.

Several studies have shown that increased TAZ activation endows mammary gland cells with stem-like properties (Bartucci et al., 2015; Cordenonsi et al., 2011). To examine the role of KIBRA in inhibiting YAP/TAZ, we initially examined the expression of a YAP/TAZ signature (Cordenonsi et al., 2011) in TCGA breast cancer data. Claudin-low and basal tumors with *KIBRA* copy number loss showed enrichment of this signature compared with those without *KIBRA* loss or other PAM50 subtypes (Figure 4Fi), suggesting that *KIBRA* loss increases YAP/TAZ activity in tumors with 5q deletion. Accordingly, *KIBRA* expression induced a significant WW domain-dependent decrease in mRNA levels of YAP/TAZ transcriptional targets (*CYR61* and *CTGF*) in MDA-MB-231 cells (Figure 4Fii). To examine this further, we assayed the effect of KIBRA on nuclear accumulation of YAP/TAZ using immunofluorescence. Using a stiffness-tenable polyacrylamide culture platform mimicking the mechanical rigidities of healthy and diseased breast tissue (Engler et al., 2006; Levental et al., 2009), we exploited the ability of YAP/TAZ to translocate to the nucleus in response to increasing extracellular matrix (ECM) stiffness (Dupont et al., 2011). Importantly, this allowed us to assay single cells, alleviating variability induced by changes in cell-cell contact. Compared with controls, KIBRA expression severely diminished nuclear YAP/TAZ localization in MDA-MB-231 and A1005 cells on stiff ECM (Figures 5A and 5B), an effect abrogated by deletion of the WW domains (Figure 5A). These data demonstrate that KIBRA prevents mechanotransduction-dependent nuclear accumulation of YAP/TAZ in a manner dependent on interaction(s) with its WW domains.

Although YAP and TAZ are generally considered to functionally overlap, it is *TAZ* specifically that is amplified in basal-like breast cancer and is associated with stem-like characteristics and metastatic potential (Chan et al., 2008; Cordenonsi et al., 2011; Skibinski et al., 2014). To determine the effect of KIBRA expression on YAP and TAZ, we used specific antibodies to examine their status in MDA-MB-231 and A1005 cells (Figure 5C). In agreement with previously published work (Xiao et al., 2011), we detected elevated YAP phosphorylation at Ser127 in cells expressing KIBRA, indicating inhibition. However, the increase in MDA-MB-231 cells was slight and, in A1005, correlated with increased YAP protein levels. More significantly, we observed a decrease in TAZ protein levels upon KIBRA expression in both cell lines (Figure 5C), which is consistent with the proteasomal degradation of TAZ that occurs following either Hippo pathway activation (Liu et al., 2010) or interference with mechanotransduction (Sorrentino et al., 2014).

To investigate the possibility that KIBRA functions through TAZ inhibition, we grew KIBRA-expressing cells as tumorspheres after transfection with constitutively active, serine-to-alanine mutants of YAP and TAZ (Figure 5Di), for which we confirmed nuclear localization (Figure S5E). Constitutively active TAZ, but not YAP, significantly increased the SFE of KIBRA-expressing cells (Figures 5Dii and 5Diii). In further support of a role for TAZ inhibition downstream of KIBRA, orthotopic A1005 tumors (Figure 3) showed prominent nuclear localization of TAZ that became cytoplasmic in tumors expressing KIBRA. YAP, however, remained largely cytoplasmic under all conditions (Figure 5E). Collectively, these data indicate that, in claudin-low breast cancer cells, loss of KIBRA promotes tumor progression and metastasis primarily by relieving inhibition of TAZ.

KIBRA and PTPN14 Co-operate to Impair Breast Cancer Tumorsphere Formation

To clarify the WW domain interactions critical for KIBRA to suppress tumorsphere formation, we used BioID, a proximity-based strategy using biotinylation and mass spectrometry, for analysis of proximity-dependent interactions (Roux et al., 2012). Figure 6A, i, and Table S4 show high-confidence interactors (significance analysis of interactome [SAINT]express < 0.8) enriched in KIBRA BioID compared with negative controls in MDA-MB-231 cells. The only significant association lost by ΔWW1/2-KIBRA but retained in "non-rescue" mutants (lacking PDZ and aPKC binding domains), was with PTPN14 (protein tyrosine

(Di) Representative images of invasion (white arrows) from cysts into the collagen matrix. Scale bars represent 50 μm.
(Dii) Quantification of invasion (3 independent experiments, means ± SEM).
(E) Migration velocity of cells on fibronectin-coated plates (3 independent experiments, 30 cells/condition/experiment, mean ± SEM).
See also Figure S3.

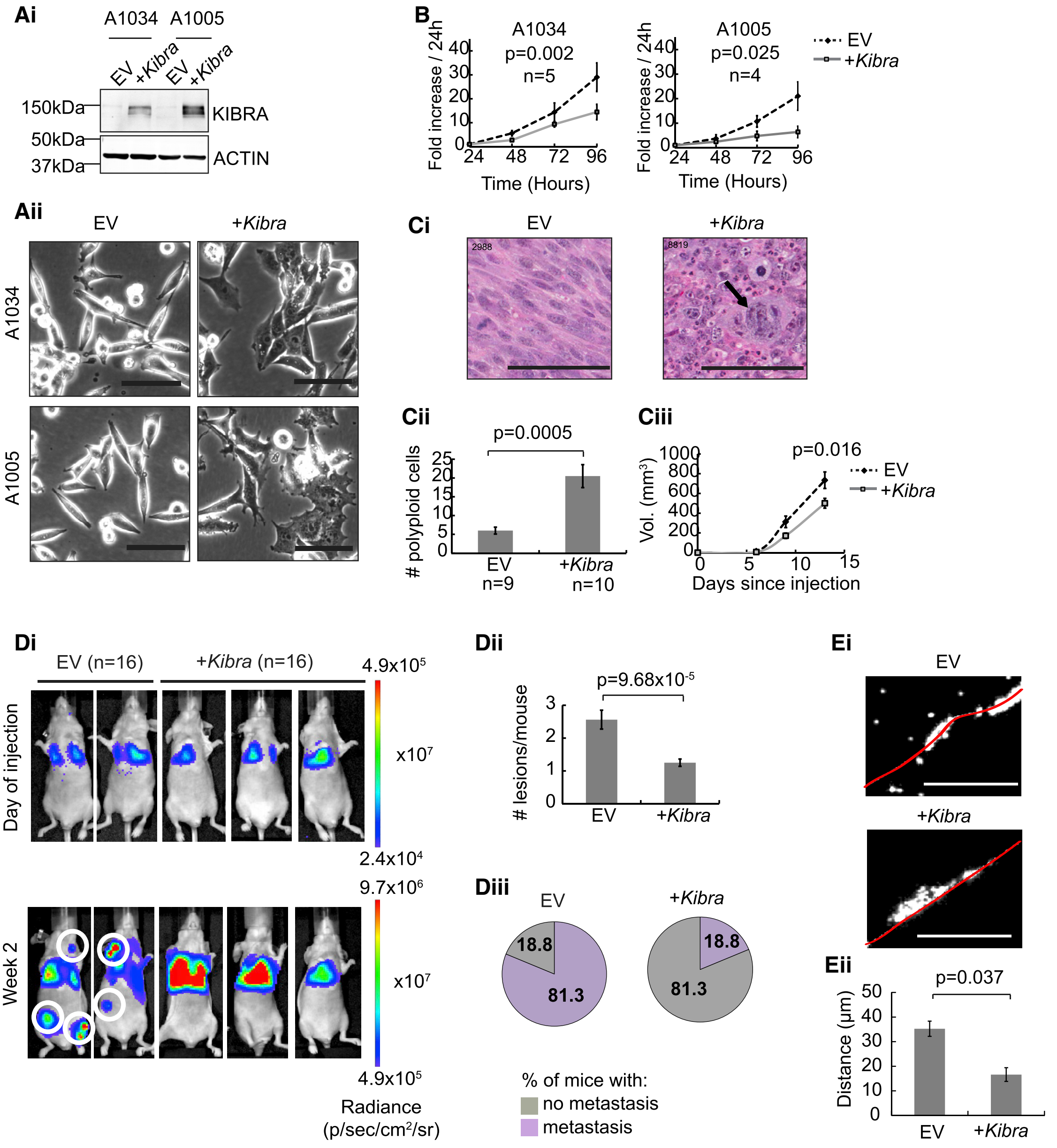

Figure 3. *Kibra* Re-expression Has an Anti-tumorigenic Effect

(Ai) Western blot showing stable KIBRA re-expression in MMTV-*Met;Trp53fl/+;Cre* mammary tumor cells (A1034 and A1005).

(Aii) Altered cell morphology in *Kibra*-expressing cells. EV, empty vector control. Scale bars, 100 μm.

(B) Proliferation of cell lines with or without *Kibra*. Shown is the mean of the indicated replicates ± SEM.

(Ci) H&E-stained mammary tumor sections from mice orthotopically injected with A1005 cells with or without *Kibra*. The arrow indicates an example of polyploidy. Scale bars, 100 μm.

(Cii) Quantification of karyomegalic/multi-nucleated (polyploid) cells per section (mean ± SEM).

(Ciii) Growth of tumors from (Ci) (mean ± SEM), showing significant difference in endpoint tumor size.

(Di) Representative bioluminescent images of mice immediately after and 2 weeks after intravenous injection of A1005-luciferase cells with or without *Kibra*. n = number of mice. White circles highlight metastases outside of the lungs.

(Dii) Number of metastatic sites per mouse (mean ± SEM).

(Diii) Percentage of mice with metastatic sites outside of the lungs (mean ± SEM).

(Ei) Representative images of invasion of DAPI-stained (white) A1005 cells with or without *Kibra*. Scale bars, 250 μm.

(Eii) Quantification of invasion as distance traveled through collagen from the seeded area (red line in Ei) (n = 3, mean ± SEM).

See also Figure S4.

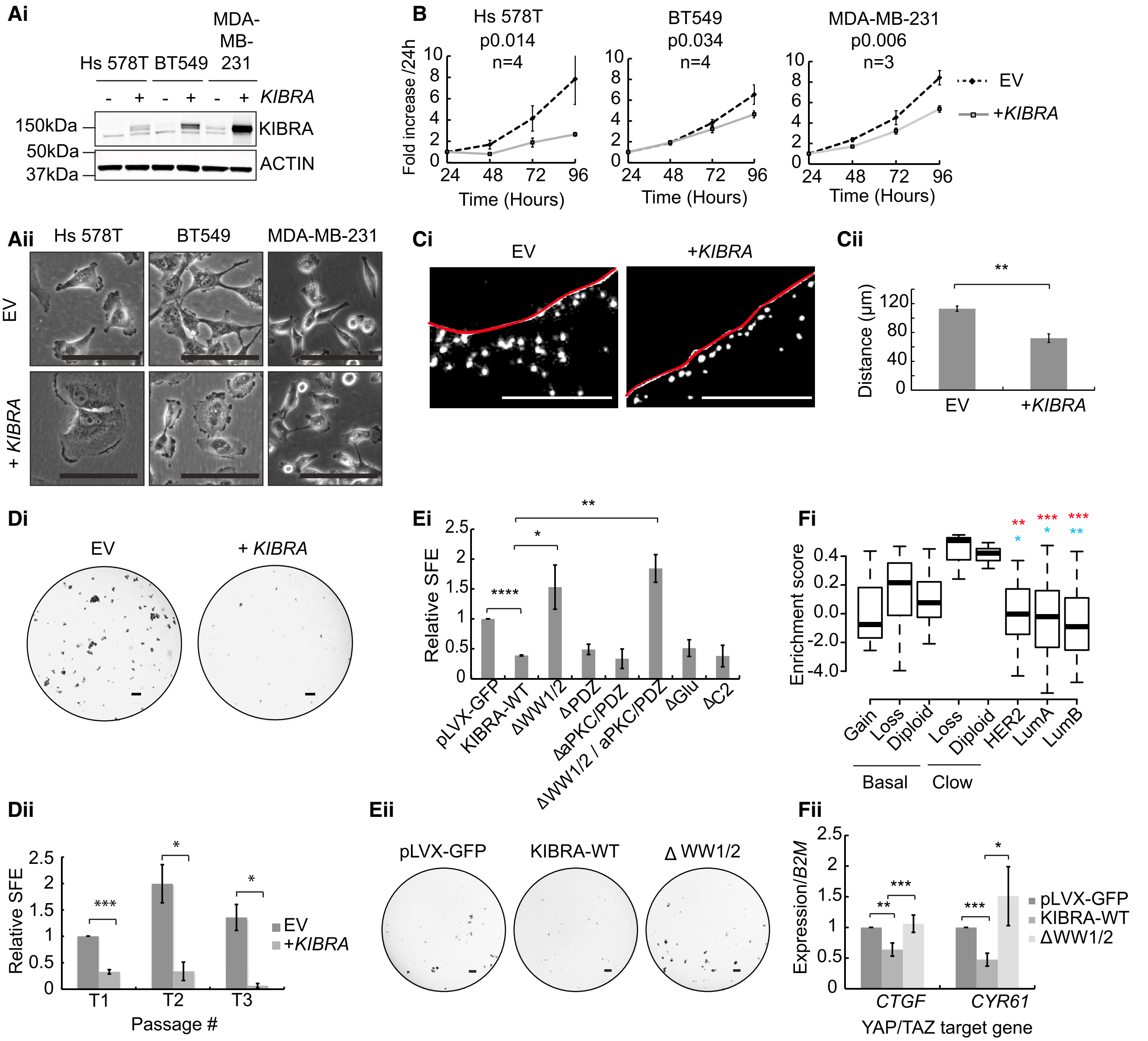

Figure 4. KIBRA Expression Reduces the Invasiveness and Tumorsphere-Forming Capacity of Breast Cancer Cells and Correlates with a YAP/TAZ Signature in Human Breast Cancers

(Ai) Western blot showing stable KIBRA expression in 3 basal B breast cancer cell lines.

(Aii) Images showing *KIBRA*-induced loss of mesenchymal features. Scale bars, 100 µm.

(B) Proliferation with or without *KIBRA*. Shown are the mean values of the indicated replicates ± SEM.

(Ci) Representative images of invasion of DAPI-stained (white) MDA-MB-231 cells with or without *KIBRA*. Scale bars, 250 µm.

(Cii) Quantification of invasion as distance traveled through collagen from the seeded area (red line). n = 3, mean ± SEM.

(Di) Representative images of MDA-MB-231 tumorspheres with or without *KIBRA*. Scale bars, 400 µm.

(Dii) Sphere-forming efficiency (SFE) calculated at 3 serial passages (T1, T2, and T3) and normalized to EV control at T1 (n = 3, mean ± SEM).

(Ei) SFE for MDA-MB-231 cells expressing a control (pLVX-GFP) compared with cells expressing either wild-type KIBRA (KIBRA-WT) or KIBRA mutants lacking specific regions as indicated (n = 3, mean ± SEM).

(Eii) Representative images of tumorspheres in (Ei). Scale bars, 400 µm.

(Fi) Gene set variation analysis (GSVA) showing enrichment of a *YAP/TAZ* gene expression signature. Basal and claudin-low subtypes are divided by *KIBRA* copy number gain or loss or diploid status. Asterisks indicate statistical differences between PAM50 subtypes and claudin-low (blue) or basal (red) tumors affected by *KIBRA* copy number loss.

(Fii) qRT-PCR for YAP/TAZ targets in MDA-MB-231 cells expressing vector control, KIBRA-WT, or ΔWW1/2-KIBRA (n = 3, mean ± SEM).

See also Figure S5.

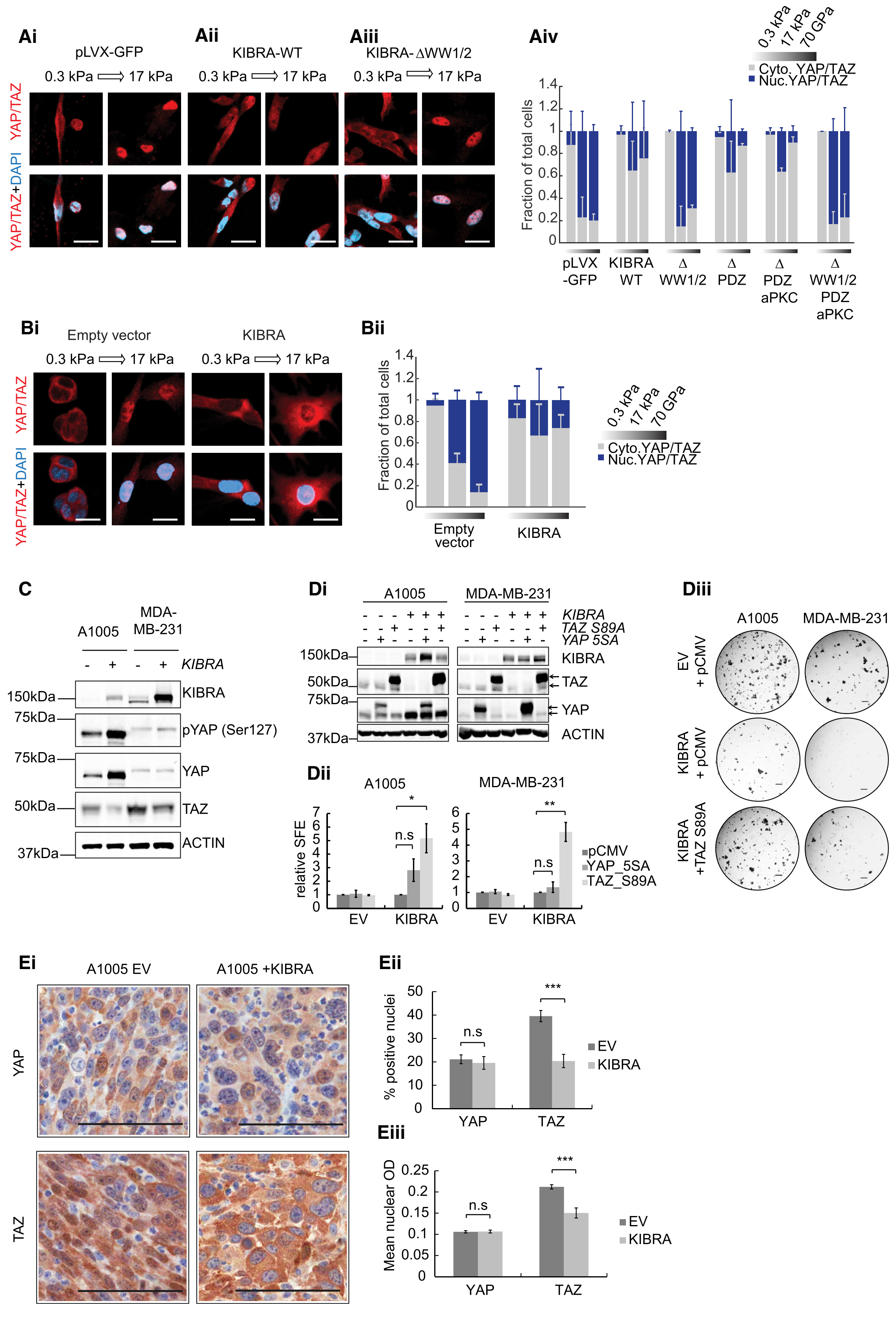

(legend on next page)

phosphatase non-receptor 14) (Poernbacher et al., 2012). We validated this interaction by co-immunoprecipitation (Figure 6Aii). Notably, the BioID failed to detect other known KIBRA interactors, including MERLIN and LATS1/2 (which were readily detected in other cell types such as HeLa; data not shown). Although MDA-MB-231 cells express LATS1, LATS2 is barely detectable, and MERLIN is not expressed (Figure S6A). Consistent with previous work (Xiao et al., 2011), KIBRA expression increased the levels of LATS1 and LATS2. However, KIBRA did not induce their auto-phosphorylation, indicating that KIBRA does not activate LATS1/2 in MDA-MB-231 cells (Figure S6A), possibly because of the absence of MERLIN (Baumgartner et al., 2010; Genevet et al., 2010; Yu et al., 2010). Interestingly, copy number loss of *KIBRA* can co-occur with that of *LATS1/2* or *NF2* (MERLIN), supporting LATS1/2 and MERLIN-independent functions of KIBRA in TNBCs (Cancer Genome Atlas Network, 2012; Figure S6B).

To investigate the role of the PTPN14-KIBRA interaction, we stably silenced *PTPN14* in MDA-MB-231 cells, expressed GFP-KIBRA, and seeded GFP-positive cells in tumorsphere assays (Figure 6Bi). KIBRA expression in control cells reduced SFE by 85%, which was rescued by *PTPN14* silencing in a manner correlative with the extent of knockdown (Figure 6B). This supports the hypothesis that PTPN14 co-operates with KIBRA to inhibit tumorsphere formation in MDA-MB-231 cells. To determine the role of the KIBRA/PTPN14 interaction in YAP/TAZ regulation, we evaluated YAP/TAZ subcellular localization in MDA-MB-231 cells, which express high levels of TAZ that decrease in response to KIBRA expression (Figure 5C). Strikingly, *PTPN14* silencing elicited a near-complete rescue of YAP/TAZ nuclear localization in KIBRA-expressing cells (Figure 6C), demonstrating co-operativity between KIBRA and PTPN14 in cytoplasmic sequestration of YAP/TAZ. This is supported by a highly significant correlation between *KIBRA* and *PTPN14* mRNA levels in basal and claudin-low tumors (Figure 6D).

KIBRA and PTPN14 Promote YAP/TAZ Cytoplasmic Sequestration through Regulation of Actin Cytoskeletal Dynamics

The regulation of YAP/TAZ localization by matrix tension or cell density involves modulation of the actin cytoskeleton (Aragona et al., 2013; Dupont et al., 2011). Consistent with this, expression of wild-type KIBRA, but not ΔWW1/2-KIBRA, decreased both actin stress fibers and nuclear localization of YAP/TAZ in MDA-MB-231 (Figures 7Ai and 7Bi) and A1005 cells (Figure S7) under stiff matrix conditions. These phenotypes were rescued by *PTPN14* silencing in wild-type KIBRA-expressing cells (Figures 7Aii, and 7Bii), demonstrating co-operativity between KIBRA and PTPN14 in regulating actin cytoskeletal dynamics to sequester YAP/TAZ in the cytoplasm. Furthermore, *Ptpn14* knockdown increased the metastasis of A1005 cells expressing *Kibra* to sites outside of the lungs (Figure S7), supporting the role of the KIBRA-PTPN14 interaction in suppressing metastasis *in vivo*.

The formation of actin stress fibers is controlled by RHOA, which activates formins that assemble F-actin and Rho-associated kinase (ROCK), which is required for stress fiber contractility (Narumiya et al., 2009). RHOA activation is therefore strongly implicated in YAP/TAZ nuclear localization caused by ECM stiffness (Dupont et al., 2011). We used Rhotekin-glutathione S-transferase (GST) pull-down assays (Ren et al., 1999) and an ELISA-based assay to detect guanosine triphosphate (GTP)-bound RHOA in cells expressing KIBRA (Figure 7C). Consistent with loss of stress fibers, KIBRA expression in MDA-MB-231 and A1005 cells decreased RHOA activity (Figure 7Ci; Figures S7Bi and S7Bii). This effect was not observed with ΔWW1/2 KIBRA (Figures 7Cii and 7Ciii), suggesting that the KIBRA-PTPN14 interaction represses RHOA activity to impair mechanotransduction-based regulation of TAZ, as shown schematically in Figure 7D.

DISCUSSION

The identification of syntenic regions of chromosomal loss in mouse cancer models and the human tumors they represent can aid in the identification of tumor suppressor genes (Liu et al., 2016; Xue et al., 2012). Here we have applied this strategy to show that mammary tumors from the MMTV-*Met;Trp53fl/+;Cre* mouse model lose a chromosomal region syntenic with human 5q33.2–35.3. Using a multifaceted approach, we identified *KIBRA* as a suppressor not only of tumor growth but also of metastasis. Selective pressure for loss of metastasis suppressor genes during tumorigenesis has been

Figure 5. Anti-tumorigenic Effects of KIBRA Are Associated with a Reduction in TAZ Protein Levels and Inhibition of TAZ Nuclear Localization

(Ai–Aiii) Subcellular localization of YAP/TAZ in MDA-MB-231 cells expressing wild-type KIBRA (KIBRA-WT), ΔWW1/2-KIBRA, or control (pLVX-GFP) in response to increasing matrix tension (0.3 to 17 kPa). Scale bars, 20 μm.

(Aiv) Quantification of nuclear to cytoplasmic YAP/TAZ ratios under conditions of soft (0.3 kPa) or stiff (17 kPa) matrix or a collagen-coated glass coverslip (70 GPa) (n = 3, mean ± SD).

(Bi and Bii) Representative images and quantification of YAP/TAZ localization in A1005 cells in response to matrix tension as in (A).

(C) Western blot showing YAP phosphorylation and TAZ protein levels in MDA-MB-231 and A1005 cells with or without KIBRA.

(Di) Western blots confirming transfection of constitutively active YAP or TAZ in A1005 and MDA-MB-231 cells with KIBRA. The empty pCMV vector is a negative control. The arrows indicate tagged (top) and endogenous (bottom) proteins.

(Dii) Quantification of SFE relative to empty vector for cells in (Di) (n = 3, ± SEM).

(Diii) Representative tumorsphere images. Scale bars, 400 μm.

(Ei) Immunohistochemistry (IHC) showing YAP and TAZ subcellular localization in A1005 orthotopic tumors with or without *KIBRA*. Scale bars, 100 μm.

(Eii and Eiii) Percentage of cells with positive nuclear staining (ii) and mean optical density (OD) of nuclear staining (iii) for YAP and TAZ in ten fields of view, 6 to 10 sections per condition (mean ± SEM).

See also Figure S5.

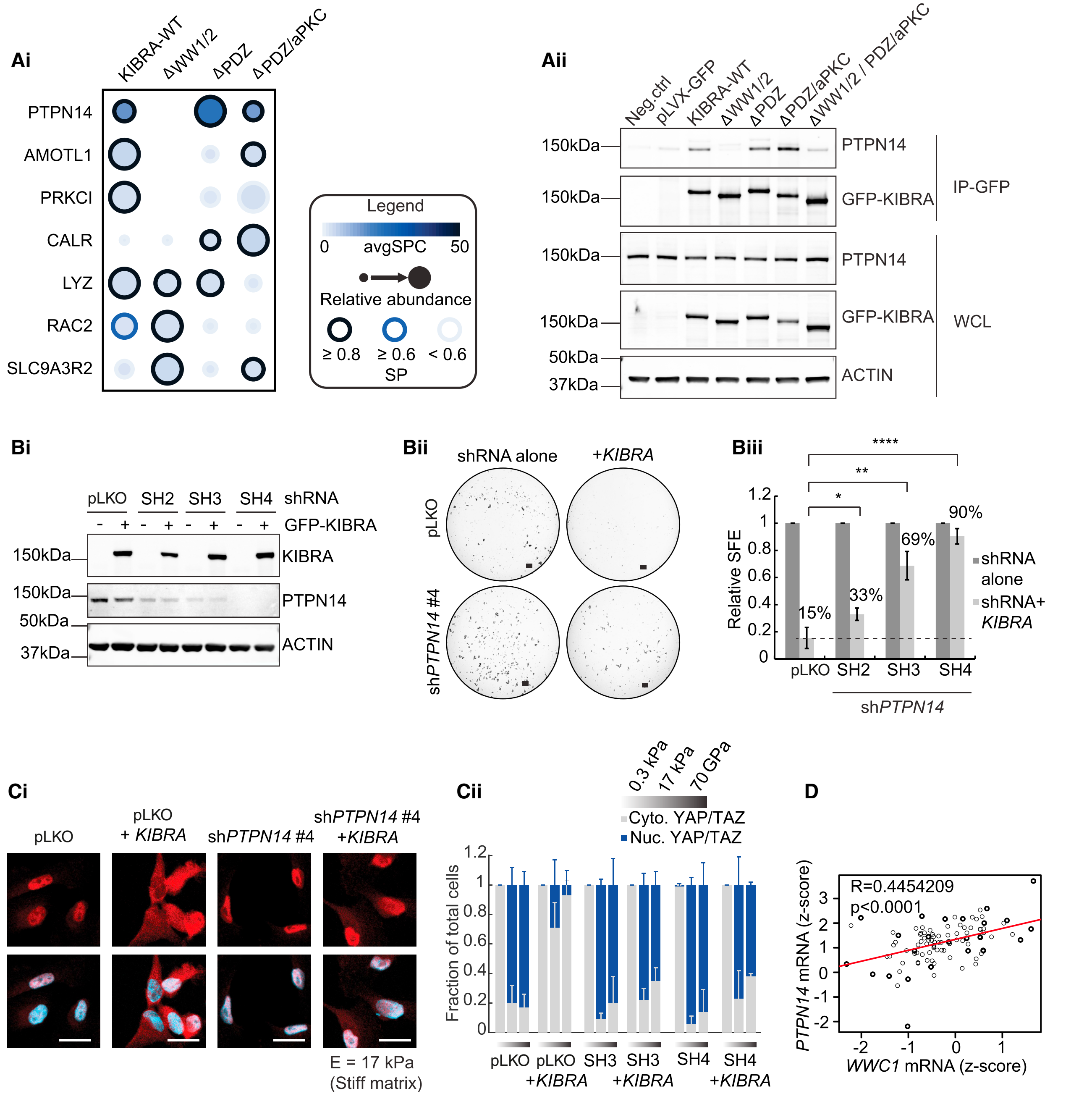

Figure 6. The KIBRA WW1/2 Domain Interactor PTPN14 Is Required for KIBRA-Mediated Inhibition of Tumorsphere Formation

(Ai) High-confidence KIBRA-proximal proteins from BioID mass spectrometry analysis of MDA-MB-231 cells expressing WT or mutated KIBRA.
(Aii) Co-immunoprecipitation of KIBRA and PTPN14 in MDA-MB-231 cells expressing wild-type or mutated KIBRA.
(Bi) Western blot showing PTPN14 levels in MDA-MB-231-KIBRA cells expressing 3 *PTPN14* shRNAs (SH2, SH3, and SH4) or empty vector (pLKO).
(Bii) Representative images of MDA-MB-231 tumorspheres expressing pLKO or *PTPN14* SH4 ± *KIBRA*. Scale bars, 400 μm.
(Biii) SFE of MDA-MB-231 cells expressing pLKO or *PTPN14* shRNA with or without *KIBRA*, normalized to the appropriate shRNA-alone condition (conditions seeded in triplicate, mean of 2 experiments ± SEM).
(Ci) YAP/TAZ localization in MDA-MB-231 cells expressing pLKO or *PTPN14* shRNA with or without *KIBRA*. Scale bars, 40 μm.
(Cii) Quantification of YAP/TAZ nuclear to cytoplasmic ratios in MDA-MB-231 cells expressing pLKO or *PTPN14* shRNA with or without *KIBRA*, cultured on soft (0.3 kPa) or stiff (17 kPa) matrix or collagen-coated glass coverslips (70 GPa) (n = 3 mean ± SD).
(D) Pearson correlation analysis of *WWC1* (*KIBRA*) and *PTPN14* mRNA levels (*Z* scores) in pooled basal and claudin-low patients (TCGA data, n = 89).
See also Figure S6 and Table S4.

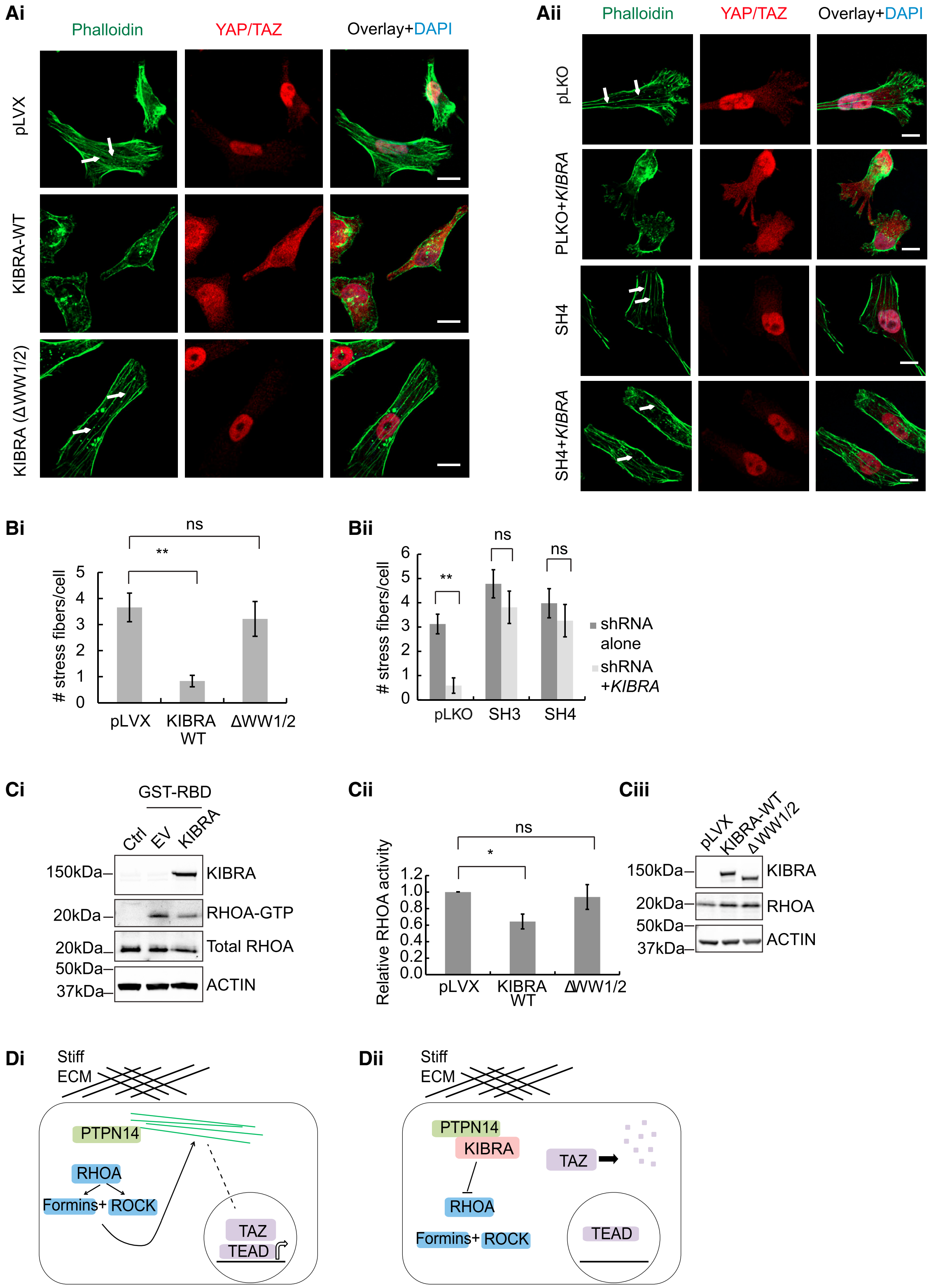

Figure 7. KIBRA and PTPN14 Co-operatively Regulate Actin Cytoskeletal Tension to Inhibit the Nuclear Translocation of YAP/TAZ

(Ai) Representative phalloidin staining and YAP/TAZ immunofluorescence of MDA-MB-231 cells expressing empty vector, wild-type KIBRA, or ΔWW1/2 KIBRA seeded on collagen-coated coverslips. White arrows indicate actin stress fibers. Scale bars, 10 μm.

(legend continued on next page)

described (Al-Mulla et al., 2006; Cohn et al., 1991) and may reflect functional overlap between tumor initiation and aspects of the metastatic cascade. For example, the ability to survive and self-renew could contribute to dissemination and establishment at a secondary site. Notably, however, selective pressure for 5q loss might also be conferred by co-operative effects because of loss of multiple genes, including *KIBRA*. Indeed, it has been suggested that loss of multiple DNA damage response and cell cycle genes upon 5q deletion may promote genomic instability and tumor progression (Curtis et al., 2012; Weigman et al., 2012). This may explain why the re-introduction of KIBRA alone has a modest effect on tumor growth *in vivo*.

Diminished expression of KIBRA has been detected in claudin-low breast cancers, leukemia, and osteosarcomas (Basu-Roy et al., 2015; Hill et al., 2011; Moleirinho et al., 2013), although the underlying mechanisms have not been fully explored. Much of the premise for KIBRA as a tumor suppressor comes from its role in activating the Hippo pathway, for which loss of function and the concomitant activation of YAP/TAZ are well-documented in TNBCs (Cordenonsi et al., 2011). Hypermethylation of the LATS1 and LATS2 promoters is observed in 50% of breast cancers (Takahashi et al., 2005), whereas genomic loss of LATS1, LATS2, and NF2 also occurs in TNBC (Figure S6B). Amplification of TAZ occurs in ~44% of basal breast cancers, where its expression confers stem-like and metastatic traits (Chan et al., 2008; Cordenonsi et al., 2011) and predicts poor outcome (Skibinski et al., 2014). Here, we provide evidence that hemizygous deletion of *KIBRA* increases TAZ activity in TNBC, with KIBRA expression inhibiting both tumorsphere formation (i.e., self-renewal of tumor-initiating cells) and the mechanosensing of a stiff ECM. The role of KIBRA in suppressing mechanical signals activating TAZ may be related to suppression of self-renewal, given that an undifferentiated stem-like state is maintained through contact with stiff ECM (Engler et al., 2006; Lui et al., 2012). Indeed, cells maintaining ECM contact in the basal layer of breast epithelium have nuclear TAZ, which becomes cytoplasmic as cells lose basement membrane contact and differentiate (Skibinski et al., 2014). KIBRA loss may constitutively activate mechanotransduction pathways that positively regulate TAZ, leading to persistent TAZ nuclear localization and maintenance of the poorly differentiated phenotype associated with basal-like tumors.

The mechanism of tumorsphere suppression by KIBRA involves its WW1/2 domain-mediated interaction with PTPN14. Although previous studies have shown that KIBRA and PTPN14 engage canonical Hippo signaling (Wilson et al., 2014), we found that they also co-operate to inhibit TAZ in MDA-MB-231 cells that lack MERLIN and activated LATS1/2 by inactivating RHOA and impairing actin stress fiber assembly. Although the metastasis suppressor phenotype conferred by KIBRA was only partially rescued by *Ptpn14* knockdown in A1005 cells, this may be due to residual inhibition of YAP/TAZ by canonical Hippo signaling, which, as we show, remains active in these cells. Hence, KIBRA inhibits YAP/TAZ via Hippo signaling or by activating the mechanotransduction-sensitive pathways that can promote YAP phosphorylation and TAZ degradation even in the absence of LATS1/2 and MERLIN (Sorrentino et al., 2014).

In addition to migration and invasion, cytoskeletal modulation by RHOA is critical for cytokinesis (Chircop, 2014). The accumulation of polyploid cells in A1005 KIBRA tumors may therefore involve decreased RHOA activity, which is known to cause growth arrest in tetraploid cells via activation of LATS2, subsequent YAP inhibition, and TP53 stabilization (Ganem et al., 2014). Although A1005 cells are *Trp53*-null, both LATS1/2 and YAP are phosphorylated upon KIBRA expression in A1005 cells (Figure S6), providing a partial mechanism by which KIBRA could impair growth.

Loss of heterozygosity (LOH) affecting large genomic regions occurs frequently in many cancers, including breast cancer (Solimini et al., 2012). The identification of genetic drivers for LOH and determination of their biological functions could provide new approaches for therapy. We demonstrate tumor-suppressive properties for the 5q gene *KIBRA*, which we link to tumor-initiating capacity and metastatic ability. We identify a Hippo pathway-independent function for KIBRA via its interaction with PTPN14, which itself has metastasis suppressor properties (Belle et al., 2015), in regulating YAP/TAZ localization through modulation of RHOA activity and the actin cytoskeleton. This contributes significantly to the understanding of cross-talk between actin cytoskeletal dynamics and YAP/TAZ function. The potential to target YAP/TAZ therapeutically, including through inhibiting mechanotransduction pathways, is currently being explored (Zanconato et al., 2016). Based on our findings, such therapeutic angles could be applied to TNBCs with 5q loss.

EXPERIMENTAL PROCEDURES

Genomic Analyses

Genomic DNA and mRNA isolation and microarrays were performed as described previously (Knight et al., 2013). Patient gene expression and copy number information were obtained from a TCGA Breast Invasive Carcinoma

(Aii) Representative immunofluorescence as in (Ai) for cells expressing pLKO control or sh*PTPN14* (SH4) with or without *KIBRA*.
(Bi and Bii) Number of stress fibers per cell for (Ai) and (Aii) (n = 3, mean ± SEM).
(Ci) Representative Rhotekin-GST pull-down in MDA-MB-231 cells expressing EV or KIBRA. GST alone was used as a control (Ctrl).
(Cii) RHOA activity determined by G-LISAs (n = 3, mean ± SEM).
(Ciii) RHOA protein levels for (Cii).
(D) Schematic diagram showing regulation of TAZ by KIBRA.
(Di) In the absence of KIBRA, stiff ECM activates RHOA, leading to actin stress fiber formation and contractility, facilitating TAZ nuclear translocation and interaction with TEA-domain (TEAD) transcription factors to promote expression of pro-oncogenic genes.
(Dii) Association of KIBRA with PTPN14 inhibits RHOA activation required for actin stress fiber assembly, removing the stimulus for nuclear translocation of TAZ and resulting in its proteasomal degradation.
See also Figure S7.

dataset (Cancer Genome Atlas Network, 2012; http://cancergenome.nih.gov). Further details can be found in the Supplemental Experimental Procedures.

Statistical Analyses

Statistical differences were calculated using Student's t tests, where significance is as follows: $p > 0.05$, not significant (ns); $^{*}p \leq 0.05$; $^{**}p \leq 0.01$; $^{***}p \leq 0.001$; $^{****}p \leq 0.0001$. Statistical significance in Figures 1F and 4F was calculated by one-way ANOVA with *post hoc* Tukey's multiple comparisons test. One luminal B patient-TCGA-E2-A155-01 was an outlier and was removed from all analyses.

Cell Culture

Mouse tumor cells were isolated and cultured as described previously (Knight et al., 2013). All human cell lines were obtained from the ATCC and cultured in DMEM (Hs578T and MDA-MB-231) or RPMI medium (BT549) with 10% fetal bovine serum (FBS). *In vitro* assays are described in the Supplemental Experimental Procedures.

Generation of Stable Cell Lines

The retroviral pBabe-*KIBRA* vector was a kind gift from Dr. Paul Reynolds (Addgene 40887). N-terminally GFP-tagged wild-type and mutant *KIBRA* were expressed from the pLVX lentiviral vector. Short hairpin RNAs (shRNAs) were expressed from pLKO.1 (Sigma-Aldrich). Further details can be found in the Supplemental Experimental Procedures.

Transient Transfections

The vectors pCMV-FLAG YAP2 5SA (Kunliang Guan, Addgene 27371) and 3XFLAG pCMV-TOPO TAZ (S89A) (Jeff Wrana, Addgene 24815) were used. The empty pCMV vector was a negative control. Cells were transfected using Lipofectamine 3000 (Invitrogen) according to the manufacturer's instructions. Further details can be found in the Supplemental Experimental Procedures.

KIBRA Mutagenesis

KIBRA mutants were generated using Q5 site-directed mutagenesis (New England Biolabs) on a pENTR11-wild-type KIBRA vector, as detailed in the Supplemental Experimental Procedures.

In Vivo Studies

All procedures involving mice were reviewed and approved by the McGill University Facility Animal Care Committee (FACC) and performed in accordance with university and national guidelines. Female 6-week-old friend virus B/NIH (FVB/N) mice were used for orthotopic mammary tumor growth experiments, and female 6-week-old NCr athymic nude mice (Taconic) were used for metastasis assays (tail vein injection and primary tumor resection assays). Bioluminescence imaging was performed weekly using the Xenogen IVIS 100 (Caliper LifeSciences) as described previously (Knight et al., 2013). Mammary tumor growth was monitored by twice-weekly caliper measurements. Further details can be found in the Supplemental Experimental Procedures.

Microscopy

Phase contrast images were taken on an Axiovert 200M for adherent cells and an AxioScope Zoom for tumorspheres (both from Carl Zeiss). Immunofluorescence was imaged on an LSM800 confocal laser-scanning microscope (Carl Zeiss).

YAP/TAZ Localization Assays

Polyacrylamide hydrogels, immunofluorescent staining, and analysis are described in the Supplemental Experimental Procedures. Each experiment was conducted in triplicate. An average of 45 cells was scored per replicate per condition.

PCRs

Total RNA was isolated using the RNeasy mini kit (QIAGEN) and reverse-transcribed using the Transcriptor First Strand cDNA Synthesis Kit (Roche). Real-time PCR was performed as described previously, normalizing to *GAPDH* and *B2M* (human) or *Gapdh*, *Hprt*, and *Rpl13a* (mouse) (Knight et al., 2013). Primers (listed in the Supplemental Experimental Procedures) were designed using Primer3 (http://bioinfo.ut.ee/primer3-0.4.0/).

RHO-A Activity Assays and Actin Stress Fiber Scoring

GST pull-downs and RHOA G-LISA assays (Cytoskeleton) are described in the Supplemental Experimental Procedures. Stress fibers were counted in ImageJ software, assessing a minimum of 12 cells per experiment in 3 experiments.

BioID and Mass Spectrometry

KIBRA constructs were cloned into pSTV2-BirA*-FLAG using Gateway LR clonase (Invitrogen). MDA-MB-231 expressing pSTV2-KIBRA constructs or vector alone were analyzed in biological duplicates. Expression and peptide isolation are described in the Supplemental Experimental Procedures.

DATA AND SOFTWARE AVAILABILITY

The accession number for the raw and normalized aCGH and gene expression microarray data reported in this paper is GEO: GSE417748. The accession numbers for the mass spectrometry data reported in this paper are ProteomeXchange: PXD006608 and MassIVE: MSV000081111.

SUPPLEMENTAL INFORMATION

Supplemental Information includes Supplemental Experimental Procedures, seven figures, and four tables and can be found with this article online at https://doi.org/10.1016/j.celrep.2018.02.095.

ACKNOWLEDGMENTS

We thank Anie Monast and Virginie Pilon for assistance with animal experiments and Dr. Peter Siegel for critical reading of the manuscript. Array-CGH was performed by the UC Davis Comprehensive Cancer Center Genomics Shared Resource (Cancer Center Support Grant P30CA093373 from the NCI). Proteomics was performed at the Network Biology Collaborative Centre at the Lunenfeld-Tanenbaum Research Institute (supported by the Canada Foundation for Innovation, the Ontario Government, Genome Canada, and Ontario Genomics [OGI-139]). We acknowledge funding from Fonds de Recherche du Quebec-Sante (to V.Y.C.S., D.A.d.V., and P.P.C.), a Rosalind Goodman Commemorative Scholarship (to C.D.H.R.), Defi-Canderel and Charlotte and Leo Karassik oncology fellowships (to T.G.), the Canada Research Chairs Program (to C.M. [Advanced Cellular Microenvironments] and A.-C.G. [Functional Proteomics]), the Canadian Cancer Society (#704422 to C.M.), the Cancer Research Society (to A.-C.G. and M.P.), the Canadian Institutes for Health Research (FDN 143301 to A.-C.G. and FDN 143281 to M.P.), the Terry Fox Research Institute (to A.-C.G.), and Worldwide Cancer Research (16-0402 to M.P.).

AUTHOR CONTRIBUTIONS

Conceptualization, J.F.K. and M.P.; Methodology and Investigation, J.F.K., V.Y.C.S., E.K., A.L.C., D.A.d.V., C.D.H.R., P.P.C., R.M.J., P.S.-T., T.G., H.W.S., W.L., S.M.S., D.Z., R.R.D., and H.Z.; Formal Analysis, J.F.K., V.Y.C.S., E.K., A.L.C., D.A.d.V., C.D.H.R., P.P.C., R.M.J., M.-C.G., and A.-C.G.; Data Curation, R.M.J. and A.L.C.; Writing – Original Draft, J.F.K. and M.P.; Writing – Review and Editing, J.F.K., V.Y.C.S., E.K., A.L.C., D.A.d.V., C.D.H.R., P.P.C., R.M.J., P.S.-T., T.G., H.W.S., W.L., S.M.S., D.Z., H.Z., M.-C.G., R.R.D., J.P.G., C.M., A.-C.G., and M.P.; Visualization, J.F.K., V.Y.C.S., E.K., A.L.C., P.P.C., and R.M.J.; Funding Acquisition, M.P.

DECLARATION OF INTERESTS

The authors declare no competing interests.

Received: June 29, 2017
Revised: December 20, 2017
Accepted: February 23, 2018
Published: March 20, 2018

REFERENCES

Al-Mulla, F., AlFadhli, S., Al-Hakim, A.H., Going, J.J., and Bitar, M.S. (2006). Metastatic recurrence of early-stage colorectal cancer is linked to loss of heterozygosity on chromosomes 4 and 14q. J. Clin. Pathol. *59*, 624–630.

Aragona, M., Panciera, T., Manfrin, A., Giulitti, S., Michielin, F., Elvassore, N., Dupont, S., and Piccolo, S. (2013). A mechanical checkpoint controls multicellular growth through YAP/TAZ regulation by actin-processing factors. Cell *154*, 1047–1059.

Bartucci, M., Dattilo, R., Moriconi, C., Pagliuca, A., Mottolese, M., Federici, G., Benedetto, A.D., Todaro, M., Stassi, G., Sperati, F., et al. (2015). TAZ is required for metastatic activity and chemoresistance of breast cancer stem cells. Oncogene *34*, 681–690.

Basu-Roy, U., Bayin, N.S., Rattanakorn, K., Han, E., Placantonakis, D.G., Mansukhani, A., and Basilico, C. (2015). Sox2 antagonizes the Hippo pathway to maintain stemness in cancer cells. Nat. Commun. *6*, 6411.

Baumgartner, R., Poernbacher, I., Buser, N., Hafen, E., and Stocker, H. (2010). The WW domain protein Kibra acts upstream of Hippo in Drosophila. Dev. Cell *18*, 309–316.

Belle, L., Ali, N., Lonic, A., Li, X., Paltridge, J.L., Roslan, S., Herrmann, D., Conway, J.R., Gehling, F.K., Bert, A.G., et al. (2015). The tyrosine phosphatase PTPN14 (Pez) inhibits metastasis by altering protein trafficking. Sci. Signal. *8*, ra18.

Cancer Genome Atlas Network (2012). Comprehensive molecular portraits of human breast tumours. Nature *490*, 61–70.

Cardiff, R.D., Anver, M.R., Gusterson, B.A., Hennighausen, L., Jensen, R.A., Merino, M.J., Rehm, S., Russo, J., Tavassoli, F.A., Wakefield, L.M., et al. (2000). The mammary pathology of genetically engineered mice: the consensus report and recommendations from the Annapolis meeting. Oncogene *19*, 968–988.

Chan, S.W., Lim, C.J., Guo, K., Ng, C.P., Lee, I., Hunziker, W., Zeng, Q., and Hong, W. (2008). A role for TAZ in migration, invasion, and tumorigenesis of breast cancer cells. Cancer Res. *68*, 2592–2598.

Chircop, M. (2014). Rho GTPases as regulators of mitosis and cytokinesis in mammalian cells. Small GTPases *5*, e29770.

Cohn, K.H., Wang, F.S., Desoto-LaPaix, F., Solomon, W.B., Patterson, L.G., Arnold, M.R., Weimar, J., Feldman, J.G., Levy, A.T., Leone, A., et al. (1991). Association of nm23-H1 allelic deletions with distant metastases in colorectal carcinoma. Lancet *338*, 722–724.

Cordenonsi, M., Zanconato, F., Azzolin, L., Forcato, M., Rosato, A., Frasson, C., Inui, M., Montagner, M., Parenti, A.R., Poletti, A., et al. (2011). The Hippo transducer TAZ confers cancer stem cell-related traits on breast cancer cells. Cell *147*, 759–772.

Curtis, C., Shah, S.P., Chin, S.F., Turashvili, G., Rueda, O.M., Dunning, M.J., Speed, D., Lynch, A.G., Samarajiwa, S., Yuan, Y., et al.; METABRIC Group (2012). The genomic and transcriptomic architecture of 2,000 breast tumours reveals novel subgroups. Nature *486*, 346–352.

Denkert, C., Liedtke, C., Tutt, A., and von Minckwitz, G. (2017). Molecular alterations in triple-negative breast cancer-the road to new treatment strategies. Lancet *389*, 2430–2442.

Dupont, S., Morsut, L., Aragona, M., Enzo, E., Giulitti, S., Cordenonsi, M., Zanconato, F., Le Digabel, J., Forcato, M., Bicciato, S., et al. (2011). Role of YAP/TAZ in mechanotransduction. Nature *474*, 179–183.

Engler, A.J., Sen, S., Sweeney, H.L., and Discher, D.E. (2006). Matrix elasticity directs stem cell lineage specification. Cell *126*, 677–689.

Foulkes, W.D., Smith, I.E., and Reis-Filho, J.S. (2010). Triple-negative breast cancer. N. Engl. J. Med. *363*, 1938–1948.

Ganem, N.J., Cornils, H., Chiu, S.Y., O'Rourke, K.P., Arnaud, J., Yimlamai, D., Théry, M., Camargo, F.D., and Pellman, D. (2014). Cytokinesis failure triggers hippo tumor suppressor pathway activation. Cell *158*, 833–848.

Genevet, A., Wehr, M.C., Brain, R., Thompson, B.J., and Tapon, N. (2010). Kibra is a regulator of the Salvador/Warts/Hippo signaling network. Dev. Cell *18*, 300–308.

Herschkowitz, J.I., Simin, K., Weigman, V.J., Mikaelian, I., Usary, J., Hu, Z., Rasmussen, K.E., Jones, L.P., Assefnia, S., Chandrasekharan, S., et al. (2007). Identification of conserved gene expression features between murine mammary carcinoma models and human breast tumors. Genome Biol. *8*, R76.

Hill, V.K., Dunwell, T.L., Catchpoole, D., Krex, D., Brini, A.T., Griffiths, M., Craddock, C., Maher, E.R., and Latif, F. (2011). Frequent epigenetic inactivation of KIBRA, an upstream member of the Salvador/Warts/Hippo (SWH) tumor suppressor network, is associated with specific genetic event in B-cell acute lymphocytic leukemia. Epigenetics *6*, 326–332.

Johannsdottir, H.K., Jonsson, G., Johannesdottir, G., Agnarsson, B.A., Eerola, H., Arason, A., Heikkila, P., Egilsson, V., Olsson, H., Johannsson, O.T., et al. (2006). Chromosome 5 imbalance mapping in breast tumors from BRCA1 and BRCA2 mutation carriers and sporadic breast tumors. Int. J. Cancer *119*, 1052–1060.

Knight, J.F., Lesurf, R., Zhao, H., Pinnaduwage, D., Davis, R.R., Saleh, S.M., Zuo, D., Naujokas, M.A., Chughtai, N., Herschkowitz, J.I., et al. (2013). Met synergizes with p53 loss to induce mammary tumors that possess features of claudin-low breast cancer. Proc. Natl. Acad. Sci. USA *110*, E1301–E1310.

Kremerskothen, J., Plaas, C., Büther, K., Finger, I., Veltel, S., Matanis, T., Liedtke, T., and Barnekow, A. (2003). Characterization of KIBRA, a novel WW domain-containing protein. Biochem. Biophys. Res. Commun. *300*, 862–867.

Lehmann, B.D., and Pietenpol, J.A. (2014). Identification and use of biomarkers in treatment strategies for triple-negative breast cancer subtypes. J. Pathol. *232*, 142–150.

Lehmann, B.D., Jovanović, B., Chen, X., Estrada, M.V., Johnson, K.N., Shyr, Y., Moses, H.L., Sanders, M.E., and Pietenpol, J.A. (2016). Refinement of Triple-Negative Breast Cancer Molecular Subtypes: Implications for Neoadjuvant Chemotherapy Selection. PLoS ONE *11*, e0157368.

Levental, K.R., Yu, H., Kass, L., Lakins, J.N., Egeblad, M., Erler, J.T., Fong, S.F., Csiszar, K., Giaccia, A., Weninger, W., et al. (2009). Matrix crosslinking forces tumor progression by enhancing integrin signaling. Cell *139*, 891–906.

Liu, C.Y., Zha, Z.Y., Zhou, X., Zhang, H., Huang, W., Zhao, D., Li, T., Chan, S.W., Lim, C.J., Hong, W., et al. (2010). The hippo tumor pathway promotes TAZ degradation by phosphorylating a phosphodegron and recruiting the SCFbeta-TrCP E3 ligase. J. Biol. Chem. *285*, 37159–37169.

Liu, Y., Chen, C., Xu, Z., Scuoppo, C., Rillahan, C.D., Gao, J., Spitzer, B., Bosbach, B., Kastenhuber, E.R., Baslan, T., et al. (2016). Deletions linked to TP53 loss drive cancer through p53-independent mechanisms. Nature *531*, 471–475.

Lui, C., Lee, K., and Nelson, C.M. (2012). Matrix compliance and RhoA direct the differentiation of mammary progenitor cells. Biomech. Model. Mechanobiol. *11*, 1241–1249.

Moleirinho, S., Chang, N., Sims, A.H., Tilston-Lünel, A.M., Angus, L., Steele, A., Boswell, V., Barnett, S.C., Ormandy, C., Faratian, D., et al. (2013). KIBRA exhibits MST-independent functional regulation of the Hippo signaling pathway in mammals. Oncogene *32*, 1821–1830.

Narumiya, S., Tanji, M., and Ishizaki, T. (2009). Rho signaling, ROCK and mDia1, in transformation, metastasis and invasion. Cancer Metastasis Rev. *28*, 65–76.

Natrajan, R., Lambros, M.B., Rodriguez-Pinilla, S.M., Moreno-Bueno, G., Tan, D.S., Marchio, C., Vatcheva, R., Rayter, S., Mahler-Araujo, B., Fulford, L.G., et al. (2009). Tiling path genomic profiling of grade 3 invasive ductal breast cancers. Clin. Cancer Res. *15*, 2711–2722.

Pece, S., Tosoni, D., Confalonieri, S., Mazzarol, G., Vecchi, M., Ronzoni, S., Bernard, L., Viale, G., Pelicci, P.G., and Di Fiore, P.P. (2010). Biological and molecular heterogeneity of breast cancers correlates with their cancer stem cell content. Cell *140*, 62–73.

Poernbacher, I., Baumgartner, R., Marada, S.K., Edwards, K., and Stocker, H. (2012). Drosophila Pez acts in Hippo signaling to restrict intestinal stem cell proliferation. Curr. Biol. *22*, 389–396.

Ponzo, M.G., Lesurf, R., Petkiewicz, S., O'Malley, F.P., Pinnaduwage, D., Andrulis, I.L., Bull, S.B., Chughtai, N., Zuo, D., Souleimanova, M., et al. (2009).

Met induces mammary tumors with diverse histologies and is associated with poor outcome and human basal breast cancer. Proc. Natl. Acad. Sci. USA *106*, 12903–12908.

Prat, A., and Perou, C.M. (2011). Deconstructing the molecular portraits of breast cancer. Mol. Oncol. *5*, 5–23.

Prat, A., Parker, J.S., Karginova, O., Fan, C., Livasy, C., Herschkowitz, J.I., He, X., and Perou, C.M. (2010). Phenotypic and molecular characterization of the claudin-low intrinsic subtype of breast cancer. Breast Cancer Res. *12*, R68.

Rakha, E.A., Elsheikh, S.E., Aleskandarany, M.A., Habashi, H.O., Green, A.R., Powe, D.G., El-Sayed, M.E., Benhasouna, A., Brunet, J.S., Akslen, L.A., et al. (2009). Triple-negative breast cancer: distinguishing between basal and non-basal subtypes. Clin. Cancer Res. *15*, 2302–2310.

Ren, X.D., Kiosses, W.B., and Schwartz, M.A. (1999). Regulation of the small GTP-binding protein Rho by cell adhesion and the cytoskeleton. EMBO J. *18*, 578–585.

Roux, K.J., Kim, D.I., Raida, M., and Burke, B. (2012). A promiscuous biotin ligase fusion protein identifies proximal and interacting proteins in mammalian cells. J. Cell Biol. *196*, 801–810.

Skibinski, A., Breindel, J.L., Prat, A., Galván, P., Smith, E., Rolfs, A., Gupta, P.B., LaBaer, J., and Kuperwasser, C. (2014). The Hippo transducer TAZ interacts with the SWI/SNF complex to regulate breast epithelial lineage commitment. Cell Rep. *6*, 1059–1072.

Solimini, N.L., Xu, Q., Mermel, C.H., Liang, A.C., Schlabach, M.R., Luo, J., Burrows, A.E., Anselmo, A.N., Bredemeyer, A.L., Li, M.Z., et al. (2012). Recurrent hemizygous deletions in cancers may optimize proliferative potential. Science *337*, 104–109.

Sorrentino, G., Ruggeri, N., Specchia, V., Cordenonsi, M., Mano, M., Dupont, S., Manfrin, A., Ingallina, E., Sommaggio, R., Piazza, S., et al. (2014). Metabolic control of YAP and TAZ by the mevalonate pathway. Nat. Cell Biol. *16*, 357–366.

Takahashi, Y., Miyoshi, Y., Takahata, C., Irahara, N., Taguchi, T., Tamaki, Y., and Noguchi, S. (2005). Down-regulation of LATS1 and LATS2 mRNA expression by promoter hypermethylation and its association with biologically aggressive phenotype in human breast cancers. Clin. Cancer Res. *11*, 1380–1385.

Turner, N., Lambros, M.B., Horlings, H.M., Pearson, A., Sharpe, R., Natrajan, R., Geyer, F.C., van Kouwenhove, M., Kreike, B., Mackay, A., et al. (2010). Integrative molecular profiling of triple negative breast cancers identifies amplicon drivers and potential therapeutic targets. Oncogene *29*, 2013–2023.

Weigman, V.J., Chao, H.H., Shabalin, A.A., He, X., Parker, J.S., Nordgard, S.H., Grushko, T., Huo, D., Nwachukwu, C., Nobel, A., et al. (2012). Basal-like Breast cancer DNA copy number losses identify genes involved in genomic instability, response to therapy, and patient survival. Breast Cancer Res. Treat. *133*, 865–880.

Wilson, K.E., Li, Y.W., Yang, N., Shen, H., Orillion, A.R., and Zhang, J. (2014). PTPN14 forms a complex with Kibra and LATS1 proteins and negatively regulates the YAP oncogenic function. J. Biol. Chem. *289*, 23693–23700.

Xiao, L., Chen, Y., Ji, M., and Dong, J. (2011). KIBRA regulates Hippo signaling activity via interactions with large tumor suppressor kinases. J. Biol. Chem. *286*, 7788–7796.

Xue, W., Kitzing, T., Roessler, S., Zuber, J., Krasnitz, A., Schultz, N., Revill, K., Weissmueller, S., Rappaport, A.R., Simon, J., et al. (2012). A cluster of cooperating tumor-suppressor gene candidates in chromosomal deletions. Proc. Natl. Acad. Sci. USA *109*, 8212–8217.

Yu, J., Zheng, Y., Dong, J., Klusza, S., Deng, W.M., and Pan, D. (2010). Kibra functions as a tumor suppressor protein that regulates Hippo signaling in conjunction with Merlin and Expanded. Dev. Cell *18*, 288–299.

Zanconato, F., Battilana, G., Cordenonsi, M., and Piccolo, S. (2016). YAP/TAZ as therapeutic targets in cancer. Curr. Opin. Pharmacol. *29*, 26–33.

Cell Reports, Volume *22*

Supplemental Information

KIBRA (WWC1) Is a Metastasis Suppressor Gene Affected by Chromosome 5q Loss in Triple-Negative Breast Cancer

Jennifer F. Knight, Vanessa Y.C. Sung, Elena Kuzmin, Amber L. Couzens, Danielle A. de Verteuil, Colin D.H. Ratcliffe, Paula P. Coelho, Radia M. Johnson, Payman Samavarchi-Tehrani, Tina Gruosso, Harvey W. Smith, Wontae Lee, Sadiq M. Saleh, Dongmei Zuo, Hong Zhao, Marie-Christine Guiot, Ryan R. Davis, Jeffrey P. Gregg, Christopher Moraes, Anne-Claude Gingras, and Morag Park

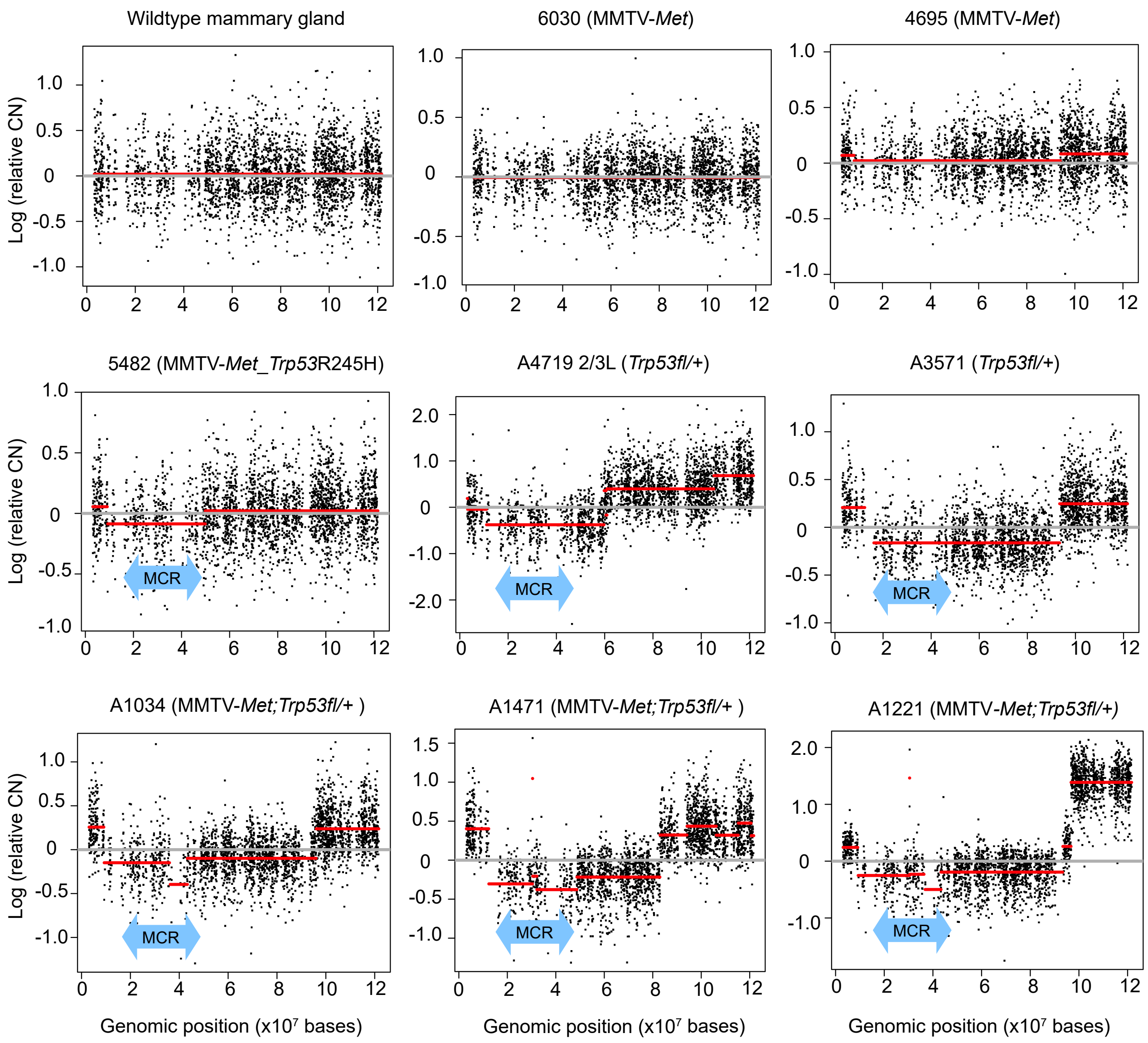

Supplemental Figure S1. Loss of chromosome 11 is a frequent event in MMTV-*Met;Trp53fl/+;Cre* and *Trp53fl/+;Cre* mouse mammary tumors. Refers to figure 1 of the main manuscript.

Examples of array-CGH (aCGH) profiles for mouse chromosome 11. Black dots indicate individual aCGH probes, red lines indicate segmented means for probe regions that deviate from a log copy number change of 0. A profile for chr11 in a normal wildtype mammary gland is shown, alongside profiles for 2 MMTV-*Met* model tumors (6030 and 4695), for which chr11 loss was an infrequent event (8/9 tumors showed no genomic loss). By contrast, loss of chr11 segments occurred frequently in tumors of the MMTV-*Met;Trp53fl/+;Cre* and *Trp53fl/+;Cre* models (18/19 tumors profiled), in addition to 1 MMTV-*Met* tumor with spontaneous *Trp53* mutation (5482). The region of chr11 loss common to all tumors with loss was defined (referred to as the 'minimal common region' or MCR) and is highlighted in blue. This region spans from position chr11:18862572 to 49845204bp.

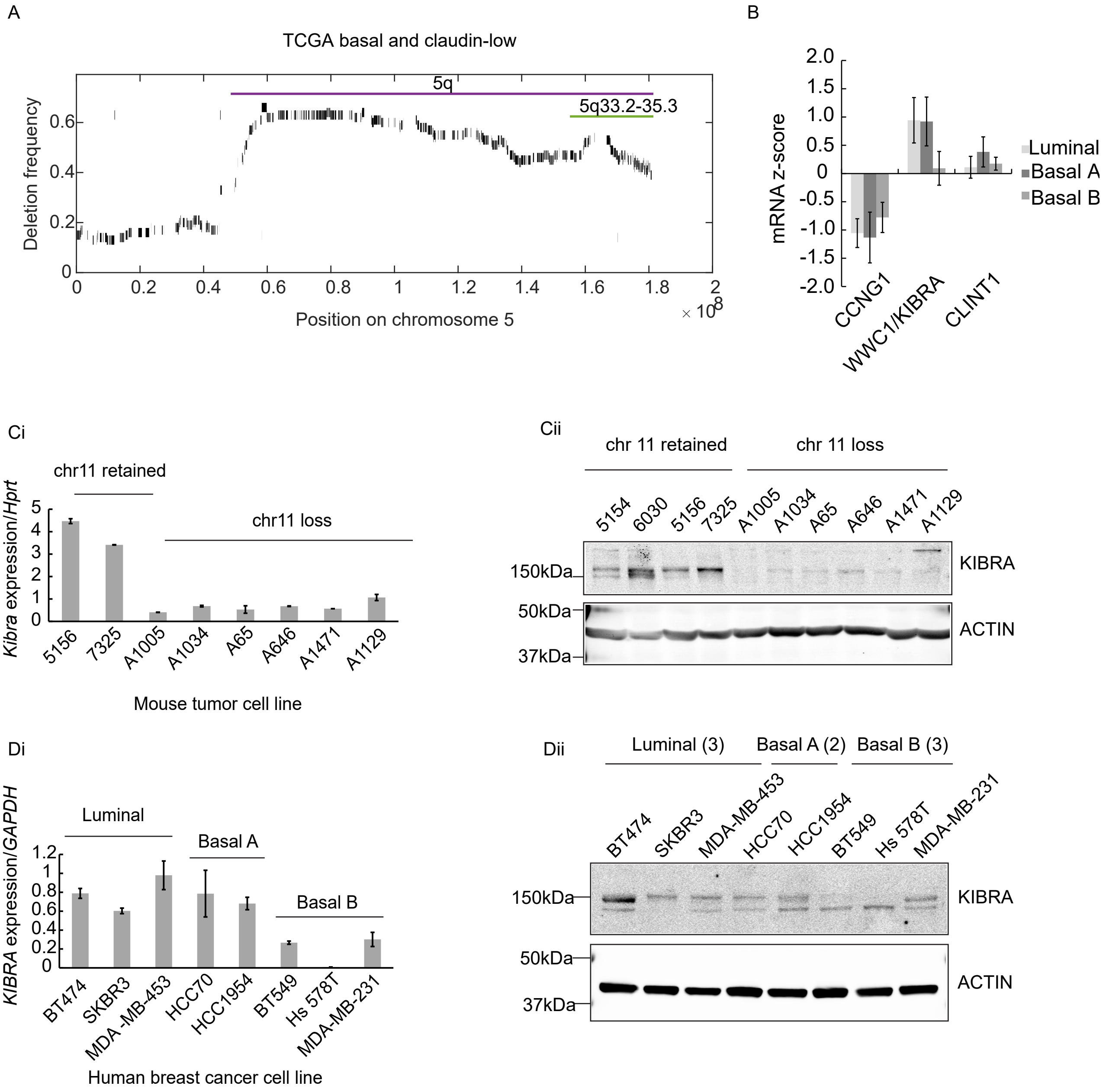

Supplemental Figure S2. Loss of chromosome 5q in basal and claudin-low breast cancers is associated with low expression of *KIBRA*. Relates to figure 1 of the main manuscript.

A) The Cancer Genome Atlas (TCGA) Invasive Breast Carcinoma single nucleotide polymorphism (SNP) array dataset, as analyzed by GISTIC, was used to investigate the frequency of gene loss on chromosome 5 among basal and claudin-low subtypes. Regions spanning the enitre long arm of chr5 (5q) occur in up to 60% of basal and claudin-low tumors. Region 5q33.2-35.3 is highlighted and represents the syntenic region of mouse chr11 that undergoes genomic loss in the transgenic breast cancer models used in this study. Loss of this region occurs in 40-55% of basal and claudin-low tumors. B) The Cancer Cell Line Encyclopedia (CCLE) was used to investigate mRNA expression of 3 genes (*CCNG1, KIBRA, CLINT1*) that undergo hemizygous deletion due to 5q loss (see main Figure 1). Human cell lines representative of the luminal, basal ('basal A') and claudin-low ('basal B') molecular subtypes were analysed. Low expression of *KIBRA* was specifically associated with basal B/claudin-low cell lines Ci) Quantitative real time PCR validated low *Kibra* mRNA expression in mouse mammary tumor cells with genomic loss of the syntenic region on mouse chr11. PCRs were performed in duplicate, error bars are SEM. Cii) Western blotting confirmed absence or low levels of KIBRA protein expression in mouse tumor cells affected by chr11 loss. Di) Quantitative real time PCR validated CCLE data showing reduced expression of *KIBRA* mRNA in basal B breast cancer cell lines compared to other subtypes. PCRs were performed in duplicate, error bars are SEM. Dii) Western blotting showed that KIBRA protein levels were low to absent in human cell lines belonging to the basal B/claudin-low subtype.

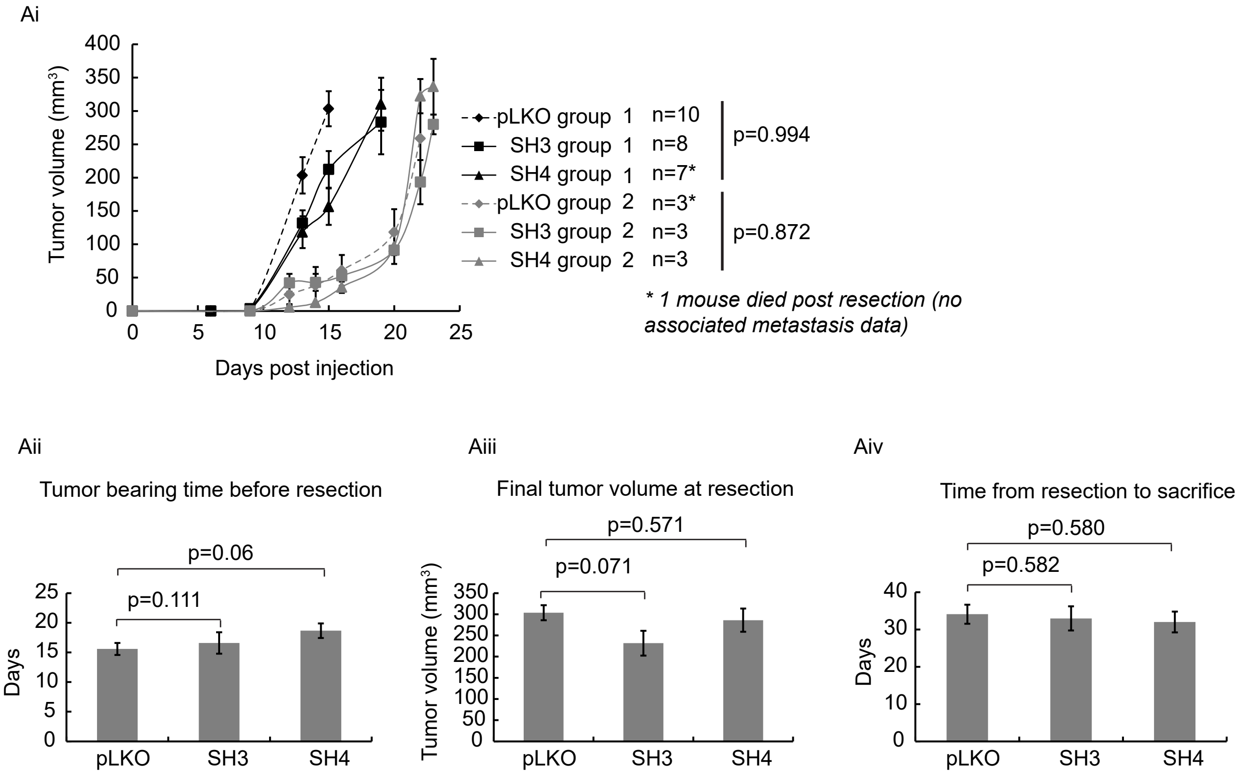

Supplemental Figure S3. Kibra knockdown has no significant impact on the *in vivo* growth of MMTV-*Met* driven mammary tumor cells. Related to Figure 2 of the main manuscript.

Ai) Primary tumour growth curves for mice presented in figure 2. Mammary fat pad injections were performed in nude mice using the MMTV-*Met* mouse mammary tumor cell line 5156-luc. Cells with *Kibra* knockdown (SH3, SH4) are compared to an empty vector control (pLKO). Two experimental groups containing the indicated number of mice are presented, mean values for all mice are shown, +/-SEM. Growth rates of pLKO and SH3/SH4 tumors were not statistically significant as determined by a Kruskal-Wallis One Way Analysis of Variance test. Aii-iv) Primary tumor resection data that accompanies results presented in Figure 2 of the main text. Tumor bearing time prior to resection, final tumor volume at resection and time from resection to sacrifice were equivalent between pLKO control and SH3, SH4 tumors. Mean values for all mice are shown, +/- SEM.

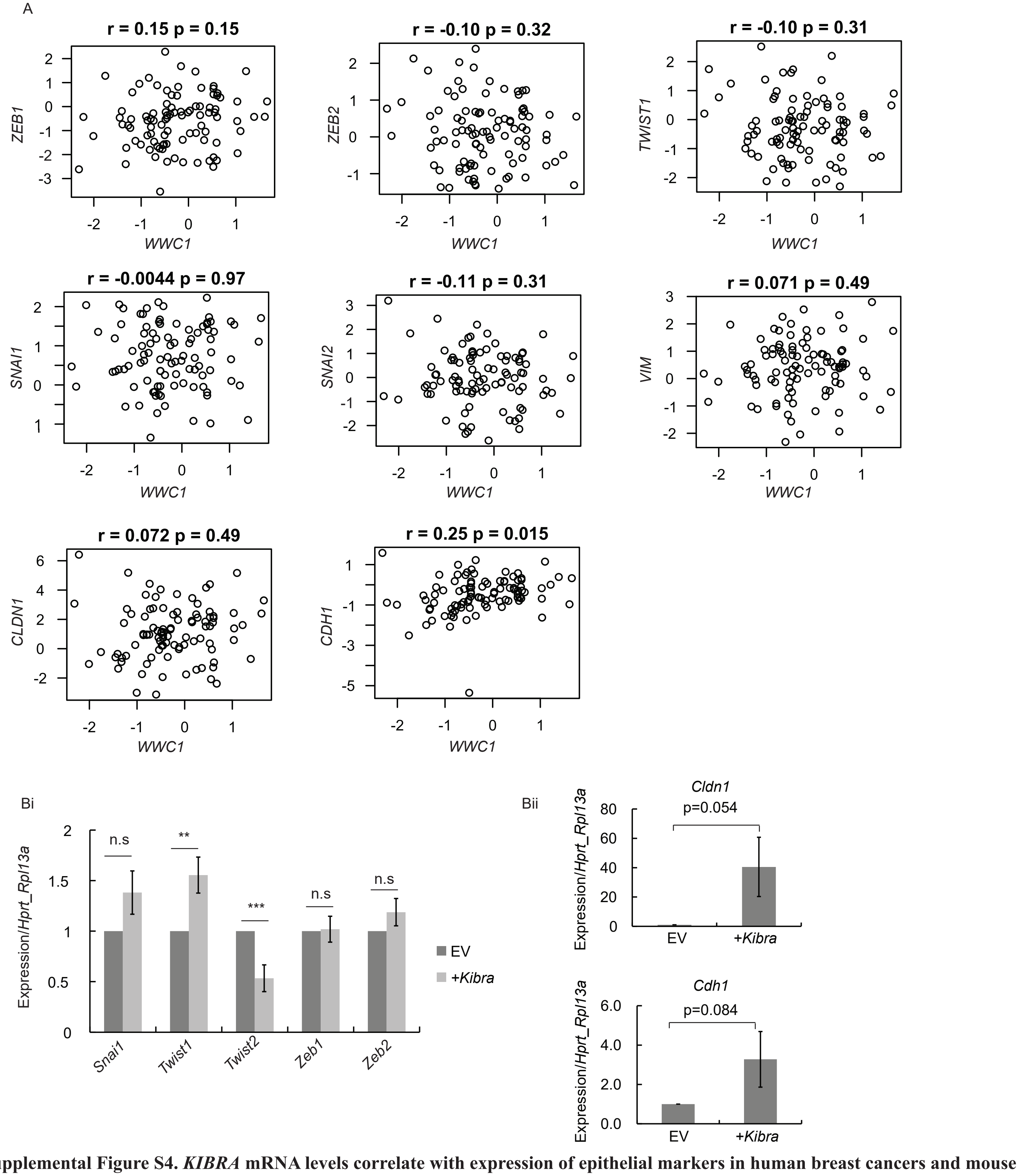

Supplemental Figure S4. *KIBRA* mRNA levels correlate with expression of epithelial markers in human breast cancers and mouse model tumor cells. Relates to figure 3 of the main manuscript.

A) Analysis of gene expression data for pooled basal and claudin-low tumors (TCGA, Nature 2012). Pearson correlation coefficients were calculated to determine the degree of correlation between mRNA levels of *WWC1* (*KIBRA*) and a panel of genes associated with either mesenchymal (*ZEB1/2, TWIST1, SNAI1/2, VIM*) or epithelial (*CLDN1, CDH1*) phenotypes. X and Y axis values are mRNA *Z*-scores. The only significant correlation was with *CDH1* (*E-CADHERIN*). B) RT-PCR data for mouse mammary tumor cells engineered to re-express *Kibra*. EV= empty vector control Bi) The only gene associated with a mesenchymal phenotype to significantly decrease following *Kibra* expression was *Twist2* (p=0.008). *Twist1* showed a compensatory increase (p=0.013). Bii) *Kibra* expression led to increases in the epithelial markers *Cldn1* (*Claudin 1*) and *Cdh1* (*E-Cadherin*). RT-PCR data were normalised to two housekeeping genes (*Hprt* and *Rpl13a*). The mean values for 3 independent experiments using two cell lines (A1005 and A1034) are shown. Error bars are SEM.

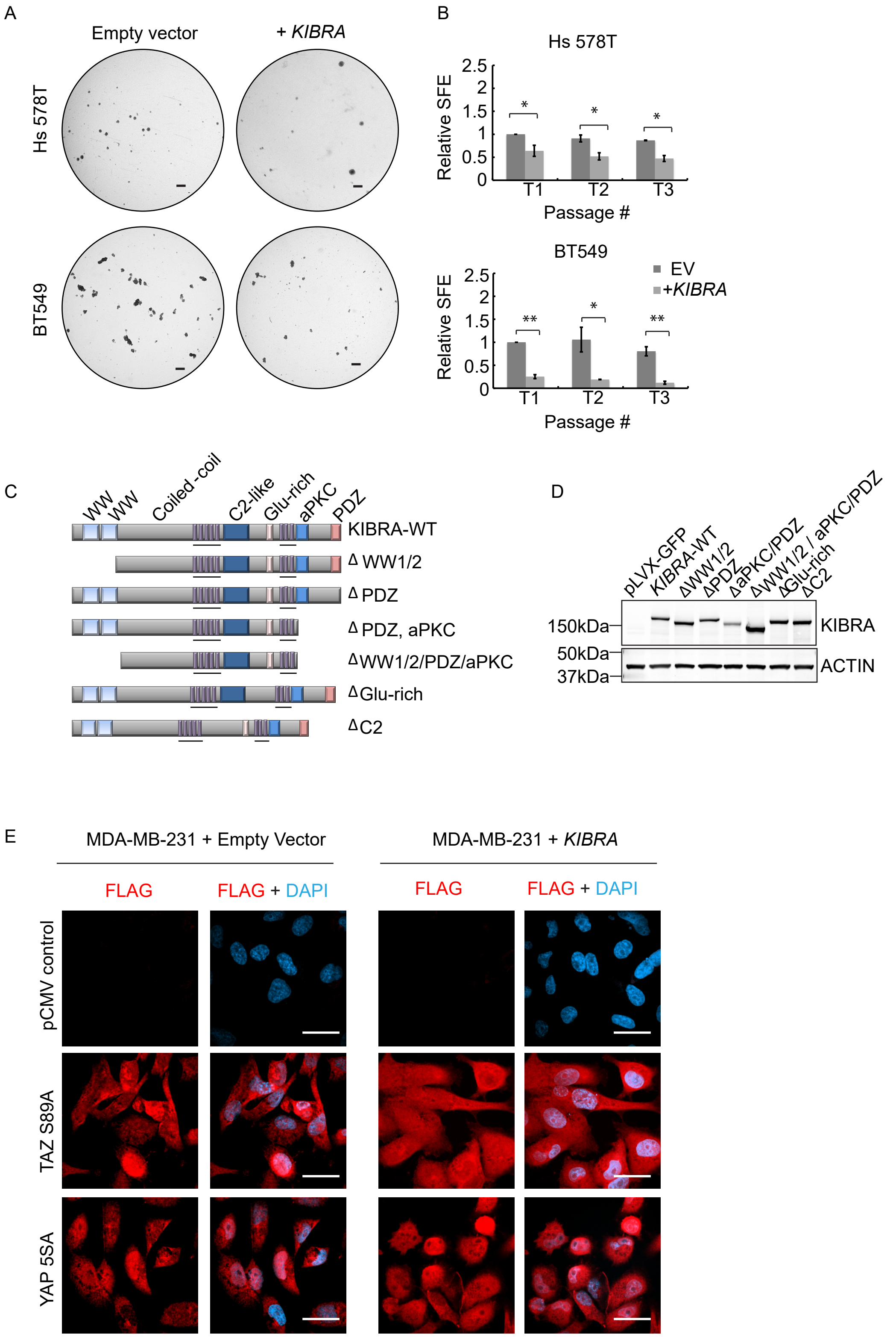

Supplemental Figure S5. ***KIBRA*** **expression impairs tumorsphere formation in human basal B cell lines, a phenotype which requires the KIBRA WW-domains and can be rescued by expression of activated TAZ. Accompanies Figures 4 and 5 of the main manuscript.**

A) Representative images of tumorspheres formed by basal B cell lines Hs 578T and BT549 +/- *KIBRA* expression. Scale bars are 400 µm. B) Quantification of sphere forming efficiency (SFE) for Hs 578T and BT549 cells +/- *KIBRA*. Results mirror those obtained with MDA-MB-231 cells as used in Figure 4 of the main manuscript (3 independent experiments, mean +/- SEM). C) Schematic showing GFP-tagged wildtype KIBRA (KIBRA-WT) and a series of KIBRA mutants lacking protein interaction and structural regions, including the WW-domains shown to be critical for the inhibition of tumorsphere formation (Figure 4) D) Western blotting showing expression of KIBRA-WT and KIBRA mutants in MDA-MB-231 cells (Figure 4). E) Immunofluorescent labelling of MDA-MB-231 cells +/- *KIBRA* and transfected with FLAG-tagged TAZ S89A or YAP 5SA mutants or a pCMV empty vector control (see Figure 5). Nuclear localisation of FLAG confirms TAZ and YAP activity in control and *KIBRA*-expressing MDA-MB-231. Scale bars are 20 µm.

A

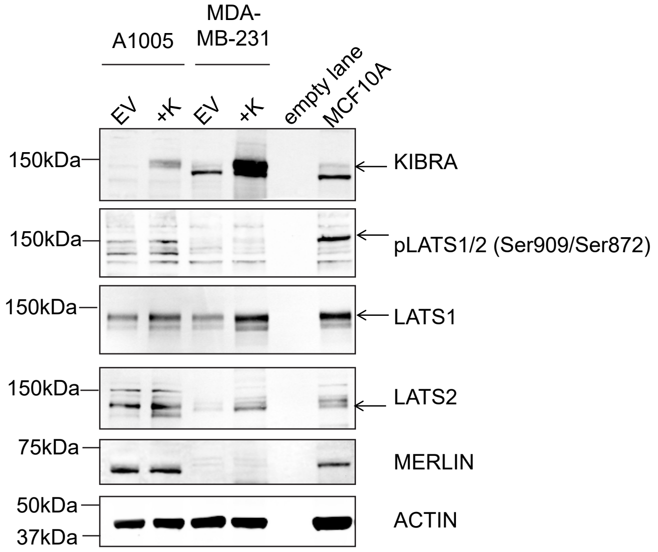

B

Gene (frequency of loss*)		p-value	Log odds ratio	Association
LATS2 (35%)	WWC1 (52%)	<0.001	1.8	Tendency towards co-occurrence
NF2 (17%)	WWC1 (52%)	0.005	2.0	Tendency towards co-occurrence
LATS1 (19%)	WWC1 (52%)	0.015	1.6	Tendency towards co-occurrence

** hetero- or homozygous loss in basal breast cancers (n=81)*

Supplemental Figure S6. MDA-MB-231 cells do not express MERLIN and do not show activation of LATS1/2. Relates to Figure 6 of the main manuscript.
A) Western blots showing expression of Hippo pathway components LATS1, LATS2 and MERLIN in cell lines A1005 and MDA-MB-231, +/- *KIBRA* expression (EV= empty vector control, +K = +*KIBRA*). The immortalised mammary epithelial line MCF10A is used as a positive control for Hippo pathway proteins and correct band sizes are highlighted by arrows. Whilst A1005 express both LATS1 and 2, expression of LATS2 is below the range of detectability in MDA-MB-231 EV cells. However, the presence of KIBRA leads to the stabilisation of LATS1 and 2 in both cell lines. Blotting for the auto-phosphorylation sites using an antibody that reacts with Serine 909 (LATS1) and Serine 872 (LATS2), gave no signal in MDA-MB-231 lysates +/- *KIBRA*, indicating lack of LATS1/2 activation in these cells. In addition, no signal was detected for MERLIN protein in MDA-MB-231. This is in contrast to A1005 cells, which express MERLIN and activate LATS1/2. B) CBioportal output showing the probability of there being co-occurrent deletion of *WWC1* (*KIBRA*) with other genes of the Hippo pathway (*LATS1*, *LATS2* and *NF2/MERLIN*). Analysis was restricted to the basal subtype. Dataset: Breast Invasive Carcinoma (TCGA, Nature 2012). Search terms entered: LATS1:HETLOSS HOMDEL LATS2: HETLOSS HOMDEL WWC1:HETLOSS HOMDEL NF2: HETLOSS HOMDEL. Small sample size (n=8 patients) for the claudin-low subtype precluded analysis of this subset.

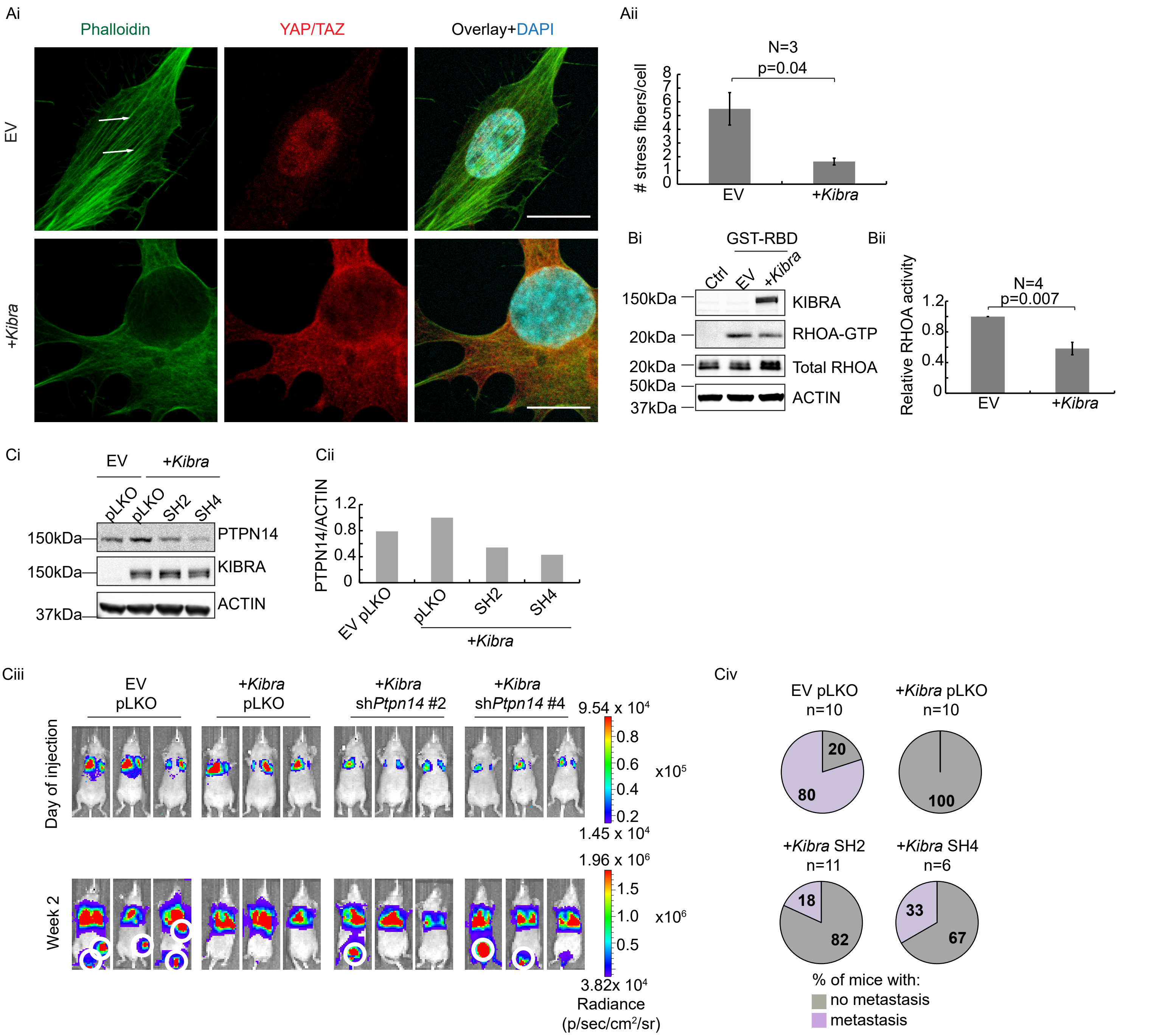

Supplemental Figure S7. *Kibra* expression in A1005 cells disrupts ACTIN stress fibers and inhibits metastatic dissemination, which is partially restored by depletion of *Ptpn14*. Relates to Figure 7 of the main manuscript.

Ai) Subcellular localisation of YAP/TAZ in the mouse mammary tumor cell line A1005 +/- *Kibra* expression. EV=empty vector control. Cells were plated on glass coverslips overlayed with type I collagen to mimic a stiff-matrix environment. Under these conditions, EV cells show nuclear YAP/TAZ and abundant ACTIN stress fibers (white arrows). Expression of *Kibra* leads to accumulation of YAP/TAZ in the cytoplasm and stress fiber loss. Scale bars are 20 µm. Aii) Quantification of (Ai). The mean number of stress fibers per cell is shown. At least 12 cells per experiment were scored and data presented are the mean of 3 independent experiments. Error bars are SEM. Bi) Representative RHO-GST-pulldown using GST-fused to the Rho-binding domain of RHOTEKIN (GST-RBD). Pulldown was performed in A1005 cells +/- *Kibra*. GST-alone is a negative control (Ctrl). Bii) Quantification of RHO-GST pulldowns as shown in (Bi). RHOA activity is based on RHOA-GTP/Total RHOA band intensity. Data are the mean of 4 experiments, error bars are SEM. Ci) Knockdown of *Ptpn14* in A1005 cells +/- *Kibra*. Two independent shRNA oligos (SH2 and SH4) are used in addition to a negative control (pLKO). Cii) Quantification of PTPN14 protein levels shown in (Ci). Levels are normalised to cells expressing *Kibra* with pLKO negative control. Ciii) Representative bioluminescent images of mice injected intravenously with A1005 cells expressing EV or *Kibra* in combination with pLKO or *Ptpn14* shRNA as indicated. White circles indicate metastases outside of the lungs that were confirmed in histological sections. Civ) Percentages of mice with metastases outside of the lungs. Numbers of mice per group are indicated.

Table S1. A summary of published studies in which loss of chromosome 5q has been correlated with basal and triple negative breast cancers. Relates to figure 1 of the main manuscript.

		**Clinical features associated with loss (% of patients affected)**	
**Reference**	**Chr5q region lost (Cytoband)**	**Receptor status**	**Molecular subtype**
(Natrajan et al., 2009)	5q23.1-33.1		Basal (40-44%)
	5q33.2-33.3		
	5q33.3-35.3		
(Bergamaschi et al., 2006)	5q35.3	ER-negative	
	5q11-31		Basal
(Chin et al., 2006)	5q		Basal
(Horlings et al., 2010)	5q33.1	ER-negative	
(Sabatier et al., 2014)	5q		Basal and Claudin-low
(Turner et al., 2010)	5q23.1-34	Triple negative (71%)	
	5q34	Triple negative (43%)	
	5q34-35.3	Triple negative (66%)	
(Wang et al., 2004)	5q11.2-35.1	High grade, ER-neg/ HER2-neg, TP53 mutated	
(Johannsdottir et al., 2006)	5q (20 regions spanning 5q11.2-35.3)		*BRCA1* mutants (31-82%)
(Weigman et al., 2012)	5q11-35		Basal

Table S3. Mouse genes with decreased expression in mammary tumors that display chr11 loss, together with their unique human chr5q homologs. Relates to figure 1 of the main manuscript.

***Mus musculus* gene**	**Chromosome location**	***Homo sapiens* homologue**	**Chromosome location**
Ranbp17	11:33211795-33513746	*RANBP17*	5:170861870-171300015
Gabrp	11:33550781-33578959	*GABRP*	5:170763350-170814047
Foxi1	11:34204338-34208089	*FOXI1*	5:170105897-170109725
Wwc1	11:35838400-35980527	*WWC1*	5:168291651-168472303
Ccng1	11:40748552-40755311	*CCNG1*	5:163437569-163446151
Ccnjl	11:43528784-43586997	*CCNJL*	5:160251652-160345396
Clint1	11:45852051-45910625	*CLINT1*	5:157785743-157859175
BC053393	11:46571536-46589232	*HAVCR1*	5:157029413-157059119
Timd2	11:46668960-46707061	*HAVCR1*	5:157029413-157059119
Havcr1	11:46735080-46779578	*HAVCR1*	5:157029413-157059119
Trim7	11:48826140-48852209	*TRIM7*	5:181193924-181205293
Olfr56	11:48978889-49135387	*OR2V2*	5:181154943-181155890
Olfr1393	11:49280150-49281085	*OR2Y1*	5:180739042-180740099

Thirteen mouse genes located on Chr11 31.4-49.8Mb that undergo hemizygous loss and have decreased gene expression in MMTV-*Met;Trp53fl/+;Cre* and *Trp53fl/+;Cre* mammary tumors. These are homologous with 11 unique human genes (listed) on chromosome 5q.

Experimental Procedures.

Genomic analysis of mouse model tumors. The aCGH data were analysed with the snapCGH version (1.42.0) Bioconductor R package (Smith ML, 2009). The cghMCR (1.30.0) Bioconductor R package was used to determine the minimal common region of loss (Zhang and Feng, 2016). Synteny between the MCR and human 5q was determined by analysis in Ensembl Genome Browser (Aken et al., 2016). Differentially expressed genes (Figure 1C) were identified using the limma Bioconductor package (3.28.21). Gene expression values were scaled by row with *Z*-score shown.

Genomic analysis of basal and claudin-low breast cancers. Patient data were obtained from TCGA (TCGA, 2012), using CBioportal (Gao et al., 2013) to generate the results shown in Figure 1 (D-F). Figure S2A was generated using copy number variations (CNVs) from the same dataset, as obtained from Firehose Broad GDAC (https://gdac.broadinstitute.org/; accessed on 31 July 2016). Frequencies of gene deletions residing on 5q as shown in Figure S2 were derived from the single nucleotide polymorphism array dataset (genome_wide_snp_6-segmented_scna_minus_germline_cnv_hg19) and analyzed by GISTIC2.0 (Mermel et al., 2011). Parameters used for analysis were: reference genome build hg19; amplification threshold 0.3; deletion threshold -0.3; join segment size 4; qv threshold 0.25; remove X chromosome yes; cap value 1.5; confidence level 95; broad analysis yes; broad length cut-off 0.5; maximum samples per segments per sample 2000; arm peel-off yes.

Generation of stable mouse and human cell lines. Selection of cells with stable expression of pBabe-*Kibra* was under Puromycin (2µg/ml). For A1034 and A1005 cells this included the drug efflux inhibitor Cyclosporin A (2.5 µM).

Short hairpin RNAs (shRNAs) targeting mouse *Kibra* in 5156-luc cells were expressed from a pLKO.1 vector (Dharmacon) and the following clones IDs were used: TRCN0000176876 (SH3) and TRCN0000177135 (SH4). Selection was under Puromycin (2µg/ml). Knockdown of *Ptpn14* in A1005 pBabe-EV and pBabe-*Kibra* cells was carried out with shRNAs cloned into 'pLKO.1-Blasticidin' and shRNAs had the following clone IDs: TRCN0000029015 (SH2) and TRCN0000029017 (SH4) (Sigma-Aldrich). Selection with Blasticidin (4µg/ml).

For *in vivo* experiments involving imaging of live mice, A1005 cells expressing pBabe-*Kibra* and 5156 cells with *Kibra* knockdown were transduced with pLenti PGK V5-Luc Neo lentivirus (Addgene 21471; Eric Campeau) and were selected under G418 (400µg/ml).

Expression of N-terminally GFP-tagged wildtype and mutant *KIBRA* in MDA-MB-231 cells was driven from the pLVX lentiviral vector. Cells underwent FACS for GFP-positivity 5 to 7 days post-infection. Sorted cells were cultured for 48 hours prior to use in functional assays.

Knockdown of *PTPN14* in MDA-MB-231 cells was carried out using pLKO.1 encoded shRNAs (Sigma-Aldrich) with the following clone IDs: TRCN0000006890 (SH2); TRCN0000006891 (SH3) and TRCN0000006892 (SH4). *PTPN14* knockdown cells were selected under Puromycin (2µg/ml) and were subsequently infected with pLVX-GFP-KIBRA lentivirus and sorted for GFP-positivity as described above.

Tumorsphere assays. Single cells were seeded in 6-well ultra-low attachments plates (Corning) in 2 ml serum-free DMEM/F12 supplemented with 1x B27, 10 µg/ml insulin (Gibco), 20 ng/ml EGF (BPS Bioscience), 20 ng/ml bFGF (StemRD), 10 µg/ml heparin (StemCell Technologies), and 0.5 µg/ml hydrocortisone (Wisent). 1% Methylcellulose (Sigma) was added to tumorsphere media to prevent cell clumping. BT549 and Hs 578T cells were seeded at 15,000 cells/well, A1005 at 10,000 cells/well and MDA-MB-231 cells at 7500 cells/well. To serially passage, tumorspheres were enzymatically and mechanically dissociated in 0.05% Trypsin-EDTA (Gibco), passed through a 25G needle, and re-seeded as single cells. For experiments involving constitutively active YAP and TAZ constructs, culture medium was changed 24 hours post-transfection. Cells were then trypsinised and resuspended for use in tumorsphere assays, seeding for protein isolation or staining with an anti-FLAG antibody a total of 48 hours post-transfection, as described below.

Proliferation assays. Cells were seeded in quadruplicate wells of a 12 well tissue culture plate (20,000-40,000 cells/well). Cells from one well were trypsinised and counted at 24hr intervals over 96hrs. Counts were performed using the Auto T4 Cellometer (Nexcelom Bioscience).

5156 Migration assays. Cells were plated at $2.5x10^4$ cells per well of a 24-well plate pre-coated with 10ug/ml Fibronectin (EMD Millipore FC010). Cells were allowed to adhere for 4 hours (37 degrees, 5%CO_2) before being transferred to an Axiovert 200M inverted microscope equipped with a motorized stage and Climabox that was maintained at 37 degrees, 5% CO_2 (Carl Zeiss Inc.). AxioVision LE software (Carl Zeiss Inc.) was used to pre-programme positions for imaging. Images were captured every 10 minutes for 8 hours. Cell tracks were generated by manual tracking using MetaMorph software version 7.7.7.0 (Molecular Devices) and analysed using Microsoft Excel.

Geltrex-collagen invasion assays. Twenty-four well plates were pre-treated with 100% ethanol (10 mins) before being coated with Geltrex growth factor-reduced basement membrane matrix (25µl/well) (Life Technologies). Coated plates were incubated at 37°C (30 mins). The cell line 5156-luc was seeded at 5×10^4 cells/well in medium that contained Geltrex (2%) and cultured for 1 day to form cysts. Culture medium was then removed, before adding Type I Human Collagen Solution that had been prepared in PBS and 0.01N NaOH (3mg/ml; 400 µl/well) (Advanced BioMatrix). Plates were then incubated at 37°C (1 hour) before fresh cell culture medium was applied (1ml/well). The cysts were imaged every 15 mins for 48 hours using an Axiovert 200M microscope (Carl Zeiss Inc) as described for migration assays. Cysts with a diameter greater than 0.4 µm at the start were monitored for cell scattering as a measure of invasion into the collagen matrix.

Collagen invasion assays. Assays were performed as detailed elsewhere (Brekhman and Neufeld, 2009) with minor modifications as follows. Type I Human Collagen Solution (Advanced BioMatrix), prepared to 3mg/ml in PBS and 0.01N NaOH with no additional supplements, was used. Both cell lines were seeded in between the two layers of collagen at $5x10^4$ / chamber of an 8-well chamber slide (Nunc). Cells were grown for 7 days prior to fixation. Fixed collagen gels were embedded in OCT and immediately frozen in liquid nitrogen. Constructs were flipped by 90-degrees and sectioned at 8µm thickness. Sections were mounted on microscope slides pre-coated with Poly-L-Lysine (Sigma, P8920) as described by the manufacturer. Sections were dried and nuclei stained with DAPI prior to mounting with a glass coverslip. Sections were imaged at 4X magnification using an EVOS Cell Imaging System. Invasion of individual cells into collagen was quantified using MetaMorph software (Molecular Devices). Briefly, a line was drawn to indicate the plane where cells were seeded and was used to generate an Euclidean distance map. Nuclei that had been identified by the 'granularity' application were transferred onto the Euclidean map to calculate the distance invaded into the collagen either side of the line. The mean distance of all nuclei was calculated. Three images were analysed per condition and 3 independent experiments were performed.

In vivo assays. Sh*Kibra* resection experiments were performed in 6-week old female Athymic nude mice (Taconic Farms, Inc.). Cells were injected into the 4th mammary gland (MFP4-Left; $1x10^6$ cells/mouse) and mammary tumors surgically resected before reaching $500mm^3$. Bioluminescent signal due to re-growth at or near to the primary resection site was excluded in scoring of metastatic burden. Lung metastases were scored in Haematoxylin and Eosin (H&E) stained sections as described (Knight et al., 2013).

A1005 cells expressing pBabe-empty vector or pBabe-*Kibra* were injected orthotopically into 6 week old female FVB/N mice bred in-house (MFP4-Left; $5x10^4$ cells/mouse).

Intravenous (tail vein) injections of A1005-luciferase expressing cells were performed in 6 week old female Athymic nude mice (Taconic Farms, Inc.) as described previously (Knight et al., 2013), injecting $0.5x10^6$ cells per mouse.

Tissue processing and Immunohistochemistry. Mouse tissue was fixed and processed as described (Knight et al., 2013). Haematoxylin and Eosin stained sections of mammary tumor were evaluated with the help of a pathologist (M.C.G) and were imaged using an Aperio-XT slide scanner (Aperio Technologies). The extent of polyploidy was evaluated by manually counting the number of cells exhibiting karyomegaly (enlarged nuclei) or the presence of multiple nuclei in one cell body. One section from each tumor (n=9 EV; n=10 +*Kibra*) was evaluated in Aperio ImageScope software and the entirety of each section was scored. Immunohistochemical staining was carried out using the Discovery Ultra Autostainer (Ventana Medical Systems Inc). Specific antibodies for YAP (Cell Signaling 14074) or TAZ (Atlas Antibodies HPA0077415) were used at 1/400 and 1/200 dilution, respectively. Stained tissue sections were imaged using an Aperio-XT slide scanner (Aperio Technologies). Staining was quantified using HALO 2.0 analysis software (Indica Labs) and the algorithm 'Cytonuclear'.

***KIBRA* mutagenesis.** Mutagenesis was performed on a Gateway Entry vector encoding wildtype *KIBRA* (pENTR11-wildtype *KIBRA*) using the primers indicated below. *KIBRA* constructs were transferred by Gateway LR clonase recombination (Invitrogen) into the acceptor pLVX-GFP lentiviral vector. Constructs were transformed into Stbl3 cells at 30°C to avoid spontaneous recombination prior to DNA purification. The presence of the correct inserts was verified by restriction enzyme digestion with *Mun* I (Fermentas) and by Sanger sequencing.

Mutagenesis primers.

(ΔWW1/2): 5'-TGGCGGCGGGAGCAGGAA-3' and 5'-CATCCATGGGAAGCCTGCTTTTTTGTAC-3'

(ΔPDZ): 5'-TAAGCGGCCGCACTCGAG-3' and 5'-GAGAGCTGGGATATTCATCCGAG-3'

(ΔaPKC/PDZ): 5'-TAAGCGGCCGCACTCGAG-3' and 5'-GGAGCTGTCACTATCACTCCG-3'

(ΔGlu-rich): 5'-AAAGCCTCACCTGATATGG-3' and 5'-CAGTGTCTGTGTGCTGCTC-3'

(ΔC2): 5'-CTCAGCTACAAATACTTGAAG-3' and 5'-CGATTCGTCACTGTCAAATG-3'

PCR primers

Human *CTGF* Fwd: 5'-GCAGGCTAGAGAAGCAGAGC-3'

Human *CTGF* Rvs: 5'-TGGAGATTTTGGGAGTACGG-3'.

Human *CYR61* Fwd: 5'-GGAAAAGGCAGCTCACTGAA-3'.

Human *CYR61* Rvs: 5'-GAGCACTGGGACCATGAAGT-3'.

Human *B2M* Fwd: 5'-TGACTTTGTCACAGCCCAAG-3'.

Human *B2M* Rvs: 5'-AGCAAGCAAGCAGAATTTGG-3'.

GAPDH Fwd: 5'-CTGCACCACCAACTGCTTAG-3'.

GAPDH Rvs: 5'- GTCTTCTGGGTGGCAGTGAT-3'.

Human *KIBRA* Fwd: 5'-GCCTCACCTGATATGGATGG-3'.

Human *KIBRA* Rvs: 5'-CCACTCTCCGGTCCTTAGGT-3'.

Mouse *Kibra* Fwd: 5'-AAGATACCGGCTGGAGGAAC-3'.

Mouse *Kibra* Rvs: 5'- AGCGGACACACAGGCTACTT-3'.

Mouse *Hprt* Fwd: 5'-GCCCCAAAATGGTTAAGGTT-3'.

Mouse *Hprt* Rvs: 5'- CAAGGGCATATCCAACAACA-3'.

Mouse *Rpl13a* Fwd: 5'-AAGGCCAAGATGCACTATCG-3'

Mouse *Rpl13a* Rvs: 5'-GAGTCCGTTGGTCTTGAGGA-3'

Mouse *Zeb1* Fwd: 5'-TGAAGGTGATCCAGCCAAAC-3'

Mouse *Zeb1* Rvs: 5'-GGCGTGGAGTCAGAGTCATT-3'

Mouse *Zeb2* Fwd: 5'-TGGCCTATACCTACCCAACG-3'

Mouse *Zeb2* Rvs: 5'-GTGCTCCATCCAGCAAGTCT-3'

Mouse *Snai1* Fwd: 5'-CTTGTGTCTGCACGACCTGT-3'

Mouse *Snai1* Rvs: 5'-GCAGTGGGAGCAGGAGAAT-3'

Mouse *Twist1* Fwd: 5'-CTCGGACAAGCTGAGCAAG-3'

Mouse *Twist1* Rvs: 5'-CAGCTTGCCATCTTGGAGTC-3'

Mouse *Twist2* Fwd: 5'-ATGTCCGCCTCCCACTAGC-3'

Mouse *Twist2* Rvs: 5'-GTCATGAGGAGCCACAAGGT-3'

Mouse *Cdh1* Fwd: 5'-GACGCTGAGCATGTGAAGAA-3'

Mouse *Cdh1* Rvs: 5'-CAGGACCAGGAGAAGAGTGC-3'

Mouse *Cldn1* Fwd: 5'-ATTGGCATGAAGTGCATGAG-3'

Mouse *Cldn1* Rvs: 5'-CCACTAATGTCGCCAGACCT-3'

YAP/TAZ localisation assays

Tunable elastic modulus cell culture substrate fabrication: Polyacrylamide (PA) hydrogels were polymerized on 12-mm-diameter coverslips, using an adapted protocol (Tse and Engler, 2010). Acrylamide 3% and bisacrylamide 0.059% were used to generate E ~ 0.3 kPa PA gels. Acrylamide 7.5% and bisacrylamide 0.236% were used to generate E ~ 17 kPa PA gels. Sulfo-SANPAH (0.05 mg/ml, G-Biosciences BC38) was added to PA gels and activated by ultraviolet irradiation for 4 min. PA gels were washed with phosphate-buffered saline (PBS) and then functionalized with type I collagen (0.05 mg/ml, Corning, Bovine 354231) overnight at 4°C. Gels were rinsed with PBS and irradiated with UV for 45 min immediately before cell culture.

Elastic modulus measurements: The stiffness of polyacrylamide hydrogels was mechanically characterized by rheometric analysis, using a parallel plate shear rheometer (Anton-Paar). One-millimeter thick polyacrylamide hydrogels were fabricated between two 3-(trimethoxysilyl)propyl methacrylate (MPS) treated coverslips and adhesively fixed between the rheometer plates. The storage modulus (G) was measured at a 5% strain and at 10 Hz, which was verified to be within the linear elastic regime by a strain sweep. The elastic modulus E was estimated to be E=3G, assuming incompressible material properties.

YAP/TAZ immunofluorescence staining: 80,000 cells were plated on 0.3 kPa PA gels and 10,000 on 17 kPa PA gels and coverslips coated directly with type 1 collagen without PA gel (denoted as 70 GPa (Seal A, 2001)) to ensure similar cell density at final time point. Cells were cultured for 72 hrs and then fixed in 4% paraformaldehyde (20 min), permeabilized with 0.2% Triton X-100 (10 min), blocked with 2% BSA (30 min), and then incubated with YAP/TAZ primary antibody (1:200, Cell Signaling 8418) (1 hr). The primary antibody was visualized with a fluorescent secondary antibody conjugated to Alexa Fluor 647 raised in donkey (1:1000, Invitrogen A-31573) together with Alexa Fluor 488 phalloidin (1:200, Invitrogen A12379) (1 hr). Nuclei were counterstained with 0.25 ng/ml DAPI (5 min). All steps were performed at room temperature. Images were acquired on the LSM800 confocal laser scanning microscope (Carl Zeiss, GmbH), using a 20X objective.

Scoring of YAP/TAZ localisation: The MetaXpress Cell Scoring Application was used to calculate ratios of mean fluorescence intensity in the nuclear vs. cytoplasmic compartments of segmented cells (Lin et al., 2015). Thresholds of ratio that were used to establish nuclear and cytoplasmic classes were derived based on precision-recall (PR) analysis using a true positive (TP) set of objects representing distinct subcellular localizations based on MDA-MB-231 expressing pLVX-empty vector across different matrix conditions as scored by visual inspection (Vizeacoumar et al., 2010; Zanella et al., 2007). For example, nuclear TP objects were those for which manual labels were nuclear. Precision was calculated as the fraction of cells correctly classified as nuclear compared to all cells that fall above a

given threshold, i.e. TP/(TP+FP) and recall was calculated as fraction of cells correctly classified as nuclear compared to all cells that are labelled as nuclear, i.e. TP/(TP+FN). Classification of YAP/TAZ localization in KIBRA mutants was achieved with P = 0.91, R = 0.82 for nuclear localization and with P = 0.76 and R = 0.88 for cytoplasmic localization. Classification of YAP/TAZ localization in *PTPN14* knockdown conditions was achieved with P = 0.94, R = 0.75 for nuclear localization and with P = 0.71 and R = 0.92 for cytoplasmic localization.

Immunofluorescent staining for FLAG-tagged YAP2 5SA or TAZ S89A constructs . Cells shown in Figure S5E were cultured on collagen coated glass coverslips as described for YAP/TAZ localisation assays. Cells were stained with an anti-FLAG tag antibody at 1/50 (Cell Signaling 14793) using conditions described above for endogenous YAP/TAZ.

Protein isolation and Western blotting. Protein extraction was performed on ice using either 1% Triton buffer (50 mM Hepes at pH 7.5, 150 mM NaCl, 1.5 mM $MgCl_2$, 1 mM EGTA, 10% glycerol, 1% Triton X-100) or RIPA buffer (1M Tris-Cl (pH8.0), 5M NaCl, 10% NP40, 0.5% Na-desoxy-cholate, 10% SDS) containing the following inhibitors: 1 mM phenylmethylsulfonyl fluoride, 1 mM sodium vanadate, 1 mM sodium fluoride, 10 μg/mL aprotinin, and 10 μg/mL leupeptin). Western blotting was performed using NuPAGE Bis-Tris 4-12% gradient gels in MOPS-SDS running buffer (Invitrogen). The LI-COR Odyssey system was used for detection (see below).

Antibodies used in Western blotting. KIBRA primary antibody (Cell Signaling 8774) was used at 1/600, PTPN14 (Cell Signaling 13808) at 1/1000, PTPN14 (R&D Systems MAB4458) at 1/600, MERLIN (Abcam 88957) at 1/2000, YAP at 1/1000 (Cell Signaling 14074), TAZ at 1/1000 (Cell Signaling 4883), pYAP Ser127 at 1/1000 (Cell Signaling 4911), LATS1 at 1/1000 (Cell Signaling 9153), LATS2 at 1/1000 (Bethyl labs A300-479A), pLATS Ser909 at 1/1000 (Cell Signaling 9157), Beta-Actin (Sigma) 1/10,000, and anti-GFP (Invitrogen LifeSciences A6455) at 1/1000. Secondary antibodies were IRDye 800 CW anti-rabbit and IRDye 680RD anti-mouse and were used as described by the manufacturer (LI-COR).

Immunoprecipitation. Co-immunoprecipitation of GFP-KIBRA and PTPN14 was performed using 500ug of protein input. Lysates were pre-cleared using IgA sepharose beads (GE Healthcare) (45 min). Incubation with anti-GFP antibody (Life Technologies A6455 at 1/500) was performed overnight with rocking, followed by incubation with IgA sepharose beads (1 hr). Beads were washed 3 times in 1% Triton lysis buffer (as described above). All steps were performed a 4 degrees. Protein was eluted by boiling in lamelli buffer containing 1mM DTT.

GST-pulldowns. GST-Rhotekin Rho-binding domain (RBD) protein was expressed in the bacterial strain BL21 DE3 pLysS from the vector pGEX2T, using Isopropyl β-D-thiogalactoside (IPTG) (0.5mM) for induction. RBD protein was conjugated to Glutathione-sepharose beads (Ren et al., 1999). Protocols for lysis and pulldown are described elsewhere (Coleman et al., 2001). Pulldowns were performed in triplicate. Pulldowns and whole cell lysates were analysed by Western blotting using a rabbit anti-RHOA antibody (Santa Cruz).

RHOA G-LISAs. The G-LISA RHOA Activation Assay was performed according to the manufacturer's instructions (Cytoskeleton Inc.) in triplicate. Duplicate absorbance readings per sample were measured using a Varioskan (Thermo Electron Corporation) with SkanIt RE software. Equal levels of total RHOA protein were verified by Western blotting of duplicate lysates.

BioID and mass spectrometry.

Lentiviral delivery of BirA-FLAG-KIBRA and biotin labeling*: BioID experiments were performed by lentiviral transduction in MDA-MB-231 cells, using a BirA*-FLAG cloning vector, pSTV2 (Samavarchi-Tehrani, manuscript in preparation), using Gateway cloning. KIBRA coding sequences (wild type and mutations as described above) were subcloned to induce a N-terminal BirA*-FLAG fusion. HEK293T cells (American Type and Tissue Collection, ATCC, Manassas, VA, USA; Cat# CRL-3216) were used for virus production. Briefly, 3 μg of psPAX2 (a gift from Didier Trono, AddGene #12260), 2 μg of VSV-G packaging vectors (a gift from Bob Weinberg, AddGene #8454) and 3 μg of pSTV2 harboring KIBRA were transfected into HEK293T cells using the jetPRIME reagent as per manufacturer's recommendations (Polyplus-transfection SA, Illkirch-Graffenstaden, France). After 10 hrs, media was changed to virus production media. Virus production media consists of DMEM supplemented with 5% heat-inactivated Fetal Bovine Serum (Gibco, ThermoFisher Scientific, Waltham, MA, USA) and 50 U/ml Penicillin-Streptomycin solution (Corning, Manassas, VA, USA); Virus was harvested at 36 hrs post media change. For all

experiments, MDA-MB-231 cells [American Type and Tissue Collection, ATCC, Manassas, VA, USA; Cat# HTB-26™] in a 15 cm dish at approximately 35-40% density were infected with an amount of KIBRA virus optimized to yield 75-85% infection; to enable doxycycline induction of expression, cells were co-infected with a similarly expressed EF1a-rtTA lentivirus. Cells were then grown until ready for the BioID experiment. One 15 cm dish was used for each biological replicate. Biological duplicates were prepared for all experiments (alongside negative controls). Cells at 75% confluence in 15 cm plates were induced with 1 μg/ml doxycycline and treated with 40 μM biotin for 24 hrs. At the end of the induction and labeling phase, cells were washed and harvested in cold PBS and flash-frozen until time of sample processing.

Streptavidin affinity purification and mass spectrometry: The frozen cell pellets were resuspended in ice-cold RIPA buffer (50 mM Tris-HCl (pH 7.5), 150 mM NaCl, 1% NP-40, 1 mM EDTA, 1 mM EGTA, 0.1% SDS, Sigma protease inhibitors P8340 1:500, and 0.5% sodium deoxycholate), supplemented with 250 U benzonase. Pellets were further processed as described (Hesketh et al., 2017). Peptides were analyzed by nano-LCMS using a home-packed 0.75 μm x 10 cm C18 emitter tip (Reprosil-Pur 120 C18-AQ, 3 μm). A NanoLC-Ultra HPLC system (Eksigent) was coupled to an LTQ Orbitrap Velos or Elite (Thermo Fisher Scientific) and samples were analyzed in data-dependent acquisition mode. A 60,000 resolution MS scan was followed by 10 CID MS/MS ion trap scans on multiple charged precursor ions with a dynamic exclusion of 20 s. The LC gradient was delivered at 200 nl/min and consisted of a ramp of 2-35% acetonitrile (0.1% formic acid) over 90 min, 35-80% acetonitrile (0.1% formic acid) over 5 min, 80% acetonitrile (0.1% formic acid) for 5 min, and then 2% acetonitrile for 20 min. This data set consisting of 10 raw files and associated peak list and results files have been deposited in ProteomeXchange (PXD006608) through partner MassIVE (MSV000081111). Raw files were converted to mzXML and mgf files using ProteoWizard 3.0.4468 (Kessner et al., 2008) and analyzed using the iProphet pipeline (Shteynberg et al., 2011) implemented within ProHits (Liu et al., 2010) as follows. The database consisted of the human and adenovirus sequences in the RefSeq protein database (version 57) supplemented with "common contaminants" from the Max Planck Institute (http://141.61.102.106:8080/share.cgi?ssid=0f2gfuB) and the Global Proteome Machine (GPM; http://www.thegpm.org/crap/index.html). The search database consisted of forward and reverse sequences (labeled "gi|9999" or "DECOY"); in total, 72,226 entries were searched. Spectra were analyzed separately using Mascot (2.3.02; Matrix Science) and Comet [2012.01 rev.3 (Eng et al., 2013)] for trypsin specificity with up to two missed cleavages; deamidation (Asn or Gln) and oxidation (Met) as variable modifications; the mass tolerance of the precursor ion was set at +/-12 parts per million (ppm), the fragment ion tolerance at +/- 0.6 amu. The resulting Comet and Mascot results were individually processed by PeptideProphet (Keller et al., 2002) and combined into a final iProphet output using the Trans-Proteomic Pipeline (TPP; Linux version, v0.0 Development trunk rev 0, Build 201303061711). TPP options were as follows: general options were -p0.05 -x20 -d"gi|9999," iProphet options were –ipPRIME, and PeptideProphet options were –OpdP.

Data analysis and visualization: For analysis with SAINTexpress (Teo et al., 2014), only proteins with an iProphet protein probability of >0.95 were considered. Hits were also restricted to those detected with a minimum of 2 unique peptides. Since only two control purifications were included as part of this analysis, we supplemented these controls with controls from the Contaminant Repository for Affinity Purification (CRAPome; controls were selected to model endogenous biotinylation, i.e. no bait, and promiscuous biotinylation, i.e. FLAG-BirA* alone, in two additional cell lines, namely HEK293 and HeLa cells (Mellacheruvu et al., 2013) . These (CC532, CC533, CC537, CC538, CC540, CC5541, CC546, CC547 were used) and the MDA-MB-231 generated here were compressed to two controls, and SAINTexpress analysis was performed. Here, we considered as high-confidence those hits that passed a 0.8 SAINTexpress cutoff. Visualization of the interactions as dot plots was through prohits-viz.lunenfeld.ca (Knight et al., 2017); once a particular prey passes the SAINTexpress threshold for at least one bait, all the quantitative data across all baits are retrieved and displayed. On these dot plots, the color intensity maps to the averaged spectral counts across both replicates (capped at 50 spectral counts), while the size of the circles is proportional to the maximal spectral count value for the bait across all samples analyzed in parallel. The confidence score from SAINTexpress is mapped as the edge color.

Supplemental References.

Aken, B.L., Ayling, S., Barrell, D., Clarke, L., Curwen, V., Fairley, S., Fernandez Banet, J., Billis, K., Garcia Giron, C., Hourlier, T., *et al.* (2016). The Ensembl gene annotation system. Database : the journal of biological databases and curation *2016*.

Bergamaschi, A., Kim, Y.H., Wang, P., Sorlie, T., Hernandez-Boussard, T., Lonning, P.E., Tibshirani, R., Borresen-Dale, A.L., and Pollack, J.R. (2006). Distinct patterns of DNA copy number alteration are associated with different clinicopathological features and gene-expression subtypes of breast cancer. Genes, chromosomes & cancer *45*, 1033-1040.
Brekhman, V., and Neufeld, G. (2009). A novel asymmetric 3D in-vitro assay for the study of tumor cell invasion. BMC cancer *9*, 415.
Chin, K., DeVries, S., Fridlyand, J., Spellman, P.T., Roydasgupta, R., Kuo, W.L., Lapuk, A., Neve, R.M., Qian, Z., Ryder, T.*, et al.* (2006). Genomic and transcriptional aberrations linked to breast cancer pathophysiologies. Cancer cell *10*, 529-541.
Coleman, M.L., Sahai, E.A., Yeo, M., Bosch, M., Dewar, A., and Olson, M.F. (2001). Membrane blebbing during apoptosis results from caspase-mediated activation of ROCK I. Nature cell biology *3*, 339-345.
Eng, J.K., Jahan, T.A., and Hoopmann, M.R. (2013). Comet: an open-source MS/MS sequence database search tool. Proteomics *13*, 22-24.
Gao, J., Aksoy, B.A., Dogrusoz, U., Dresdner, G., Gross, B., Sumer, S.O., Sun, Y., Jacobsen, A., Sinha, R., Larsson, E.*, et al.* (2013). Integrative analysis of complex cancer genomics and clinical profiles using the cBioPortal. Science signaling *6*, pl1.
Hesketh, G.G., Youn, J.Y., Samavarchi-Tehrani, P., Raught, B., and Gingras, A.C. (2017). Parallel Exploration of Interaction Space by BioID and Affinity Purification Coupled to Mass Spectrometry. Methods Mol Biol *1550*, 115-136.
Horlings, H.M., Lai, C., Nuyten, D.S., Halfwerk, H., Kristel, P., van Beers, E., Joosse, S.A., Klijn, C., Nederlof, P.M., Reinders, M.J.*, et al.* (2010). Integration of DNA copy number alterations and prognostic gene expression signatures in breast cancer patients. Clinical cancer research : an official journal of the American Association for Cancer Research *16*, 651-663.
Johannsdottir, H.K., Jonsson, G., Johannesdottir, G., Agnarsson, B.A., Eerola, H., Arason, A., Heikkila, P., Egilsson, V., Olsson, H., Johannsson, O.T.*, et al.* (2006). Chromosome 5 imbalance mapping in breast tumors from BRCA1 and BRCA2 mutation carriers and sporadic breast tumors. International journal of cancer Journal international du cancer *119*, 1052-1060.
Keller, A., Nesvizhskii, A.I., Kolker, E., and Aebersold, R. (2002). Empirical statistical model to estimate the accuracy of peptide identifications made by MS/MS and database search. Analytical chemistry *74*, 5383-5392.
Kessner, D., Chambers, M., Burke, R., Agus, D., and Mallick, P. (2008). ProteoWizard: open source software for rapid proteomics tools development. Bioinformatics *24*, 2534-2536.
Knight, J.D.R., Choi, H., Gupta, G.D., Pelletier, L., Raught, B., Nesvizhskii, A.I., and Gingras, A.C. (2017). ProHits-viz: a suite of web tools for visualizing interaction proteomics data. Nature methods *14*, 645-646.
Knight, J.F., Lesurf, R., Zhao, H., Pinnaduwage, D., Davis, R.R., Saleh, S.M., Zuo, D., Naujokas, M.A., Chughtai, N., Herschkowitz, J.I.*, et al.* (2013). Met synergizes with p53 loss to induce mammary tumors that possess features of claudin-low breast cancer. Proceedings of the National Academy of Sciences of the United States of America *110*, E1301-1310.
Lin, C.H., Pelissier, F.A., Zhang, H., Lakins, J., Weaver, V.M., Park, C., and LaBarge, M.A. (2015). Microenvironment rigidity modulates responses to the HER2 receptor tyrosine kinase inhibitor lapatinib via YAP and TAZ transcription factors. Molecular biology of the cell *26*, 3946-3953.
Liu, G., Zhang, J., Larsen, B., Stark, C., Breitkreutz, A., Lin, Z.Y., Breitkreutz, B.J., Ding, Y., Colwill, K., Pasculescu, A.*, et al.* (2010). ProHits: integrated software for mass spectrometry-based interaction proteomics. Nature biotechnology *28*, 1015-1017.
Mellacheruvu, D., Wright, Z., Couzens, A.L., Lambert, J.P., St-Denis, N.A., Li, T., Miteva, Y.V., Hauri, S., Sardiu, M.E., Low, T.Y.*, et al.* (2013). The CRAPome: a contaminant repository for affinity purification-mass spectrometry data. Nature methods *10*, 730-736.
Mermel, C.H., Schumacher, S.E., Hill, B., Meyerson, M.L., Beroukhim, R., and Getz, G. (2011). GISTIC2.0 facilitates sensitive and confident localization of the targets of focal somatic copy-number alteration in human cancers. Genome biology *12*, R41.
Natrajan, R., Lambros, M.B., Rodriguez-Pinilla, S.M., Moreno-Bueno, G., Tan, D.S., Marchio, C., Vatcheva, R., Rayter, S., Mahler-Araujo, B., Fulford, L.G.*, et al.* (2009). Tiling path genomic profiling of grade 3 invasive ductal breast cancers. Clinical cancer research : an official journal of the American Association for Cancer Research *15*, 2711-2722.
Ren, X.D., Kiosses, W.B., and Schwartz, M.A. (1999). Regulation of the small GTP-binding protein Rho by cell adhesion and the cytoskeleton. The EMBO journal *18*, 578-585.

Sabatier, R., Finetti, P., Guille, A., Adelaide, J., Chaffanet, M., Viens, P., Birnbaum, D., and Bertucci, F. (2014). Claudin-low breast cancers: clinical, pathological, molecular and prognostic characterization. Molecular cancer *13*, 228.
Seal A, D.A., Banerjee M, Mukhopadhyay AK, Phani KK (2001). Mechanical properties of very thin cover slip glass disc. Bulletin of Materials Science *24*, 151-155.
Shteynberg, D., Deutsch, E.W., Lam, H., Eng, J.K., Sun, Z., Tasman, N., Mendoza, L., Moritz, R.L., Aebersold, R., and Nesvizhskii, A.I. (2011). iProphet: multi-level integrative analysis of shotgun proteomic data improves peptide and protein identification rates and error estimates. Molecular & cellular proteomics : MCP *10*, M111 007690.
Smith ML, M.J., McKinney S, Hardcastle T and Thorne NP (2009). snapCGH: Segmentation, normalisation and processing of aCGH data. R-package version 1.46.0.
TCGA (2012). Comprehensive molecular portraits of human breast tumours. Nature *490*, 61-70.
Teo, G., Liu, G., Zhang, J., Nesvizhskii, A.I., Gingras, A.C., and Choi, H. (2014). SAINTexpress: improvements and additional features in Significance Analysis of INTeractome software. Journal of proteomics *100*, 37-43.
Tse, J.R., and Engler, A.J. (2010). Preparation of hydrogel substrates with tunable mechanical properties. Current protocols in cell biology *Chapter 10*, Unit 10 16.
Turner, N., Lambros, M.B., Horlings, H.M., Pearson, A., Sharpe, R., Natrajan, R., Geyer, F.C., van Kouwenhove, M., Kreike, B., Mackay, A.*, et al.* (2010). Integrative molecular profiling of triple negative breast cancers identifies amplicon drivers and potential therapeutic targets. Oncogene *29*, 2013-2023.
Vizeacoumar, F.J., van Dyk, N., F, S.V., Cheung, V., Li, J., Sydorskyy, Y., Case, N., Li, Z., Datti, A., Nislow, C.*, et al.* (2010). Integrating high-throughput genetic interaction mapping and high-content screening to explore yeast spindle morphogenesis. The Journal of cell biology *188*, 69-81.
Wang, Z.C., Lin, M., Wei, L.J., Li, C., Miron, A., Lodeiro, G., Harris, L., Ramaswamy, S., Tanenbaum, D.M., Meyerson, M.*, et al.* (2004). Loss of heterozygosity and its correlation with expression profiles in subclasses of invasive breast cancers. Cancer research *64*, 64-71.
Weigman, V.J., Chao, H.H., Shabalin, A.A., He, X., Parker, J.S., Nordgard, S.H., Grushko, T., Huo, D., Nwachukwu, C., Nobel, A.*, et al.* (2012). Basal-like Breast cancer DNA copy number losses identify genes involved in genomic instability, response to therapy, and patient survival. Breast cancer research and treatment *133*, 865-880.
Zanella, F., Rosado, A., Blanco, F., Henderson, B.R., Carnero, A., and Link, W. (2007). An HTS approach to screen for antagonists of the nuclear export machinery using high content cell-based assays. Assay and drug development technologies *5*, 333-341.
Zhang, J., and Feng, B. (2016). cghMCR: Find chromosome regions showing common gains/losses.
